# Genomic insights into biased allele loss and increased gene numbers after genome duplication in autotetraploid *Cyclocarya paliurus*

**DOI:** 10.1186/s12915-023-01668-1

**Published:** 2023-08-08

**Authors:** Rui-Min Yu, Ning Zhang, Bo-Wen Zhang, Yu Liang, Xiao-Xu Pang, Lei Cao, Yi-Dan Chen, Wei-Ping Zhang, Yang Yang, Da-Yong Zhang, Er-Li Pang, Wei-Ning Bai

**Affiliations:** State Key Laboratory of Earth Surface Processes and Resource Ecology, and Ministry of Education Key Laboratory for Biodiversity Science and Ecological Engineering, College of Life Sciences, Beijing Normal University, Beijing, 100875 China

**Keywords:** Allelic chromosome, Autopolyploidy, Shallow divergence, Allele loss, Tetrasomic inheritance, Whole-genome duplication

## Abstract

**Background:**

Autopolyploidy is a valuable model for studying whole-genome duplication (WGD) without hybridization, yet little is known about the genomic structural and functional changes that occur in autopolyploids after WGD. *Cyclocarya paliurus* (Juglandaceae) is a natural diploid–autotetraploid species. We generated an allele-aware autotetraploid genome, a chimeric chromosome-level diploid genome, and whole-genome resequencing data for 106 autotetraploid individuals at an average depth of 60 × per individual, along with 12 diploid individuals at an average depth of 90 × per individual.

**Results:**

Autotetraploid *C. paliurus* had 64 chromosomes clustered into 16 homologous groups, and the majority of homologous chromosomes demonstrated similar chromosome length, gene numbers, and expression. The regions of synteny, structural variation and nonalignment to the diploid genome accounted for 81.3%, 8.8% and 9.9% of the autotetraploid genome, respectively. Our analyses identified 20,626 genes (69.18%) with four alleles and 9191 genes (30.82%) with one, two, or three alleles, suggesting post-polyploid allelic loss. Genes with allelic loss were found to occur more often in proximity to or within structural variations and exhibited a marked overlap with transposable elements. Additionally, such genes showed a reduced tendency to interact with other genes. We also found 102 genes with more than four copies in the autotetraploid genome, and their expression levels were significantly higher than their diploid counterparts. These genes were enriched in enzymes involved in stress response and plant defense, potentially contributing to the evolutionary success of autotetraploids. Our population genomic analyses suggested a single origin of autotetraploids and recent divergence (~ 0.57 Mya) from diploids, with minimal interploidy admixture.

**Conclusions:**

Our results indicate the potential for genomic and functional reorganization, which may contribute to evolutionary success in autotetraploid *C. paliurus*.

**Supplementary Information:**

The online version contains supplementary material available at 10.1186/s12915-023-01668-1.

## Background

Autopolyploids form within a species by whole-genome duplication (WGD) and generally show random segregation of homologous chromosomes [1–3]. Ramsey and Schemske [1] estimated that the frequency of autotetraploid formation is on the same order as the genic mutation rate (10^−5^). Moreover, there is strong evidence that autopolyploids are more common than previously appreciated [4, 5]. Although autotetraploids offer an opportunity to examine the effects of WGD without the confounding impacts of hybridization, research on autopolyploids has lagged behind that on allopolyploids, and many aspects of autopolyploidy evolution remain poorly understood [1, 6–8]. For example, many allopolyploids have been studied to investigate the immediate and long-term effects of polyploidization on genome evolution [9], which include genome structural and functional reorganization over time, such as changes in genome size, genome rearrangements, and alterations in gene expression [2]. However, the information available on these effects in autopolyploid species is limited. One possible reason may be that assembling allele-aware chromosome-level genomes for autotetraploids is very difficult. Fortunately, new genomic approaches and methods hold promise for enabling the assembly of high-quality genomes of autopolyploids and investigating their genome changes [10–12]. Recently, with allele-aware chromosome-level genome data, highly abundant structural rearrangements involving ~ 20% of the genome were detected in an autotetraploid potato cultivar [12], indicating that the genome evolution of autopolyploids may not be as simple as previously considered.

*Cyclocarya paliurus* (wheel wingnut) is the sole species of the genus *Cyclocarya* in the family Juglandaceae and is native to eastern and central China. The species contains a wealth of medicinal compounds, such as polysaccharides, flavones, and triterpenoids, as well as several trace elements [13–17], and has long been used as a traditional Chinese medicine to control human blood glucose and lipid concentrations [14, 18]. The species was reported to have an abnormal number of chromosomes (*x* = 28) [19], whereas all other Juglandaceae species are diploids with 32 chromosomes (2*n* = 2*x* = 32) [19, 20]. The unique chromosome number was considered to be the distinctness of *Cyclocarya* from its sister genus *Pterocarya* [19]. However, Zheng et al. [21] and Qu et al. [22] recently found that *C. paliurus* includes autotetraploid individuals with 64 chromosomes. Therefore, the autotetraploid individuals may previously have been wrongly regarded as diploids with a unique aneuploid chromosome number. Considering that the sister genera *Pterocarya* and *Juglans* are diploid with 2*n* = 2*x* = 32, it is highly possible that *C. paliurus* is a naturally diploid–autotetraploid species [22], providing a powerful basis for investigating autotetraploid origins and genome evolution.

In this study, we used the PacBio CCS (circular consensus sequencing) platform and Hi-C (high-throughput chromosome conformation capture) technology to assemble an allele-aware chromosome-level genome for autotetraploid and a chromosome-level genome for diploid *C. paliurus*. In addition, we generated comprehensive range-wide population genomic data for autotetraploid (106 individuals) and diploid (12 individuals) lineages. The aim was to reveal genomic changes in autotetraploid *C. paliurus* after WGD and to gain new insights into the evolution and potential adaptation of naturally occurring autotetraploids.

## Results

### Genome assembly for autotetraploid and diploid *C. paliurus*

**Fig. 1:**
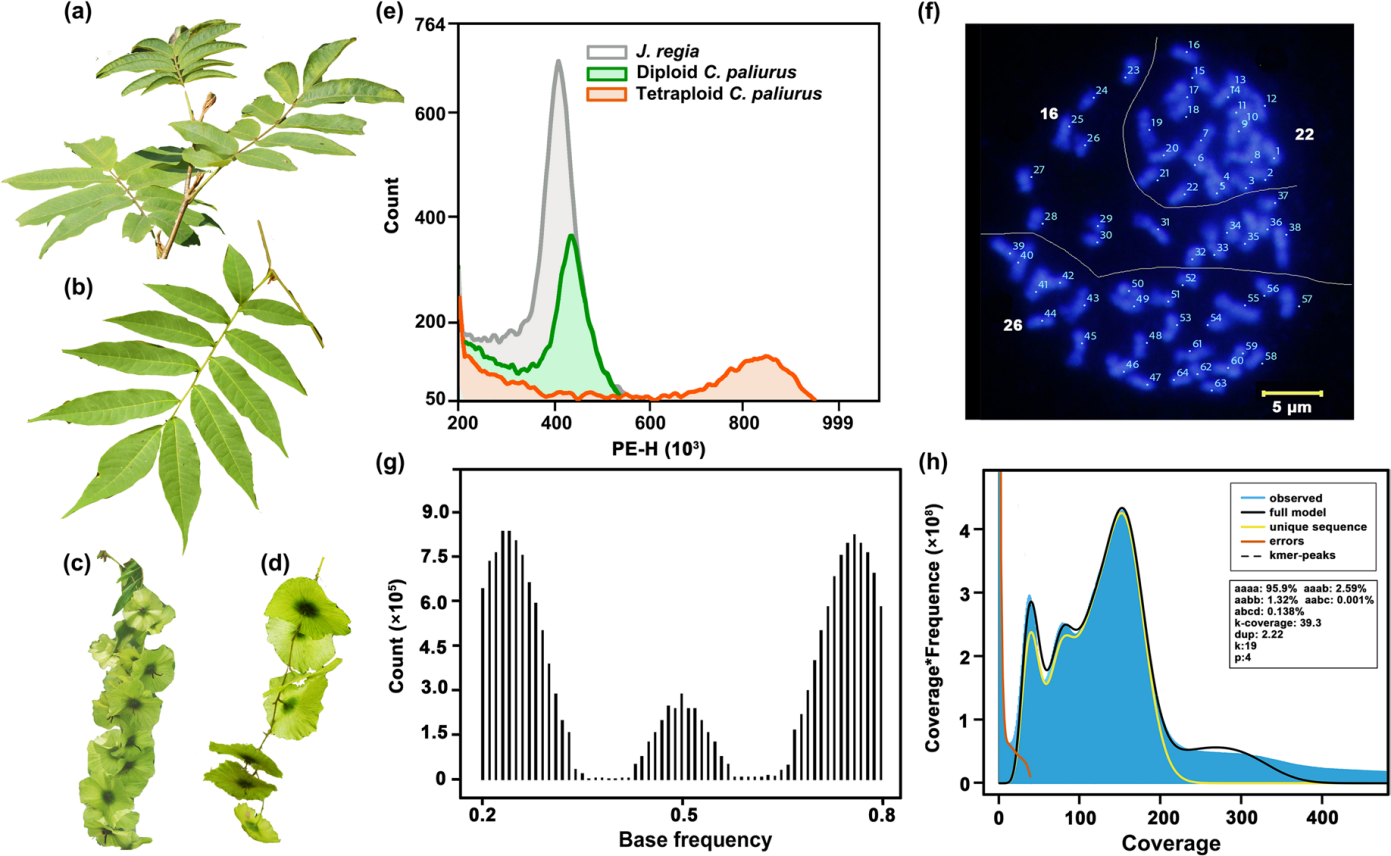
Identification of autotetraploid *C. paliurus.* The mature leaves in **a** and **b**, as well as the fruits in **c** and **d** for both diploid and autotetraploid *C. paliurus*. **e** Flow cytometric histograms showing the relative PI fluorescence intensity in nuclei from leaves of diploid and tetraploid *C. paliurus* and *J. regia*. **f** Mitotic metaphase chromosomes of a tetraploid *C. paliurus*. The blue chromosomes were counterstained with DAPI, and the chromosome number of this tetraploid is 64. Scale bar = 5 μm. **g** Distribution of base frequencies at variable sites determined using nQuire. Three peaks at 0.25, 0.50, and 0.75 indicated that this tetraploid is an autotetraploid. **h** *K*-mer spectra and fitted models for the tetraploid individual

The results of flow cytometry suggested that *C. paliurus* is a naturally diploid–autotetraploid species (Fig. 1a–e). Fluorescence in situ hybridization (FISH) suggested that the chromosome number of a tetraploid individual was 2*n* = 4*x* = 64 (Fig. 1f). The peak of the base frequencies of variable sites for the tetraploid individual were 0.25, 0.5, and 0.75, and a high proportion of one or three copies of a subgenome was observed rather than two copies, indicating that the tetraploid individual of *C. paliurus* was autotetraploid (Fig. 1g, h).

**Fig. 2:**
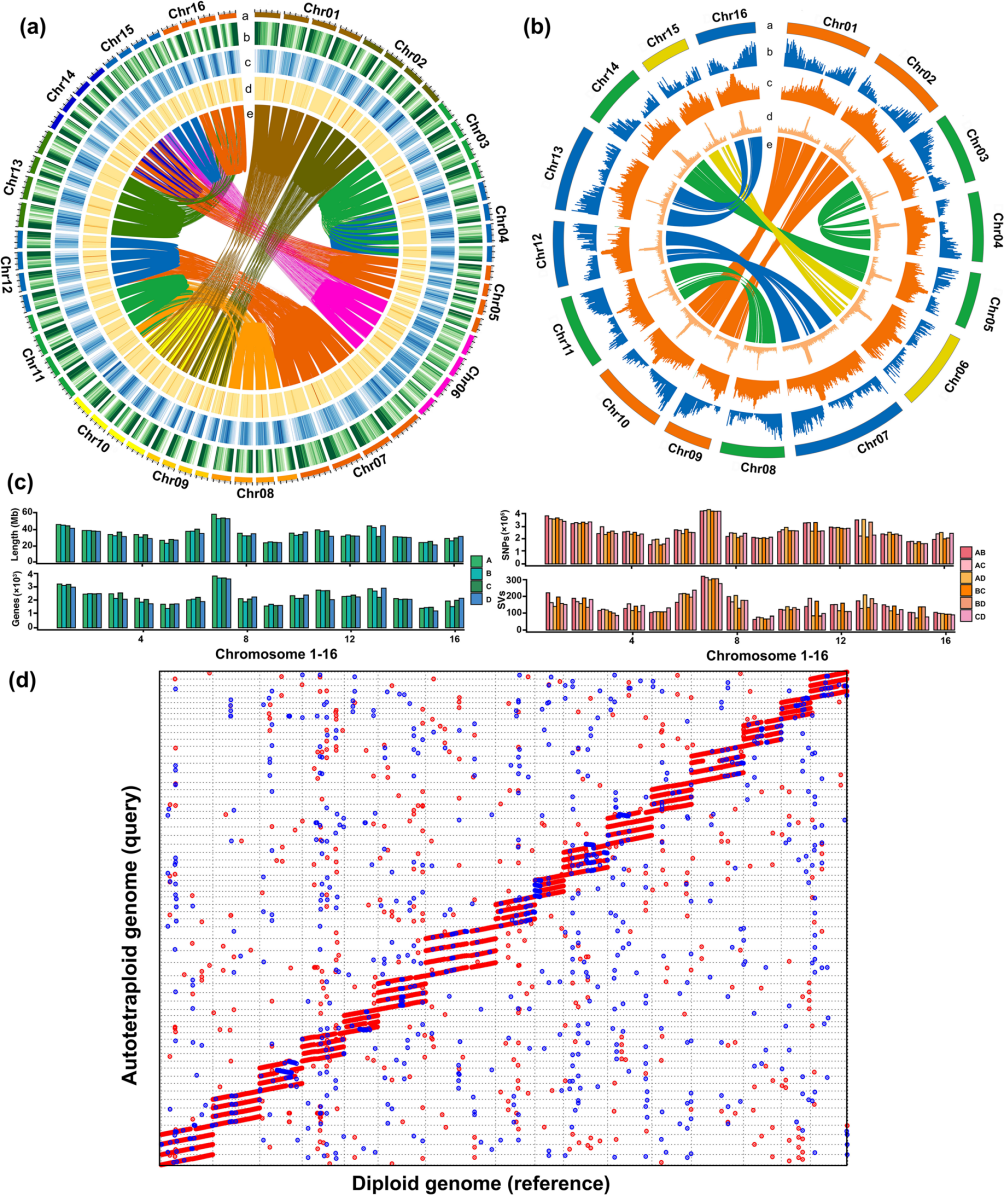
Overview of the *C. paliurus* genome. Circos plots for the autotetraploid (**a**) and diploid genomes (**b**). The tracks are (from outermost to innermost) as follows: “a,” chromosome order, “b,” gene density, “c,” density of transposon elements, “d,” GC content, and “e,” syntenic blocks. **c** Length, number of genes, single-nucleotide polymorphisms (SNPs), and structural variations (SVs) of four monoploid chromosomes. **d** Four monoploid chromosomes were aligned to the diploid chromosome

A total of 73 gigabases (Gb) of CCS long reads were assembled for an autotetraploid *C. paliurus* with a total length of 2.36 Gb and 4.05% heterozygosity, (Table 1). Subsequently, 156 million reads of Hi-C data were utilized to scaffold the autotetraploid genome at the chromosomal level. The assembled genome consisted of 64 chromosomes that constituted 16 homologous groups. The 64 chromosomes anchored 2.25 Gb of the genome, accounting for 95.47% of the total genome size (Fig. 2 and Table 1). The scaffold and contig N50 values were 33.24 and 6.32 Mb, respectively. Several approaches were employed to confirm the accuracy of the assembly. First, Hi-C interactions heatmap showed strong linkages within each homologous chromosome and relatively few linkages between homologous chromosomes, indicating a clear separation of homologous chromosomes of autotetraploid genome (Additional file 1: Fig. S1a). Second, a total of 1563 (96.84%) complete gene in BUSCO and 220 (88.71%) ultraconserved core eukaryotic genes in CEGMA were identified in the autotetraploid *C. paliurus* (Additional file 2: Table S1). Third, we mapped both 262 million next-generation Illumina short reads and five million CCS long reads to the assembled genome, achieving high coverage rates of 96.82 and 99.82% (Additional file 2: Table S2), respectively. The HiFi reads uniformly covered the whole genome (Additional file 1: Fig S2 and S3), and detailed alignment plots were provided on our website. We randomly selected 20 copy gain/loss regions and found effective HiFi read coverage at these regions (Additional file 3). Fourth, we detected 104 breakpoints across the assembled genome using GAEP [23] (Additional file 4), with an average of 0.05 breakpoints per Mb, indicating high structural correctness. Fifth, the annotation of long terminal repeats (LTRs) revealed an LTR Assembly Index (LAI) score of 11.36 met the standard for high-quality reference genome [24] (Additional file 1: Fig. S4). These findings demonstrate we have successfully generated a high-quality and high integrity genome assembly.

**Table 1:** Statistics for autotetraploid and diploid *C. paliurus* genomes

	**Autotetraploid *C. paliurus* genome**	**Diploid *C. paliurus* genome**
Assembly size (bp)	2,355,676,533	601,463,103
Anchoring size (bp)	2,248,910,762	600,846,041
Anchoring rate (%)	95.47	99.90
GC content (%)	36.71	36.79
Number of scaffolds	3010	33
Scaffold N50 size (bp)	33,241,311	38,614,602
Number of contigs	3532	55
Contig N50 size (bp)	6,316,438	25,614,943
Number of genes/alleles	157,337	28,621
Repetitive elements (%)	43.61	55.52
Heterozygosity (%)	4.05	1.11

In total, we identified 157,337 protein-coding genes, 40,337 noncoding RNAs (30,775 rRNAs, 8302 tRNAs, 443 miRNAs, 376 snRNAs, and 441 snRNAs), and 1149 Mb of repetitive sequences (48.76% of the assembled genome, including 6.52% tandem repeats, 28.95% LTR retrotransposons, 5.25% long interspersed nuclear elements, and 7.86% DNA transposons) (Additional file 2: Table S3). The majority of homologous chromosomes demonstrated similarity in length, gene count, single-nucleotide polymorphisms (SNPs), and structural variations (SVs), with the exception of Chr13 and Chr16 (Fig. 2c, d; Additional file 1: Fig. S5; Additional file 2: Tables S4 and S5), suggesting that these genomes have been recently inherited from a shared ancestor.

For comparison, we also assembled the genome for diploid *C. paliurus.* The final assembled genome size was 601.46 Mb, and 99.90% of the genome was oriented into 16 pseudochromosomes (Fig. 2, Additional file 1: Fig. S1b and Table 1). The assembly completeness assessment showed that 1581 (97.96%) complete genes in BUSCO and 222 (89.52%) ultraconserved core eukaryotic genes in CEGMA were identified in diploid *C. paliurus* (Additional file 2: Table S1). In total, we identified 28,621 protein-coding genes, 36,365 noncoding RNAs (34,572 rRNAs, 562 tRNAs, 494 miRNAs, and 737 snRNAs), and 55.52% repetitive elements, including 1.2% tandem repeats and 54.32% transposable elements (TEs) (Additional file 2: Table S6).

### WGD and genome characteristics of autotetraploid *C. paliurus*

Based on previous studies [25, 26], Juglandaceae species have experienced two rounds of ancient WGD events, namely, the *γ*-WGT (~ 120 million years ago, Mya) and the Juglandoid WGD (~ 85 Mya). The synonymous substitution rate (*K*_s_) peak values for the collinear gene pairs were 0.325 and 1.25 for diploid *C. paliurus* and three Juglandinae species (*P. stenoptera*, *J. mandshurica*, and *J. nigra*) (Additional file 1: Fig. 6a), corresponding to the two ancient WGD events. In addition to the two peaks, *K*_s_ values for autotetraploid *C. paliurus* showed a peak at 0.005 (Additional file 1: Fig. 6b), occurring approximately 1.2 Mya if a mutation rate of 2.06 × 10^−9^ was assumed [27]. The synteny plots of diploid and autotetraploid *C. paliurus* revealed that one region in the diploid genome was traced to four regions in the autotetraploid (Additional file 1: Fig. 6c), corresponding to a third WGD event.

**Fig. 3:**
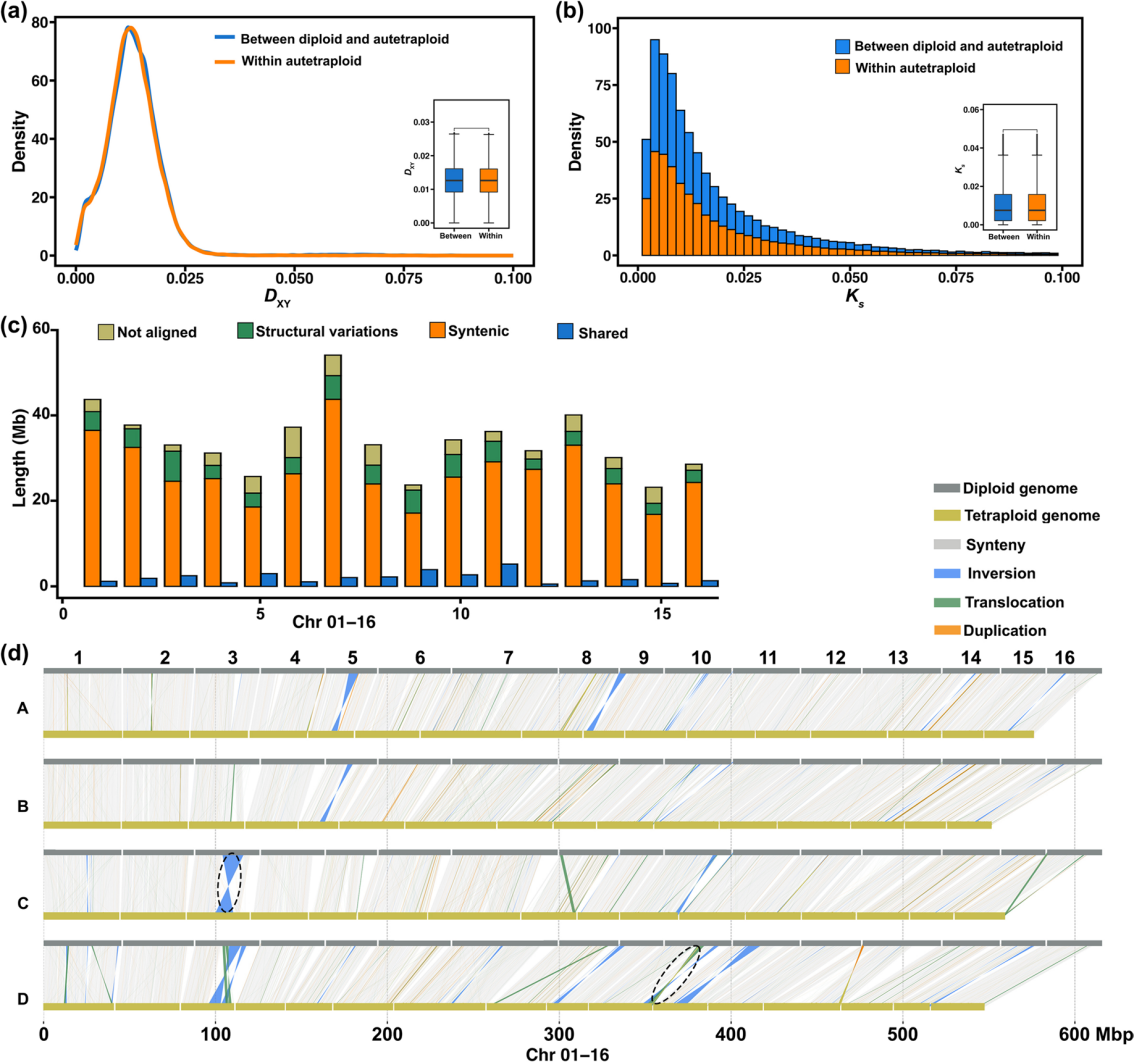
Genome analysis of diploid and autotetraploid *C. paliurus.* **a*** D*_XY_ in 50 kb stepping windows between the diploid and autotetraploid genomes and *D*_XY_ within any two monoploid genomes of the autotetraploid. **b*** K*_s_ between the diploid and autotetraploid genomes and *K*_s_ within any two monoploid genomes of the autotetraploid. **c** Average alignment statistics and structural variations in each chromosome. **d** Structural variations between the chromosomes of the diploid and autotetraploid; A, B, C, and D represent four monoploid genomes. The dotted lines mark the largest inversion and translocation

Between the diploid and autotetraploid genomes, the regions of synteny, structural variation, and nonalignment made up 81.3% (1.92 Gb), 8.8% (191.8 Mb), and 9.9% (233.6 Mb), respectively, of the assembled autotetraploid genome, which were shared by either two, three, or four monoploid genomes. The pairwise nucleotide difference (*D*_XY_) of syntenic regions and *K*_s_ for genes between diploid and autotetraploid chromosomes were similar to *D*_XY_ and *K*_s_ between any two monoploid genomes of an autotetraploid (Fig. 3a–c and Additional file 1: Fig. S7–10). There were 257 inversions (71.4 Mb), 5078 translocations (62.9 Mb), and 10,446 duplications (57.5 Mb), accounting for 3.3, 2.9, and 2.6%, respectively, of the autotetraploid genome (Fig. 3c, d). A total of 48 inversions, 517 translocations, and 1296 duplications were shared by two or more monoploid genomes of the autotetraploid (Additional file 2: Table S7), and harbored 1555, 1147, and 630 genes, respectively. The largest inversion, which reached a size of 12.8 Mb, was unique to Chr03 and harbored 490 genes, which were significantly enriched (14 of the 490 genes) in flavin adenine dinucleotide binding (Fig. 3d and Additional file 1: Fig. S11). The largest translocation, which was 2.5 Mb, was unique to Chr10 and harbored 52 genes, which were significantly enriched (5 of the 52 genes) in cell wall modification (Fig. 3d and Additional file 1: Fig. S11). These results indicated very shallow divergence between the diploid and autotetraploid genomes.

### Allele-specific annotation and biased allele loss of autotetraploid *C. paliurus*

**Fig. 4:**
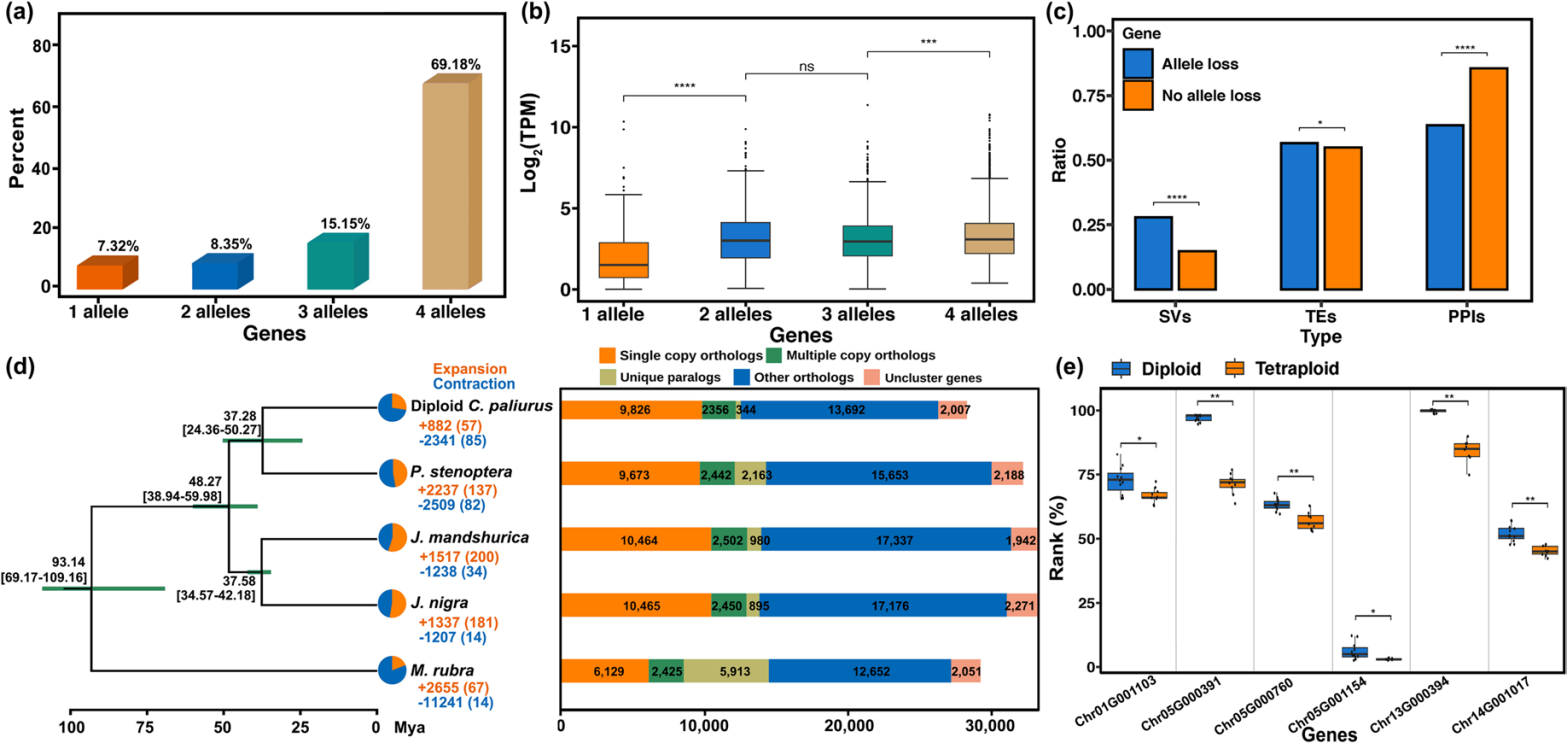
Diploid and autotetraploid *C. paliurus* gene family evolution. **a** Proportions of genes with 1–4 alleles in the autotetraploid genome. **b** Relationship between the number of alleles per gene and expression (TPM, transcripts per million). (Wilcox test, *p* value ≤ 0.001: ***;* p* value ≥ 0.05: ns). **c** Comparison of the associations with structural variations (SVs), transposable elements (TEs), and protein‒protein interactions (PPIs) between the genes with allele loss and no allele loss. **d** Phylogenetic tree for diploid *C. paliurus* and four outgroup species. The estimated divergence time (million years ago, Mya) is specified at each node, and green bars indicate the 95% CI (each center is defined as the mean value). Gene family expansion and contraction are indicated in orange and blue, respectively, in the pie charts, and the corresponding numbers are shown using the same colors. Genes of diploid *C. paliurus* and other reference genomes were classified into five classes. The absolute numbers of genes are shown in the bars. **e** The expression of six genes encoding sulfotransferase and 1,3-beta-D-glucan synthase was significantly upregulated in autotetraploids compared to diploids. (Fisher’s exact test, *p* value ≤ 0.0001: ****)

In autopolyploid genomes, homologous genes at the same locus on homologous chromosomes are defined as alleles [28]. Using two strategies to separate genes and alleles (see “Methods”), we annotated 29,817 genes containing 103,217 alleles with an average of 3.46 alleles per gene. The annotated genes comprised 20,626 (69.18%) genes with four alleles, 9191 genes with fewer than four alleles (allele loss) including 4516 (15.15%) genes with three alleles, 2490 (8.35%) genes with two alleles, and 2185 (7.32%) genes with one allele. These genes were significantly enriched in the trehalose biosynthetic process, the fatty acid biosynthetic process, and recognition of pollen, respectively (Fig. 4a and Additional file 2: Table S8). Genes with a higher number of alleles showed a significantly higher expression level than those with fewer alleles (Wilcox test, *p* value ≤ 0.001) (Fig. 4b). We extracted 4222 genes with four alleles that were all expressed in leaves (transcripts per million > 1.0) and observed no significant global allelic chromosome dominance (Additional file 1: Fig. S12). Similar findings were reported in *Saccharum spontaneum* [10] and cultivated alfalfa [11].

Compared with the genes with four alleles, the genes with three, two, or one allele were not randomly distributed in the genome. Among the 9191 genes with allele loss, the proportion of genes (26.15%) within or near SVs (located within 2000 bp of the SVs) was much higher than that of the genes with no allele loss (17.89%) (Fisher’s exact test,* p* value = 0; Fig. 4c, Additional file 2: Table S9). Several studies have found tetraploids with allele loss can involve accelerated rates of long deletions and translocations [29–32]. Among the total number of genes within translocations (939), inversions (1546), and duplications (992), the number of genes exhibiting allele loss in translocations (533, 56.76%) was significantly higher than that in inversions (413, 26.71%) and duplications (443, 44.66%) (Fisher’s exact test, *p* value < 0.01; Additional file 2: Table S10); but no significant correlation was observed between the gene exhibiting allele loss and the large deletion. Moreover, 56.37% of the genes with allele loss overlapped with TEs, compared with 54.92% of the genes with no allele loss (Fisher’s exact test, *p* value = 0.02; Fig. 4c, Additional file 2: Table S9). Interestingly, 85.43% of genes with no allele loss were identified as protein‒protein interaction (PPI) genes, whereas only 63.70% of the genes with allele loss were identified as PPI genes (Fisher’s exact test,* p* value = 0; Fig. 4c, Additional file 2: Table S9), indicating that genes with more connections were less prone to be lost.

### A disproportionate increase in gene number in autotetraploid *C. paliurus*

Duplicated genes play critical roles in phenotypic diversification [33] and adaptation [34]. Therefore, we conducted an expansion and contraction analysis of the gene families of *C. paliurus*. As CAFE [35] is not recommended for use in species that have experienced a recent WGD, we first conducted the analysis for diploid *C. paliurus.* We clustered the annotated genes into 32,276 gene families for diploid *C. paliurus* and four outgroup species (*P. stenoptera*, *J. mandshurica*, *J. nigra*, and *Morella rubra*) and obtained 22,438 gene families that contained less than 100 copies in the five species. To construct a phylogenetic tree as required by CAFE, we selected 461 shared single-copy genes with lengths between 500 and 1000 bp, separated by a distance of more than 20 kb, and with less than 20% missing matches among the five species. The phylogenetic tree showed that diploid *C. paliurus* and *P. stenoptera* formed a clade that was sister to a clade comprising *J. mandshurica* and *J. nigra* (Fig. 4d).

We detected 882 expanded families with a total of 2528 genes in diploid *C. paliurus* (Fig. 4d), and 1113 of the 2528 genes had four or more alleles in autotetraploid *C. paliurus*. Among these 1113 genes, the expression levels of 102 genes were significantly upregulated compared to those of the diploid based on the RNA-seq analysis of leaves (Additional file 5: Table S11). GO enrichment analysis of the 102 genes suggested significant enrichment (6 of the 102 genes) in sulfotransferase and 1,3-beta-D-glucan synthase activity (Fig. 4e and Additional file 1: Fig. S13), and two of the six genes were under positive selection in autotetraploids (Additional file 1: Fig. S14). Of the 102 genes, in addition to those two genes, seven genes related to protein phosphorylation and oxidation‒reduction processes were under positive selection (Additional file 1: Fig. S14).

Cold-regulated (*COR*) genes in the C-repeat binding factor (CBF) pathway are critical for cold acclimation and preferentially retained after WGD in higher plants [36, 37]. Using the genome of *C. paliurus*, we searched for homologs of previously reported *COR* genes in *Arabidopsis thaliana* [36, 38], and found 57 candidate *COR* genes in both diploid and autotetraploid *C. paliurus*. Forty-one of 57 *COR* genes had four or more alleles in autotetraploid *C. paliurus* (Additional file 2: Table S12); the expression of two of these genes was significantly upregulated, and three were under positive selection (Additional file 1: Fig. S15).

### Population genomic analysis of autotetraploid and diploid *C. paliurus*

**Fig. 5:**
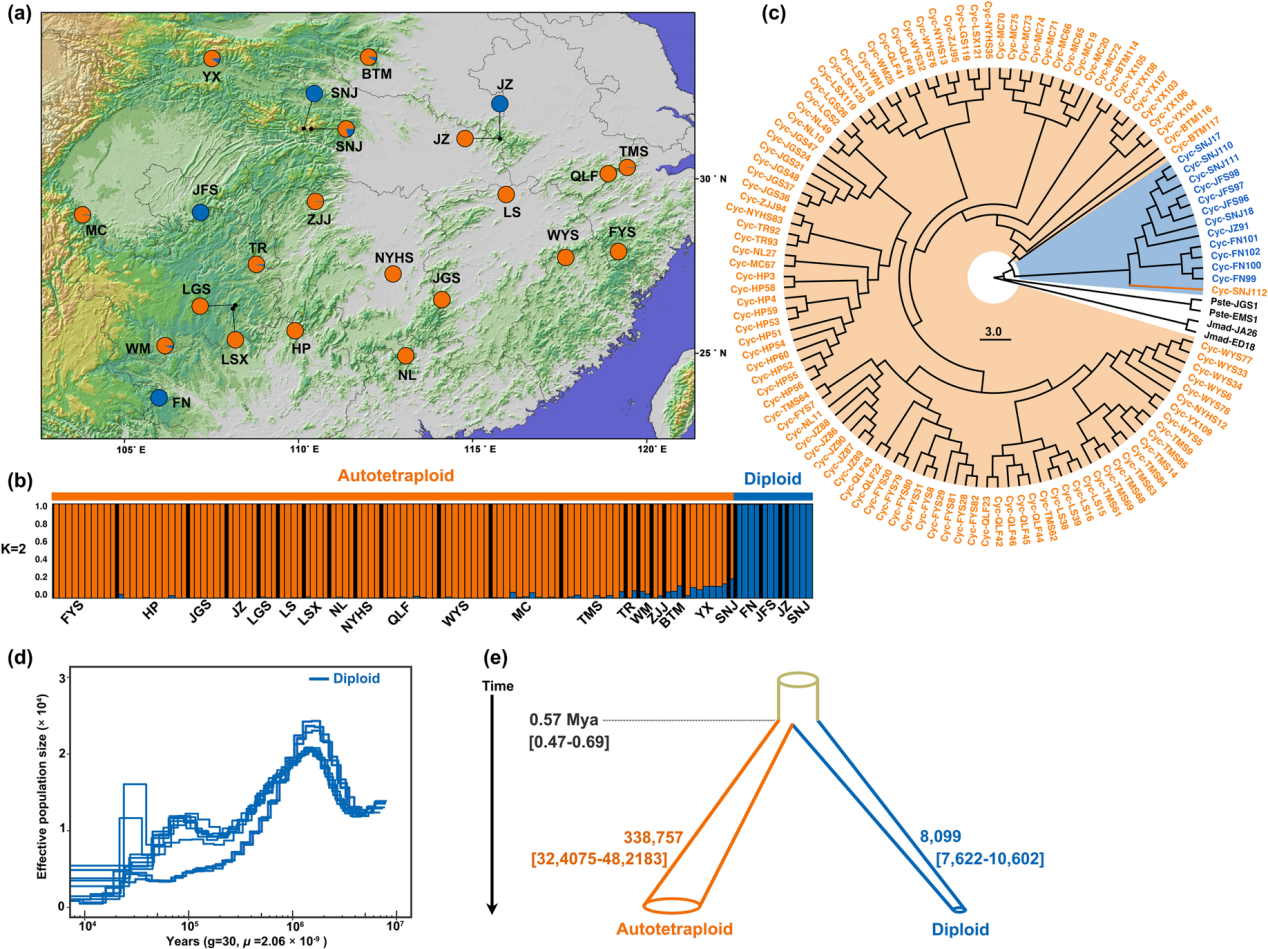
Population clustering and demographic history of *C. paliurus*. **a** Geographic locations of 118 *C. paliurus* samples from 21 populations. **b** Results of mixed-ploidy STRUCTURE analysis of 118 *C. paliurus* samples at *K* = 2. **c** The maximum likelihood phylogeny tree of 118 individuals and four outgroups with 7013 nuclear single-copy genes. A total of 105 autotetraploids clustered into one clade, and 12 diploids and one autotetraploid clustered into the other clade. Bootstrap support values are labeled on each branch. **d** Changes in effective population size over time for 12 diploid samples estimated with PSMC. **e** The divergence times and effective population sizes for autotetraploids and diploids were estimated using *fastsimcoal*2. The 95% parametric bootstrap CIs are specified in square brackets

To explore the origin of autotetraploid *C. paliurus*, we generated a comprehensive range-wide population genomic dataset of 118 individuals (Additional file 2: Table S13). We identified 12 diploids and 106 autotetraploids (Additional file 1: Fig. S16 and Additional file 2: Table S14). The nucleotide diversity (*π*) of autotetraploids (0.032 ± 0.01 per site) was much higher than that of diploids (0.015 ± 0.006 per site). The results of the mixed-ploidy STRUCTURE analysis using 2849 independent and neutral SNPs or 14,365 independent and synonymous SNPs for the 118 individuals indicated that *k* = 2 was the optimal number of groups when using the parsimony method of Wang [39] (Fig. 5a, b and Additional file 1: Fig. S17). At *k* = 2, the 106 autotetraploids formed one group and the 12 diploids formed the other group. To gain further insights into the structure of autotetraploids, we conducted additional analyses using STRUCTURE and ENTROPY with 3191 neutral and independent SNPs or 25,577 independent and synonymous SNPs for the 106 autotetraploids. However, the results of these analyses still suggested that *k* = 1 was the optimal number of groups (Additional file 1: Fig. S18 and S19). The maximum likelihood (ML) phylogenetic tree for the 118 individuals showed that 105 autotetraploids were resolved into one clade, and 12 diploids and one autotetraploid individual were grouped into a separate clade (Fig. 5c). The single ‘outlier’ autotetraploid individual was sympatric with four diploid individuals and had an admixture proportion of 0.2 in the mixed-ploidy STRUCTURE analysis.

We used pairwise sequentially Markovian coalescent (PSMC) modeling to infer the changes in effective population size (*N*_e_) over time for the 12 diploid individuals. The *N*_e_ value increased to the maximum (*N*_e_ ≈ 2.43 × 10^4^) between 2 and 1.5 Mya and thereafter declined rapidly, and the smallest population size (*N*_e_ ≈ 0.31 × 10^4^) was observed to be between 25 and 10 Kya (Fig. 5d).

When using *fastsimcoal*2 to simulate population divergence, the divergence time between diploids and autotetraploids was ~ 0.57 Mya (95% CI 0.47–0.69 Mya), and *N*_e_ was 0.81 × 10^4^ (95% CI 0.76–1.10 × 10^4^) for diploids and 3.39 × 10^5^ (95% CI 3.24–4.82 × 10^5^) for autotetraploids (Fig. 5e). To investigate the pattern of inheritance in the autotetraploid *C. paliurus*, we conducted coalescent simulations to generate the site frequency spectrum (SFS). Our analysis revealed that at most 30% of the tetraploid genome have undergone disomic inheritance (see “Methods”; Additional file 1: Fig. S20 and Additional file 2: Table S15), indicating the process of rediploidization, in which tetraploid genomes evolve towards disomic inheritance, is gradual and ongoing [40–42].

To further explore how natural selection shapes genetic variation in autotetraploid and diploid *C. paliurus*, genome-wide detection of positive selection with the DCMS (Decorrelated Composite of Multiple Signals) method was performed. We identified 1405 genes in autotetraploids and 1257 genes in diploids that displayed a significant DCMS score (*p* value < 0.05; Additional file 1: Fig. S21). Our GO enrichment analysis of the 1405 genes in autotetraploids revealed significant enrichment (eight genes) for the term “response to biotic stimulus” (Additional file 1: Fig. S22 and Additional file 2: Table S16). Four of these genes were found to be homologous to genes in *A. thaliana* (*MLP43*, *MLP328, MLP31*, and *AGD3*) that encode proteins responsible for responding to various stresses like abscisic acid, cis-cinnamic acid, salicylic acid, and auxin [43–46]. GO enrichment analysis for the 1257 genes in diploids showed no significant enrichment for any term.

Given that even conserved meiotic processes can show evolutionary shifts in response to selective pressures in autotetraploid *A. arenosa* [47–49], we examined 62 orthologs of meiosis-related genes from *A. thaliana* in *C. paliurus* (Additional file 2: Table S17). Our analysis revealed that eight of these genes were under positive selection (Additional file 1: Fig. S22 and Additional file 2: Table S18). Interestingly, two of the eight genes (*ZYP1a* and *ASY3*) that were under positive selection in *C. paliurus* have also been found to be under positive selection in *A. arenosa*. This suggests that the phenomenon of conserved meiotic processes exhibiting nimble evolutionary shifts in autotetraploids may have broader significance that warrants further investigation.

## Discussion

### Origin and evolution of autotetraploid *C. paliurus*

In this study, we reported an allele-aware chromosome-level autotetraploid genome and a chimeric well-organized diploid genome for *C. paliurus*. Many previous studies have reported that WGDs are far more prevalent in the evolutionary history of flowering plants [50–54]; consistent with those reports, we discovered an independent, recent WGD in autotetraploid *C. paliurus* (Additional file 1: Fig. S6), in addition to diploid *C. paliurus* and other Juglandaceae species that have experienced two rounds of ancient WGD events [25]. We observed similar divergence between diploid and autotetraploid genomes and among the monoploid genomes (Fig. 3 and Additional file 1: Fig. S7 – S10), indicating that autotetraploid *C. paliurus* was recently formed, a conclusion supported by the divergence time estimated with *fastsimcoal*2 (Fig. 5d).

Studies of polyploid taxa have documented multiple origins of polyploidy in at least 40 species, including both autopolyploids and allopolyploids [55–61]. Autotetraploid lineages often arise from more than a single individual in many species, so research on single-origin autopolyploid species is rarely reported (but see *A. arenosa* [62] and sweet potato [7]). The observation that autotetraploid individuals comprised a single group, as illustrated by the STRUCTURE analysis and maximum likelihood phylogeny tree, indicated that extant autotetraploids of *C. paliurus* may have originated and radiated from a single ancestral population (Fig. 5 and Additional file 1: Fig. S17 – S19).

### Genome structural and functional reorganization of autotetraploid *C. paliurus*

Autopolyploids are generally thought to experience less genome restructuring than allopolyploids. However, it has been reported that some autopolyploids undergo significant chromosome length decrease [63, 64], relocation of chromosomal segments, asymmetrical relocation, and loss of rDNA loci [65]. These results indicate that autopolyploids can undergo substantial genome reorganization compared to their diploid relatives. In our study, we identified 9191 genes (30.82%) with allele loss, of which 69.08% were also found in sugarcane [66], and 47% were found in potatoes [12]. Our study revealed that genes exhibiting allele loss are significantly associated with structural variations and transposon mobilization. This biased pattern was similar to the fractionation in allopolyploids, which is sometimes linked to chromosome breaks, large-scale rearrangements, centromere loss, and transposon mobilization [31]. The underlying mechanisms of this phenomenon remain not fully understood; however, it is believed that long-term genome instability and a biased pattern of allele loss play a role in gradually restoring diploid-like behavior to autopolyploid genomes over time. Furthermore, the genes coding for PPI products tend to retain four alleles, indicating that dosage balance constraints may be a major factor affecting the loss or retention of duplicate genes [67–69]. Albalat and Canestro [70] concluded that genes with functions in the GO categories of “transcriptional regulation,” “signal transduction,” or “protein‒protein interacting complexes” are unlikely to be lost after WGD.

WGD events have often occurred during periods of severe global environmental change, including global cooling, darkness, acid rain, and wildfires [37, 71, 72]. Therefore, polyploidy may enhance the tolerance of individuals to environmental stress. Among the 1113 genes with significant post-WGD allelic duplications, we observed that the expression levels of 102 genes were significantly upregulated in autotetraploid *C. paliurus*. This gene set was notably enriched (six out of 102 genes) in sulfotransferase and 1,3-beta-D-glucan synthase activity, both of which play essential roles in plant defense, stress response, signaling, and developmental regulation [73]. Moreover, we detected 41 *COR* genes with four or more alleles in autotetraploid *C. paliurus* (Additional file 2: Table S12)*.* Furthermore, we identified 41 *COR* genes with four or more alleles in autotetraploid *C. paliurus* (Additional file 2: Table S12). The divergence time between autotetraploid and diploid *C. paliurus* was approximately 0.57 Mya, which coincided with the period of coolest temperatures in the Quaternary [74]. In comparison to diploids, which experienced severe population bottlenecks during that period (Fig. 5c), these significantly expanded genes may have contributed to enhancing autotetraploid adaptability to harsh environments.

## Conclusions

Our study found that autopolyploid *C. paliurus* had a single origin and recent divergence from diploids, but there was very little interploidy gene flow observed. Contrary to our initial expectations, autopolyploid *C. paliurus* underwent a certain degree of genome restructuring after WGD, as evidenced by biased allelic loss, a disproportionate increase in gene number, and enhanced expression of genes encoding important enzymes. These adaptations have allowed them to thrive and persist over time.

## Methods

### Sampling and sequencing

Fresh leaves from two individuals of *C. paliurus* were collected from the Institute of Botany, Chinese Academy of Sciences, Beijing (39° 58′ 58.8″ N, 116° 12′ 32.40″ E) and from Shennongjia National Park, Hubei Province, China (31° 27′ 2.79″ N, 110° 8′ 57.36″ E) for de novo genome assembly. First, we used flow cytometry to evaluate the ploidy of the two individuals with *Juglans regia* as the standard. The peaks of the individual from Shennongjia National Park and *J. regia* were in a similar position, while the peak position of the individual from the Institute of Botany was double that of *J. regia*, indicating that the individual from Shennongjia National Park was diploid and the one from the Institute of Botany was tetraploid. Next, root apical meristems of 1-year-old seedlings were excised and treated with nitrous oxide to prepare chromosome slides. We performed FISH analysis using 5S rDNA and 18S rDNA sequences. A total of 64 chromosomes were counted for the tetraploid by FISH analysis. Third, we used nQuire and GenomeScope 2.0 with ~ 60 Gb Illumina paired-end short reads of 350 bp to determine whether the tetraploid individual was an autotetraploid. nQuire is a statistical approach for ploidy estimation based on the distribution of base frequencies at variable sites using a Gaussian mixture model. GenomeScope 2.0 applies combinatorial theory to establish a detailed mathematical model of the distribution of *K*-mer frequencies in heterozygous and polyploid genomes.

We used paired-end libraries with an insert size of 350 bp to generate ~ 57 Gb of short reads on an Illumina NovaSeq 6000 platform and 15 kb CCS libraries to obtain ~ 73 Gb of CCS long reads on a PacBio Sequel II platform for the same individual. For the diploid individual, we sequenced ~ 27 Gb of Illumina short reads and ~ 23 Gb of CCS long reads using the same platforms as for the autotetraploid sample. Sequencing was performed by NovoGene (Beijing, China). We constructed a Hi-C fragment library and obtained ~ 156 and ~ 92 Gb of clean Hi-C reads for autotetraploid and diploid *C. paliurus*, respectively.

### Genome assembly and annotation

The autotetraploid *C. paliurus* genome was assembled as follows: First, we utilized approximately 73 Gb of PacBio CCS long reads to perform the initial contig-level assembly with hifiasm v. 0.13-r308 [75]. Four different sets of parameters “-l 2 -n 4,” “-l 2 -n 3,” “-l 0 -n 4,” and “-l 0 -n 3” were employed. After a comprehensive comparison, the result of the parameter “-l 2 -n 4” proved to be optimal and has been chosen for subsequent scaffold assembly into chromosome-level assemblies using Hi-C data. The comparison was based on various key factors, including a lower number of contigs, the longer contig N50 lengths, and a genome size that closely aligned with the expected size based on the genome survey. Next, we attempted to assign the contigs to different haplotype groups with the assistance of Hi-C data. A total of 156 Gb Hi-C reads were mapped to the contig-level assembly using BWA v. 0.7.10 [76] with default parameters. After performing pruning on the mapping results using HiC-Pro v. 2.8.1 [77], only the validated Hi-C read pairs were kept for the correction of contigs. The contigs that were fully covered by Hi-C reads were retained, while any contigs with not fully covered by Hi-C reads were partitioned into two or more smaller segments. Then, we utilized LACHESIS [78] to cluster, order, and orient the contigs based on chromatin interaction signals, with the parameters set to “cluster_min_re_sites = 84, cluster_max_link_density = 2; order_min_n_res_in_trunk = 81; order_min_n_res_in_shreds = 81.” Finally, the resulting assembly was manually corrected according to the visualization of chromatin contact patterns, and we generated a pseudochromosome genome that included 64 chromosomes.

The details of the diploid genome assembly are provided in Additional file 6: Note 1 [79–109]. In addition to the two genome sequences, we used ~ 77 Gb of Hi-C reads to improve the genome assembly of *P. stenoptera* v. 2.0 [110] to the chromosome level (assembly v. 3.0) and downloaded chromosome-level reference genomes for *J. mandshurica* [111], *J. nigra* [110], and *M. rubra* [112] as outgroup species to construct a phylogenetic tree for subsequent analysis.

Fresh leaves and fruits of four autotetraploid and four diploid individuals were collected for RNA sequencing and genome annotation. To annotate the genomes of autotetraploid and diploid *C. paliurus*, a combination of homology-based inference, ab initio prediction, and transcripts from RNA sequencing was used (for details, see Additional file 6: Note 2).

### Whole-genome resequencing and variant calling

We collected leaves of 118 adult individuals throughout the distribution range of *C. paliurus* in China from 23° N to 33° N and 103° E to 119° E (Additional file 2: Table S13). Whole-genome resequencing was performed using paired-end libraries with an insert size of 350 bp on an Illumina NovaSeq 6000 platform with an average depth of 60 × per individual for 106 autotetraploid individuals, along with an average depth of 90 × per individual for 12 diploid individuals.

The paired-end reads for the 118 individuals were trimmed to remove the adaptors and low-quality bases using Trimmomatic v. 0.32 [113]. Trimmed reads from each individual were mapped to the reference genome of diploid *C. paliurus* using BWA-MEM v. 0.7.12 [76] with the default parameters. We used nQuire to determine the ploidy of the 118 individuals with the “denoise” subcommand to reduce the high baseline of noise. Then, we called variants for the 118 BAM files using “Haplotyper” implemented in the Sentieon DNASeq v. 202112.01 package [114]. For each autotetraploid individual, “Haplotyper” was run in parallel with the parameters set to “–ploidy 4.” We combined the single-sample GVCF output from “Haplotyper” to create a multisample SNP dataset using “GVCFtyper” in DNASeq and filtered the SNPs following the filtering strategy of Xu et al. [115], except that tri-allelic or tetra-allelic SNP sites for the autotetraploid individuals were retained. Linkage disequilibrium (LD) for diploid and autotetraploid *C. paliurus* was calculated using PopLDDecay v. 3.40 [116]. To obtain neutral and independent SNPs, those located in coding sequences (CDS) and its 20 kb extension region were discarded and further thinned using a distance filter of 20 kb based on LD results (Additional file 1: Fig. S23). To reduce false-positive effects caused by sequencing error, we filtered SNPs with minor allele frequencies (MAF) < 0.01. After filtering, we retained 2849 high-quality, neutral, and independent SNPs for the subsequent cluster analysis (Additional file 2: Table S19).

### Whole-genome duplication analysis

The conserved paralogs of the protein sequences of diploid and autotetraploid *C. paliurus*, *P. stenoptera*, *J. mandshurica*, and *J. nigra* were obtained with BLASTP with a typical cutoff *E*-value ≤ 1 × 10^−10^. MCScanX [117] was applied to find the syntenic blocks of the four species with the default parameters from the top ten self-BLASTP hits. Pairwise *K*_s_ values of syntenic paralogous genes were estimated with the script “add_ka_and_ks_to_collinearity.pl” in MCScanX. For autotetraploid *C. paliurus*, we estimated the *K*_s_ values of all 64 chromosomes to identify the WGD for autotetraploid *C. paliurus*.

### Genome variation between autotetraploid and diploid *C. paliurus*

For each possible pair within the four monoploid genomes of the autotetraploid, the two monoploid genomes were mapped against each other using the “nucmer” function implemented in the MUMmer4 package [118] with the parameters “-c 500 -b 500 -l 100.” We filtered the alignments using the “delta-filter” with “-i 90 -l 100” parameters and removed repetitive sequences. The best hits were retained. To obtain SNPs and SVs of any two of the four monoploid genomes, we applied the “show-snps” function in MUMmer4 to identify SNPs with the parameters “–ClrHIT” and applied SyRI [119] to identify inversions, translocations, and duplications. The “show-aligns” function in MUMmer4 was used to obtain syntenic sequences of any two monoploid genomes. We then computed* D*_XY_ for syntenic regions using a Python script [120]. *D*_XY_ was computed in 50 kb stepping windows if the syntenic region was ≥ 50 kb and for the whole region if the syntenic region was < 50 kb. Syntenic blocks between any two monoploid genomes were identified using MCScanX based on the results of an all-to-all BLASTP search. The *K*_s_ values of syntenic genes were estimated for any two monoploid genomes using the script “add_ka_and_ks_to_collinearity.pl” in MCScanX.

Comparisons between the diploid and autotetraploid genomes were conducted using the same software and parameters as described above. Each of the four monoploid genomes of the autotetraploid was mapped against the diploid genome to identify SNPs and SVs. We further inferred the same SVs among the four monoploid genomes of the autotetraploid. Two or more SVs were defined as identical if (1) there was at least 50% overlap in the reference diploid genome, (2) the chromosomes in the four monoploid genomes were allelic chromosomes, and (3) two or more SVs were of the same type. We classified each SV into one of 15 classes by inferring the same SVs. The *D*_XY_ and *K*_s_ values between the diploid and any one of the four monoploid genomes were calculated using the same scripts described above.

### Identification of alleles

AlleleFinder (https://github.com/sc-zhang/AlleleFinder), a synteny and coordinate-based pipeline, was applied to identify alleles in the autotetraploid *C. paliurus* genome. First, interhaplotype syntenic blocks were detected by MCScanX with the default parameters. The CDSs of the autotetraploid were mapped to the diploid *C. paliurus* genome using GMAP v. 2020–06–30 [121]; those with at least 50% overlap of coordinates were considered to be potential alleles. Next, syntenic blocks and potential alleles were integrated based on sequence similarities. Finally, the anchor genes in diploid *C. paliurus* and corresponding alleles in autotetraploid *C. paliurus* were identified and integrated into a table.

### Allele loss analysis based on SVs, TEs, and PPIs

Based on the SVs between the diploid and autotetraploid genomes, we classified genes with allele loss and no allele loss into three categories: (1) genes within one or multiple types of SVs, with “multiple types” referring to different SVs in the four monoploid genomes of autotetraploids at the same positions, (2) genes within the region of SVs around 2000 bp, and (3) genes not close to (> 2000 bp) SVs (Additional file 2: Table S9). Based on the results of TE annotation, we classified the genes with allele loss and no allele loss into two categories: (1) genes overlapping with one or multiple types of TEs and (2) genes not overlapping with one or multiple types of TEs (Additional file 2: Table S9). Furthermore, we used the STRING [122] database v.11.0 (https://cn.string-db.org/) to identify PPI genes among the genes with and without allele loss. Reciprocal best hits (RBH) between diploid *C. paliurus* and *J. regia* for protein sequences from the STRING database were used to obtain PPI genes (Additional file 2: Table S9). Fisher’s exact test was used to compare the differences in ratios between genes with and without allele loss.

### Gene family expansion analyses

To identify gene families, the protein sequences of *C. paliurus* (diploid and autotetraploid) and four outgroup species (*P. stenoptera*, *J. mandshurica*, *J. nigra*, and *M. rubra*) were used with OrthoFinder v. 2.5.2 using default parameters. For species phylogeny inference required in the CAFE analysis, we obtained a set of single-copy orthologs from the OrthoFinder results. MAFFT v. 7.273 was used to align the single-copy orthologous genes between 500 and 1000 bp in length and at 20 kb intervals. Sequences with more than 20% missing data were excluded. The aligned protein sequences of the single-copy orthologs were converted to a CDS alignment with PAL2NAL. Next, ML trees were inferred from the 461 single-copy orthologous genes shared by all species using RAxML v. 8.0.26 (with 100 bootstrap replicates) with the GTR + gamma substitution model. ASTRAL v. 4.10.10 [123] was used to construct a coalescent tree from the gene trees, and MCMCTree in the PAML package [124] was used to estimate the species divergence time. One fossil date was used as a minimum-age calibration point, i.e., 35–43 Mya, as the stem age of the ancestor of *J. mandshurica* and *J. nigra*.

CAFE v. 4.2.1 was applied to estimate gene family size across an ultrametric phylogenetic tree. Given that large variance in gene copy numbers of gene families across species may result in noninformative parameter calculations, we filtered out all gene families with more than 100 copies. We used the parameter *λ* (lambda value = 0.00291075) to describe the rate of change as the probability that a gene family either expands or contracts per gene per million years after simulating an error model that takes into account errors in genome assembly and gene annotation for all analyzed genomes [35].

### Identification and analysis of *COR* genes in *C. paliurus*

Fifty-six *COR* protein sequences of *Arabidopsis* from previous reports [36, 125] were employed in searches against diploid and autotetraploid *C. paliurus*. Homologous *COR* genes in *C. paliurus* were identified using BLASTP (*E*-value < 1 × 10^−10^, identity ≥ 70%, coverage ≥ 40) in accordance with a previous report [126]. Each *COR* gene and corresponding homologs in the diploid *C. paliurus* genomes was retained using a Python script. Finally, the allele numbers of each *COR* gene in the autotetraploid *C. paliurus* genome was obtained based on the result of allele identification.

### Population genomic analysis of diploid and autotetraploid individuals

Twelve diploid individuals and four randomly selected autotetraploid individuals (replicated 20 times) were used to calculate *π* using ANGSD [127] with BAM files in a 50 kb sliding window and 20 kb steps. We used 2849 high-quality, neutral, and independent SNPs and 14,365 independent and synonymous SNPs to perform a STRUCTURE analysis for 118 individuals. We conducted additional analyses using STRUCTURE and ENTROPY with 3191 neutral and independent SNPs and 25,577 independent and synonymous SNPs for the 106 autotetraploids.

We obtained 8047 genes for 118 *C. paliurus* individuals, two *P. stenoptera* individuals, and two *J. mandshurica* individuals using OrthoFinder v. 2.5.2 [128]. MAFFT v. 7.273 [129] was used to align the 7013 genes with less than 20% missing data. The aligned protein sequences of these genes were converted to a CDS alignment with PAL2NAL [130]. Combining the 7013 genes of the 122 individuals, we used RAxML v. 8.0.26 [131] to infer an ML tree under the GTR + gamma substitution model with 1000 bootstrap replicates.

We used PSMC v. 0.6.5-r67 [132] to infer the change in *N*_e_ over time for 12 diploids that were mapped to the diploid reference genome. The PSMC parameters were set with quality adjusted to 50, a minimum mapping quality of 20, a minimum depth of one-third average depth of genome coverage, and a maximum depth of twofold average depth of genome coverage. The analysis commands included the options “-N25” for the number of cycles of the algorithm, “-t15” as the upper limit for the most recent common ancestor, “-r5” for the initial *h*/*q* value, and “-p 4 + 25*2 + 4 + 6” atomic intervals. The reconstructed population history was plotted with the substitution rate “-u 2.06e-9” and a generation time of 30 years [27].

We estimated the divergence time for eight diploid samples and 19 autotetraploid samples from 19 populations of *C. paliurus* using *fastsimcoal*2 [133]. We filtered the SNPs and randomly subsampled two alleles per site for the autotetraploids. Two-dimensional joint site frequency spectra (2D-SFS) were constructed with easySFS (https://github.com/isaacovercast/easySFS). We then performed 100,000 coalescent simulations and computed log-likelihoods based on simulated and observed 2D-SFS matrices. Global ML estimates for the best model were obtained from 100 independent runs, with 50 expectation conditional maximization algorithm cycles. A parametric bootstrapping approach was used to construct 95% CIs with 100 independent runs for each bootstrap.

### Inheritance of autotetraploid *C. paliurus*

There are two extreme models for diploid gametes produced by tetraploid plants, i.e., disomic in allotetraploids and tetrasomic in autotetraploids [134–136]. We investigated fully disomic and tetrasomic inheritance models in autotetraploid *C. paliurus* by comparing the SFS of our sequence data with the simulated datasets. We employed the software ms [137] to simulate data with different time settings since evolution of disomic inheritance (td; in units of 4N generations) from td = 1 to 0.2, with steps of 0.2, and a fully tetrasomic inheritance, following the methodology outlined in Hollister et al. [138] (for details, see Additional file 6: Note 3). Furthermore, we conducted simulations at different proportions of genome (at intervals of 10%, ranging from 10 to 90%) exhibiting disomic inheritance, which occurred under different time settings. Using these simulations, we generated the expected neutral site frequency spectrum (SFS). We then selected 12 tetraploid individuals with a total of 30,124,148 high-quality SNPs to create the observed SFS. We used the one-sided two-sample Kolmogorov–Smirnov test, which is a more powerful tool for assessing the goodness of fit of a theoretical distribution to observed data [139]. Our statistical analysis showed significant differences between our observed data and simulated data for both disomic and tetrasomic inheritance (Additional file 1: Fig S20 and Additional file 2: Table S15).

### Detection of positive selection

To detect regions under selection across the genome of autotetraploid *C. paliurus*, we scanned the genome for multiple patterns of molecular variation: (1) locally elevated levels of genetic differentiation and (2) distortions in the allele frequency spectrum. We applied a combination of statistics (*F*_ST_, Tajima’s *D* [140], composite likelihood ratio [CLR; 141] and Fay-Wu’s *H*) in nonoverlapping 25 kb windows, with *J. mandshurica* as an outgroup and a minimum of 10 SNPs per window. Tajima’s *D* and *F*_ST_ were calculated using VCFtools v. 0.1.17 [142]; the CLR statistic was calculated using SWEEPFINDER2 [143]; and Fay-Wu’s *H* was calculated using VariScan v. 2.0 [144].

In our analysis of autotetraploid data, we used random subsampling of two alleles per site to compute four statistics (*F*_ST_, Tajima’s *D*, CLR, and Fay-Wu’s *H*.) for each of the five replicate datasets. We conducted rank sum tests to assess whether there were any significant differences among the results obtained from five replicate datasets. The results of the tests indicated that there were no significant differences observed among them (Additional file 1: Fig. S24). To facilitate the integration of the results of the four statistics, we computed ΔTajima’s *D* as Tajima’s *D*_*diploid*_—Tajima’s *D*_*autotetraploid*_ and Δ*CLR* as *CLR*_*autotetraploid*_—*CLR*_*diploid*_ for each window.

All four statistics were combined using the DCMS method, a composite method for the detection of selection that combines molecular signals of different tests and considers potential correlations among the different tests. DCMS is expected to increase resolution and reduce the proportion of false positives [145]. For each statistic, we tested whether its distribution fit the normal distribution using the R package Cmplot v. 4.0.0 [146]. If not, we performed a two-step normalization approach [147]. First, the variable is transformed into a percentile rank, which results in uniformly distributed probabilities; second, the inverse-normal transformation is applied on the percentile ranks to form a variable consisting of normally distributed *Z* scores. Normalized scores for each statistic were *Z* transformed, and a *p* value was derived from this transformation. The correlation of the *p* value of each statistic was calculated and used to calculate their weight factors. Finally, the DCMS was estimated for each window, and a *p* value was derived for each 25 kb window following the method described above. Regions under putative positive selection were defined as the windows with a *p* value < 0.05. We also conducted the analyses with a 10 kb window and found a total of 1560 genes under positive selection for diploids and 1683 genes for tetraploids. Considering there still exhibited linkage equilibrium in the 10 kb, we think maybe it is better to choose a 25 kb window.

Finally, 71 meiosis-related genes of *Arabidopsis* [49] were downloaded from The Arabidopsis Information Resource database (https://www.arabidopsis.org/) and 62 homologous meiosis-related genes in *C. paliurus* were identified (Additional file 2: Table S17). After analyzing the 62 genes in *C. paliurus*, we were able to identify specific genes related to meiosis that exhibited positive selection.

### RNA-seq analysis

Fresh leaves of three autotetraploid *C. paliurus* individuals were used for RNA extraction and sequencing. Paired-end RNA-sequencing reads were trimmed with Trimmomatic v. 0.39 [113] with “SLIDINGWINDOW:4:15 LEADING:3 TRAILING:3 MINLEN:36” and then mapped to the autotetraploid *C. paliurus* genome using HISAT2 v. 2.2.1 [148] (Additional file 2: Table S20). The number of fragments mapped to genes was quantified with HTSeq v. 2.0.0 [149]. The fragment counts were normalized as log_2_-transformed transcripts per million (TPM). To explore the impact on gene expression of the number of alleles, the expression of genes with different alleles was compared (* indicates statistical significance: Wilcox test), where the expression of each gene was the average log_2_-transformed TPM for all alleles.

To further examine the transcriptomic differences between diploid and autotetraploid populations of *C. paliurus*, we applied RNA-seq to two batches (3 + 8 replicates from leaves) of diploid samples and two batches (3 + 6 replicates from leaves) of autotetraploid samples (for sample details, see Additional file 2: Table S21). We obtained the count matrix of transcripts for each sample by HTSeq count using the diploid transcriptome as a reference. To minimize potential batch effects, differentially expressed genes (DEGs) between diploids and autotetraploids were detected based on a rank-based nonparametric DEG detection method. First, for the transcriptomic profiling of each replicate, genes were first rearranged according to their expression levels from high to low. Next, we gave a percentile rank to each gene from 1 to 100%, sorting them into 100 groups. Then, we constructed a ranking matrix using the internal gene ranks of each profile. Finally, we applied the Wilcoxon rank sum test to compare the gene rank differences between diploid and autotetraploid samples. Genes with a *p* value < 0.05 (corrected by the Benjamini‒Hochberg method) were regarded as significant DEGs.

### GO enrichment analysis

The GO terms in the biological process category were extracted for GO functional enrichment analysis of different gene lists in this study using the R package “clusterProfiler” v. 4.0.5 [150]. Specifically, we selected 13,214 diploid genes and 88,763 tetraploid genes, all annotated with biological processes, as background sets. We then performed gene enrichment analysis using the enrichGO function in clusterProfiler, setting a *p* value and *q* value cutoff of 0.05, and using the Benjamini–Hochberg (BH) correction method to control the false discovery rate. The significance of enrichment was assessed using Fisher’s exact test (*p*. adjust < 0.05). Finally, we visualized the results using the dotplot function.

### Supplementary Information

**Additional file 1:  Fig. S1.** Hi-C heatmaps. **Fig. S2 and S3.** Plots of mapping depth of Hifi reads. **Fig. S4.** Plots of LAI score. **Fig. S5.** Structural variations among the four chromosomes of autotetraploid genome. **Fig. S6.** Whole-genome duplication results of *C. paliurus*. **Fig. S7– S10.** *D*_XY_ and *K*_s_ results of *C. paliurus*. **Fig. S11** and **S13.** GO enrichment results. **Fig. S12.** Expression levels of genes with four alleles. **Fig. S14, S15** and **S22.** Several genes are under positive selection. **Fig. S16.** The ploidy estimation results of two *C. paliurus* samples. **Fig. S17.** The results of the mixed-ploidy STRUCTURE analysis. **Fig. S18** and **S19.** Plots depicting STRUCTURE and ENTROPY results for autotetraploid *C. paliurus*. **Fig. S20.** The SFS of simulated data and autotetraploid *C. paliurus*. **Fig. S21.** DCMS values of *C. paliurus.* **Fig. S23.** LD decay patterns of *C. paliurus*. **Fig. S24.** Statistical tests of five replicate datasets.

**Additional file 2: Table S1.** BUSCO and CEGMA assessments. **Table S2.** The coverage rates of Illumina reads and Hifi reads mapping to autotetraploid genome. **Table S3 and S6.** Summaries of repetitive elements for *C. paliurus*. **Table S4.** The length and gene count of autotetraploid genome. **Table S5 and S7.** Number of SNPs and SVs in autotetraploid genome. **Table S8.** Allele annotations of autotetraploid *C. paliurus*. **Table S9 and S10.** Summary numbers of genes with/without allele loss in SVs, TEs and PPIs. **Table S12.** *COR* genes with four or more alleles of autotetraploid *C. paliurus*. **Table S13.** Sampling and mapping details of 118 *C. paliurus* individuals. **Table S14.** Ploidy estimation for 118 individuals. **Table S15.** Inheritance of autotetraploid *C. paliurus*. **Table S16 and S18.** Information on several genes under positive selection. **Table S17.** The 62 meiosis-related genes in *C. paliurus*. **Table S19.** Number of remaining SNPs after filtering. **Table S20.** Mapping information for allele expression analysis. **Table S21.** Several samples were used for RNA-seq analysis.

**Additional file 3.** Instances copy gain and copy loss in autotetraploid *C. paliurus*.

**Additional file 4.** 104 breakpoints across the autotetraploid genome.

**Additional file 5:** **Table S11.** Details of 102 significantly upregulated genes in autotetraploid *C. paliurus*.

**Additional file 6:** **Note 1.** Genome assembly of diploid *C. paliurus* and *P. stenoptera*. **Note 2.** Genome annotation of *C. paliurus* and *P. stenoptera*. **Note 3. **Autotetraploid *C. paliurus* inheritance mode.

## Data Availability

The whole-genome resequencing data, FASTA files, and GFF files of the three genomes reported in this paper have been deposited in the NCBI database under BioProject number PRJNA356989 and deposited in the National Genomics Data Center under BioProject number PRJCA010540 (https://ngdc.cncb.ac.cn/bioproject/browse/PRJCA010540). Detailed alignment plots of HiFi reads to the autotetraploid genome are available on our website (https://cmb.bnu.edu.cn/Cyclocarya/index.php/download). The analyzed datasets and custom scripts used in this study are deposited in figshare (https://figshare.com/s/68348ea581f91a056fd3).

## Abbreviations

WGD: Whole-genome duplication
CCS: Circular consensus sequencing
Hi-C: High-throughput chromosome conformation capture
FISH: Fluorescence in situ hybridization
LTRs: Long terminal repeats
LAI: LTR Assembly Index
SNPs: Single-nucleotide polymorphisms
SVs: Structural variations
TEs: Transposable elements
*K*_s_: Synonymous substitution rate
*D*_XY_: Pairwise nucleotide difference
PPI: Protein‒protein interaction
COR: Cold-regulated
CBF: C-repeat binding factor
*π*: Nucleotide diversity
ML: Maximum likelihood
PSMC: Pairwise sequentially Markovian coalescent
*N*_e_: Effective population size
CIs: Confidence intervals
SFS: Site frequency spectrum
DCMS: Decorrelated composite of multiple signals
LD: Linkage disequilibrium
MAF: Minor allele frequencies
CDSs: Coding sequences
RBH: Reciprocal best hits
2D-SFS: Two-dimensional joint site frequency spectra
CLR: Composite likelihood ratio
TPM: Transcripts per million
DEGs: Differentially expressed genes
BH: Benjamini–Hochberg
