# Author Correction: Genomic insights into biased allele loss and increased gene numbers after genome duplication in autotetraploid *Cyclocarya paliurus*

**DOI:** 10.1186/s12915-023-01710-2

**Published:** 2023-09-29

**Authors:** Rui-Min Yu, Ning Zhang, Bo-Wen Zhang, Yu Liang, Xiao-Xu Pang, Lei Cao, Yi-Dan Chen, Wei-Ping Zhang, Yang Yang, Da-Yong Zhang, Er-Li Pang, Wei-Ning Bai

**Affiliations:** State Key Laboratory of Earth Surface Processes and Resource Ecology, and Ministry of Education Key Laboratory for Biodiversity Science and Ecological Engineering, College of Life Sciences, Beijing Normal University, Beijing, 100875 China

**Correction: BMC Biology 21** **,** **168 (2023)**

**https://doi.org/10.1186/s12915-023-01668-1**

The original article [1] mistakenly omitted co-first authorship for Ning Zhang which is acknowledged via this Correction article.
