# Supplementary material for: Genomic insights into biased allele loss and increased gene numbers after genome duplication in autotetraploid Cyclocarya paliurus
Source: BMC Biol. 2023 Aug 8;21:168. doi: 10.1186/s12915-023-01668-1 (PMC10408227; doi:10.1186/s12915-023-01668-1)
Supplement: Supplementary file 1 — Additional file 1: Fig. S1. Hi-C heatmaps. Fig. S2 and S3. Plots of mapping depth of Hifi reads. Fig. S4. Plots of LAI score. Fig. S5. Structural variations among the four chromosomes of autotetraploid genome. Fig. S6. Whole-genome duplication results of C. paliurus. Fig. S7– S10. DXY and Ks results of C. paliurus. Fig. S11 and S13. GO enrichment results. Fig. S12. Expression levels of genes with four alleles. Fig. S14, S15 and S22. Several genes are under positive selection. Fig. S16. The ploidy estimation results of two C. paliurus samples. Fig. S17. The results of the mixed-ploidy STRUCTURE analysis. Fig. S18 and S19. Plots depicting STRUCTURE and ENTROPY results for autotetraploid C. paliurus. Fig. S20. The SFS of simulated data and autotetraploid C. paliurus. Fig. S21. DCMS values of C. paliurus. Fig. S23. LD decay patterns of C. paliurus. Fig. S24. Statistical tests of five replicate datasets. [file 12915_2023_1668_MOESM1_ESM.pdf]

Genomic insights into biased allele loss and increased gene numbers after genome duplication in autotetraploid *Cyclocarya paliurus*

Rui-Min Yu[1], Ning Zhang[1], Bo-Wen Zhang[1], Yu Liang[1], Xiao-Xu Pang[1], Lei Cao[1], Yi-Dan Chen[1], Wei-Ping Zhang[1], Yang Yang[1], Da-Yong Zhang[1]*, Er-Li Pang[1]*, & Wei-Ning Bai[1]*

[1]State Key Laboratory of Earth Surface Processes and Resource Ecology, and Ministry of Education Key Laboratory for Biodiversity Science and Ecological Engineering, College of Life Sciences, Beijing Normal University, Beijing 100875, China.

*Corresponding authors: Da-Yong Zhang (zhangdy@bnu.edu.cn), Er-Li Pang (pangerli@bnu.edu.cn), Wei-Ning Bai (baiwn@bnu.edu.cn).

Figures

(a)

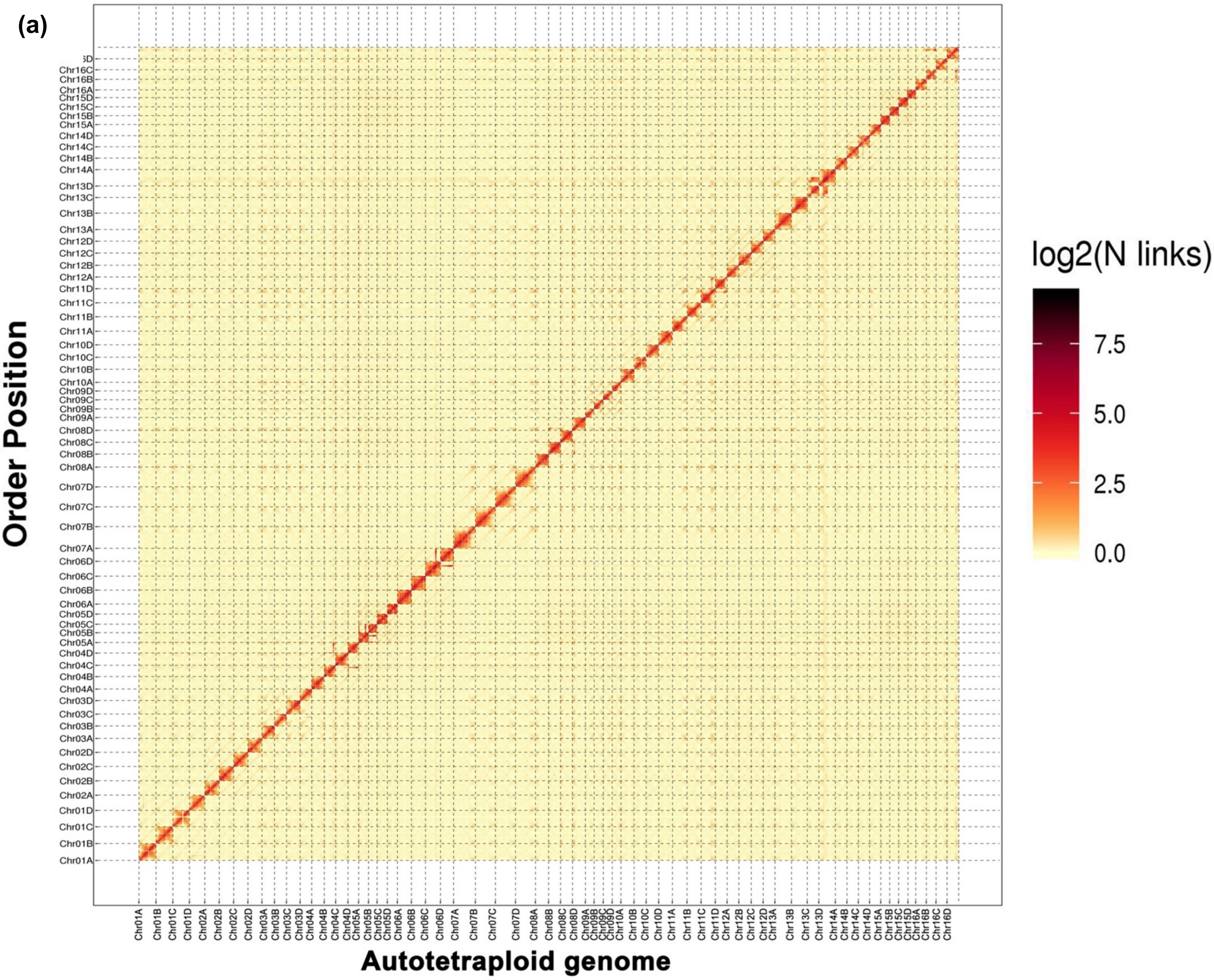
Order Position
Autotetraploid genome
log2(N links)
7.5
5.0
2.5
0.0

(b)

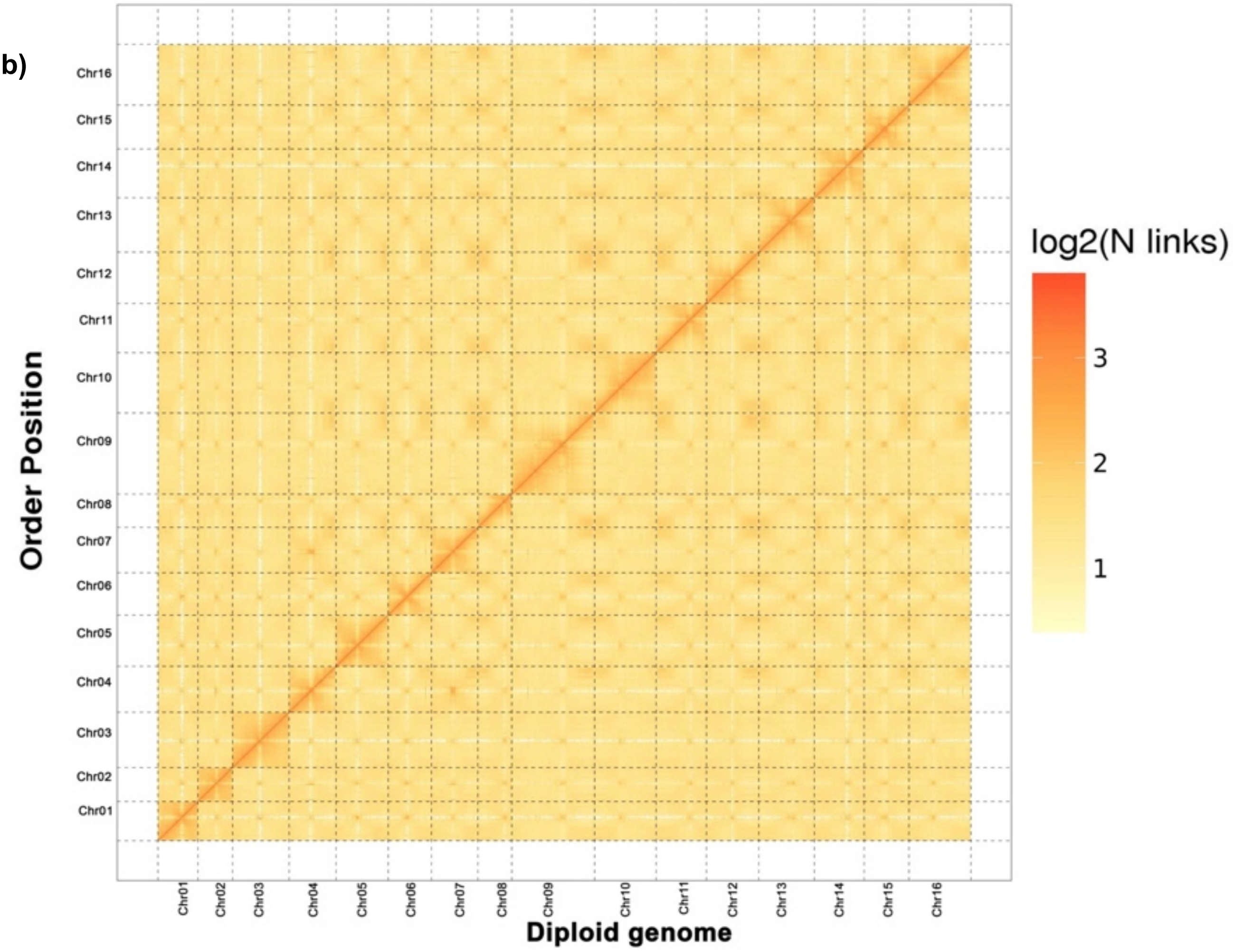

Fig. S1. The presence of strong chromatin interaction signals within the chromosomes in the Hi-C heatmaps for the tetraploid (a) and diploid (b) *Cyclocarya paliurus* genome.

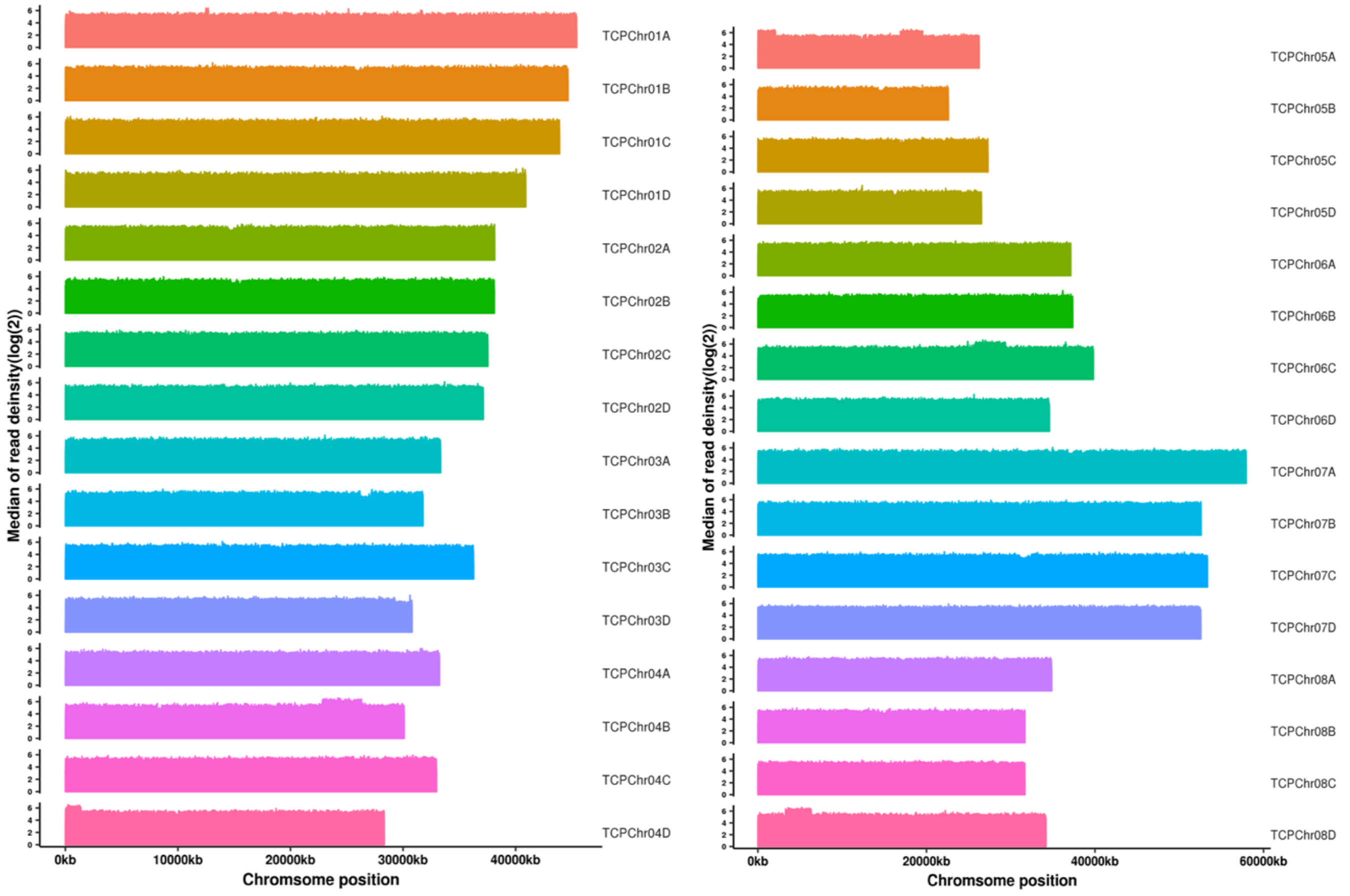

Fig. S2. The scaled depth of CCS long reads mapping to the chromosomes 1-8.

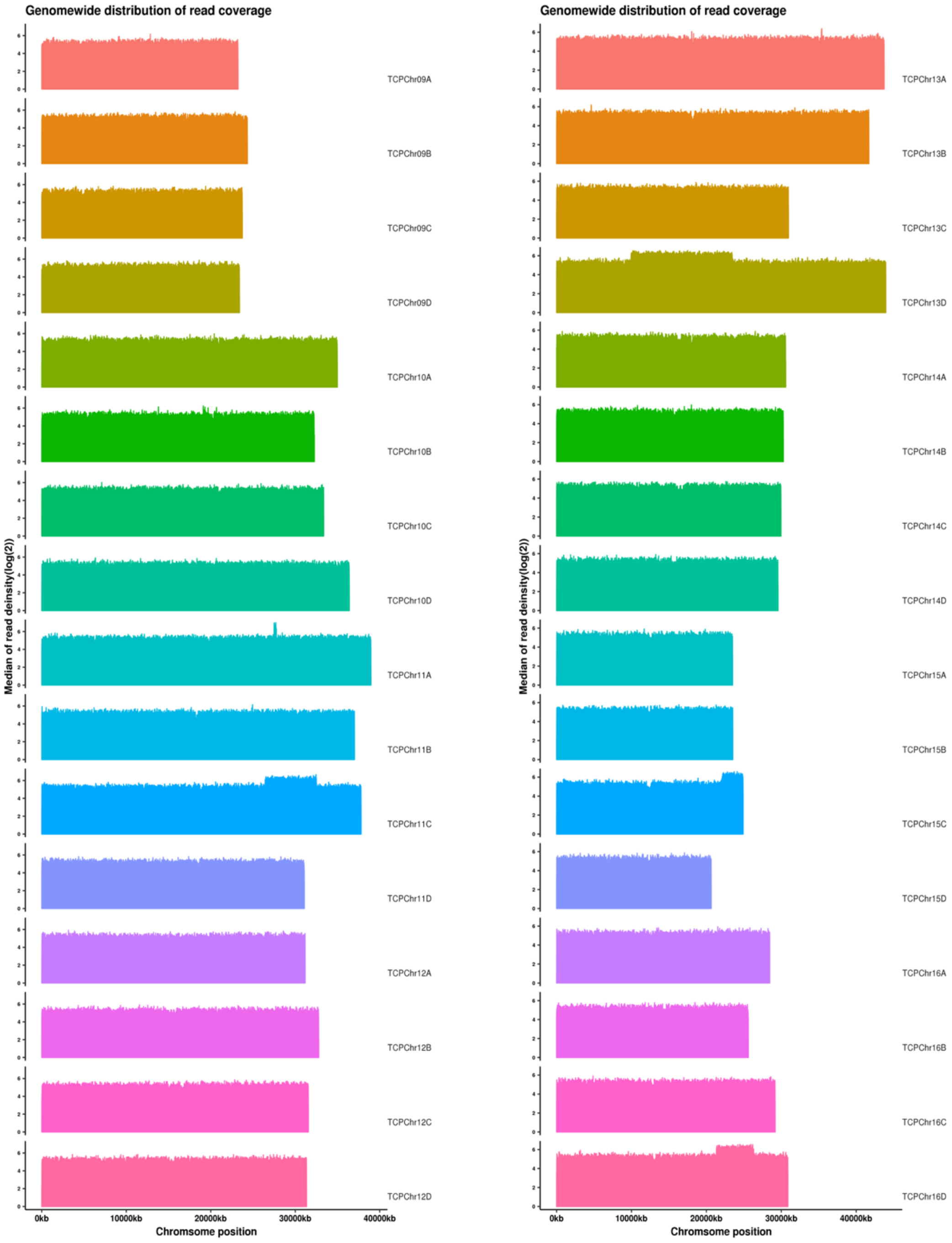

Fig. S3. The scaled depth of CCS long reads mapping to the chromosomes 9-16.

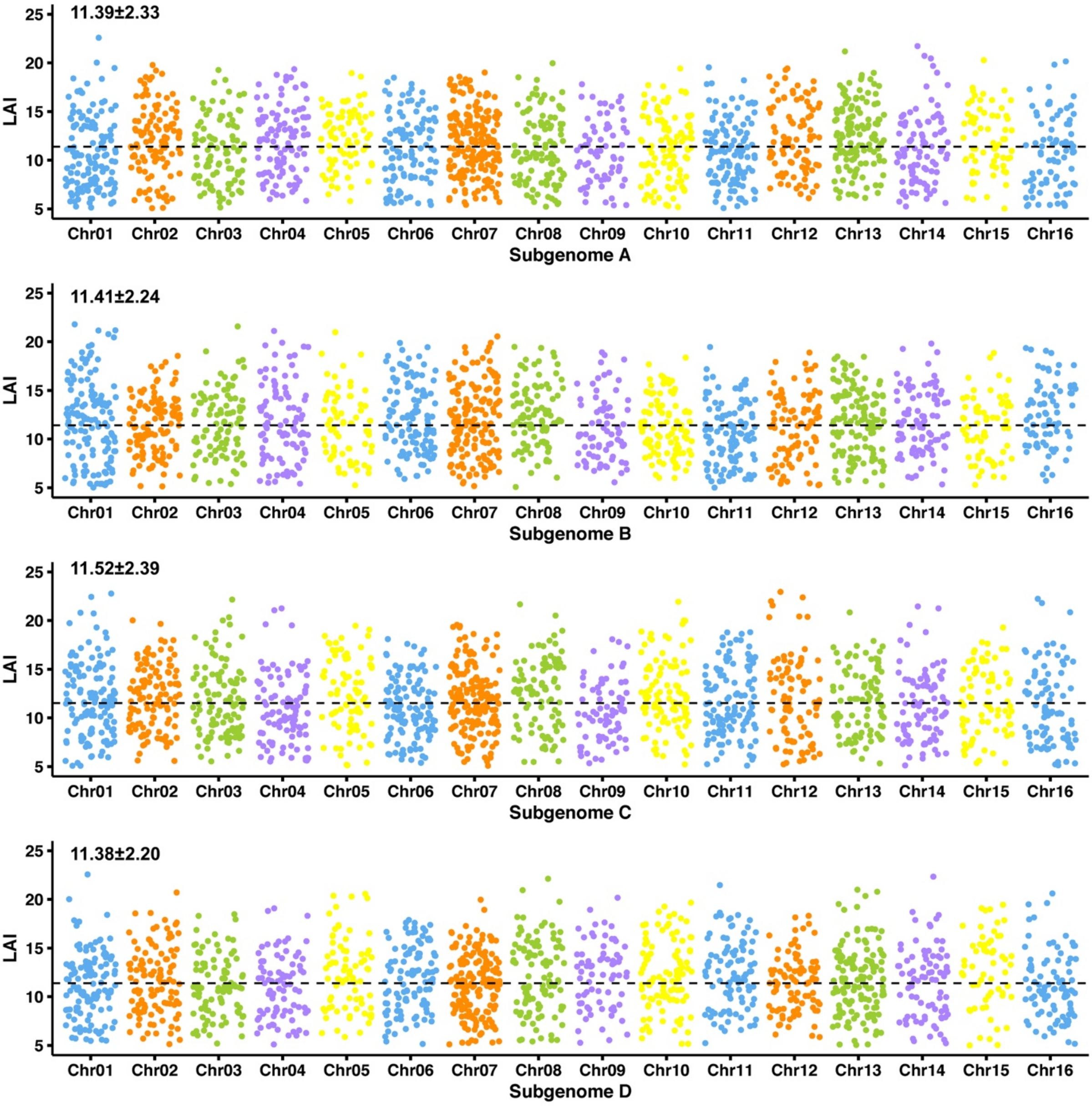

Fig. S4. The LTR assembly index (LAI) score for each chromosome was calculated by using 3000-kb sliding windows with 300-kb steps. The mean value is represented by a dotted line, and the corresponding mean ± standard deviation values are displayed in the upper left corner.

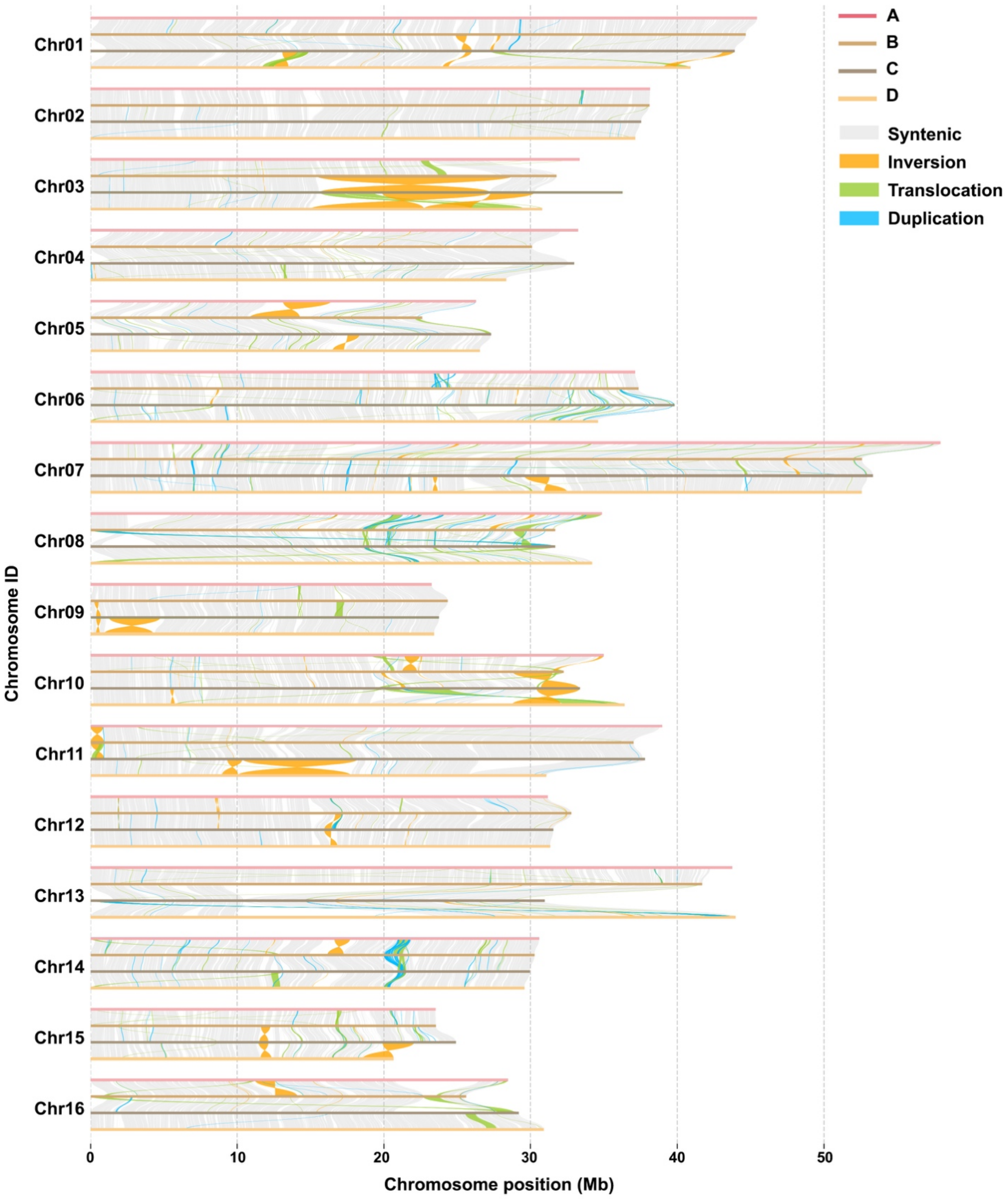

Fig. S5. Structural variations between the four allelic chromosomes for chromosome 1-16 of autotetraploid *Cyclocarya paliurus*.

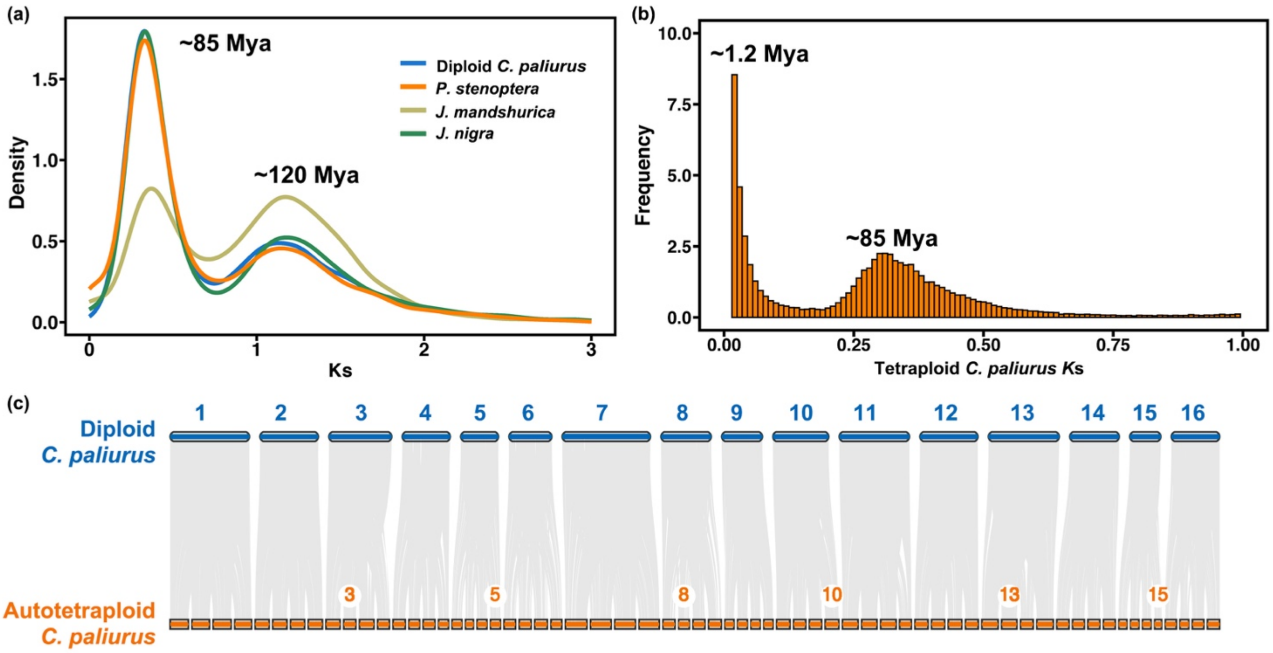

Fig. S6. Whole-genome duplication analysis of diploid and autotetraploid *C. paliurus*. (a) K_s values of diploid *C. paliurus* and three other related species. (b) K_s values of autotetraploid *C. paliurus*. (c) Collinear relationship between diploid and autotetraploid *C. paliurus* chromosomes. The collinearity pattern showed that one ancestral region in the diploid genome was traced to four regions in autotetraploid. Gray bands in the background represent syntenic blocks between the genomes spanning more than 25 genes.

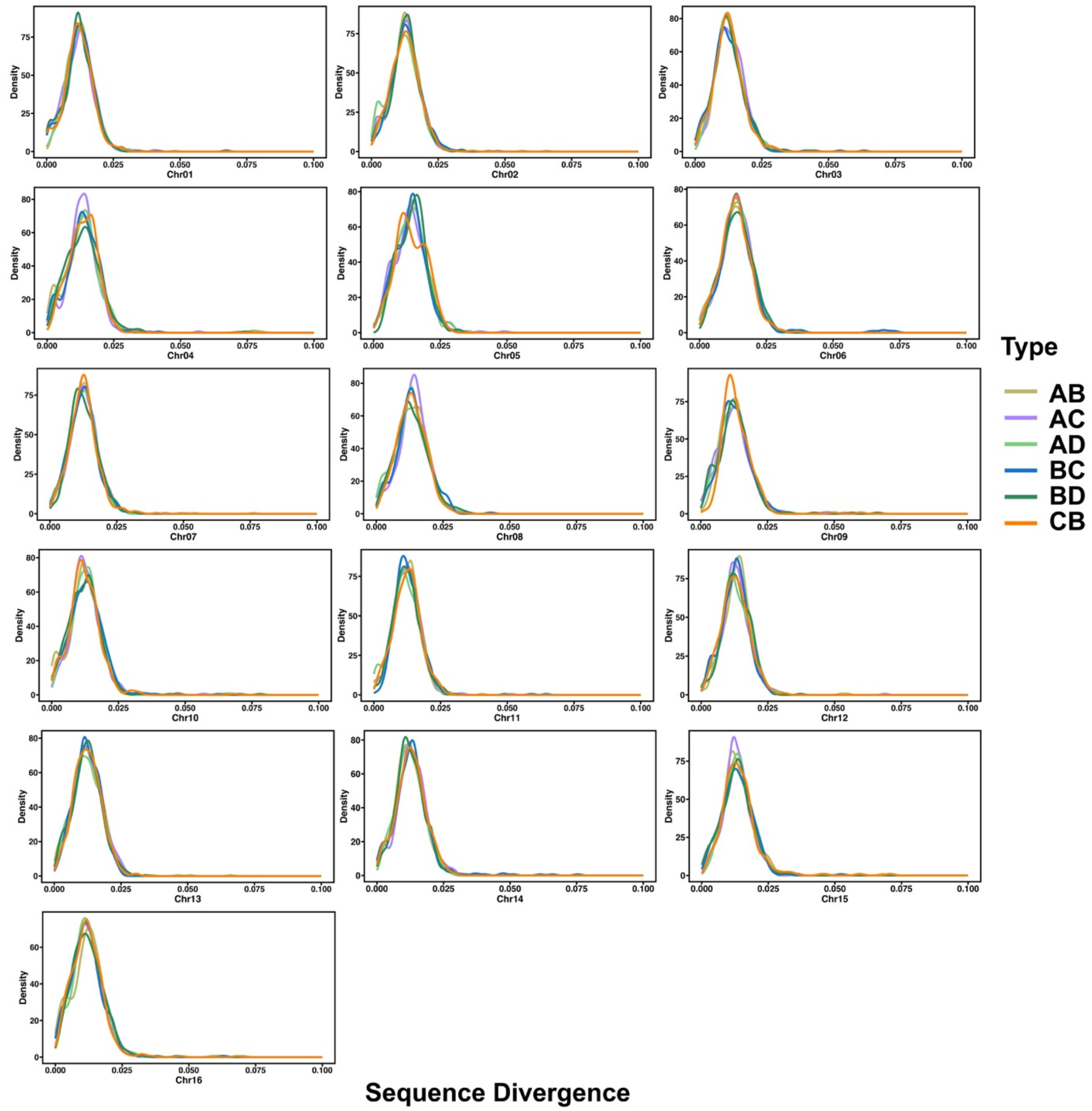

Fig. S7. Sequence divergence (D_{XY}) in 50-kb stepping windows between any two of four allelic chromosomes of autotetraploid *C. paliurus* for chromosome 1-16. Aligned sequences of any two allelic chromosomes were obtained using show-aligns function in MUMmer4.

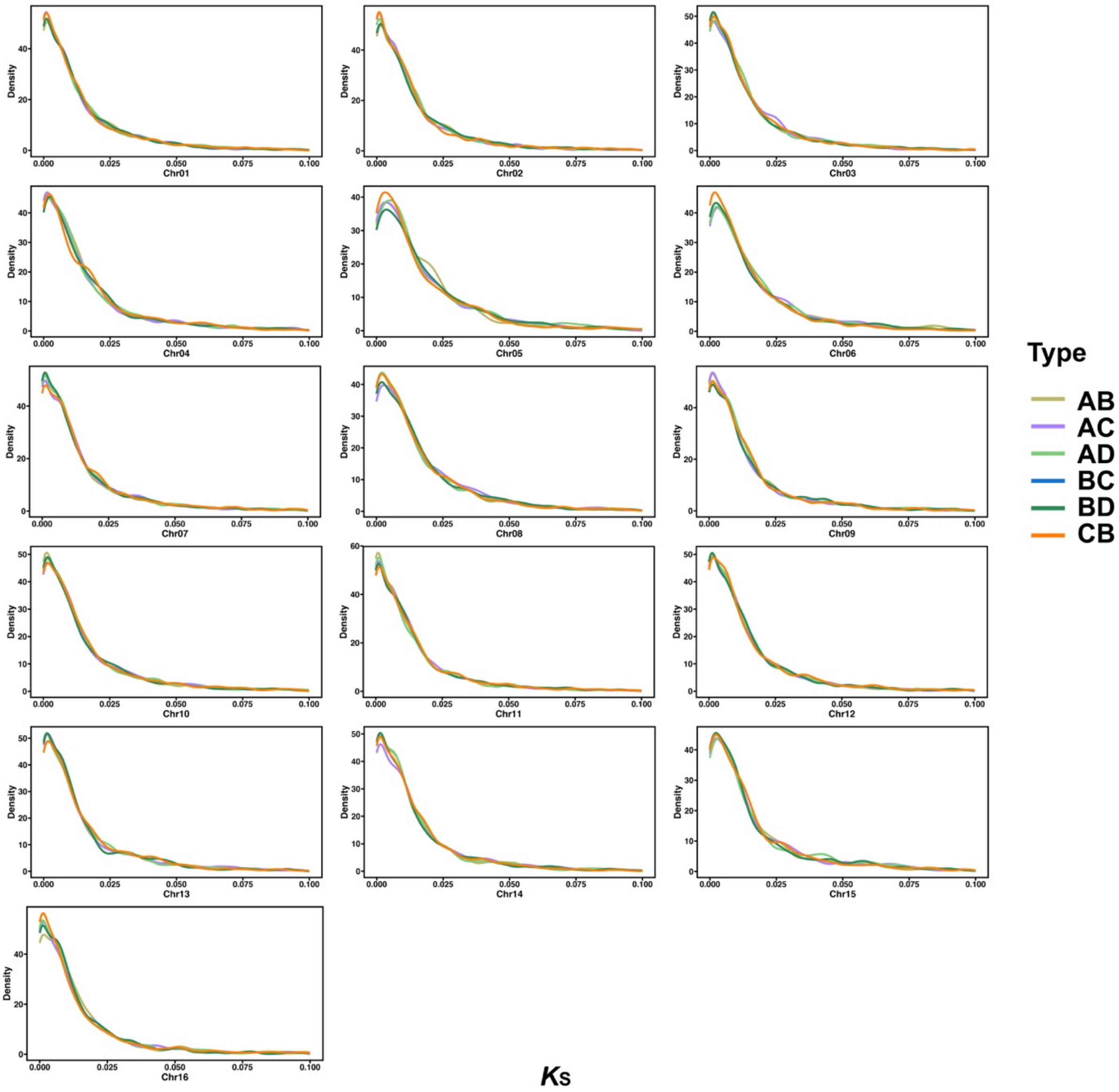

Fig. S8. K_s distribution between any two of four allelic chromosomes of autotetraploid *C. paliurus* for chromosome 1-16. Syntenic blocks between any two allelic chromosomes were identified using MCScanX based on the results of an all-to-all BLASTP search. 96 K_s distributions of syntenic genes were estimated and summarized for 16 chromosomes.

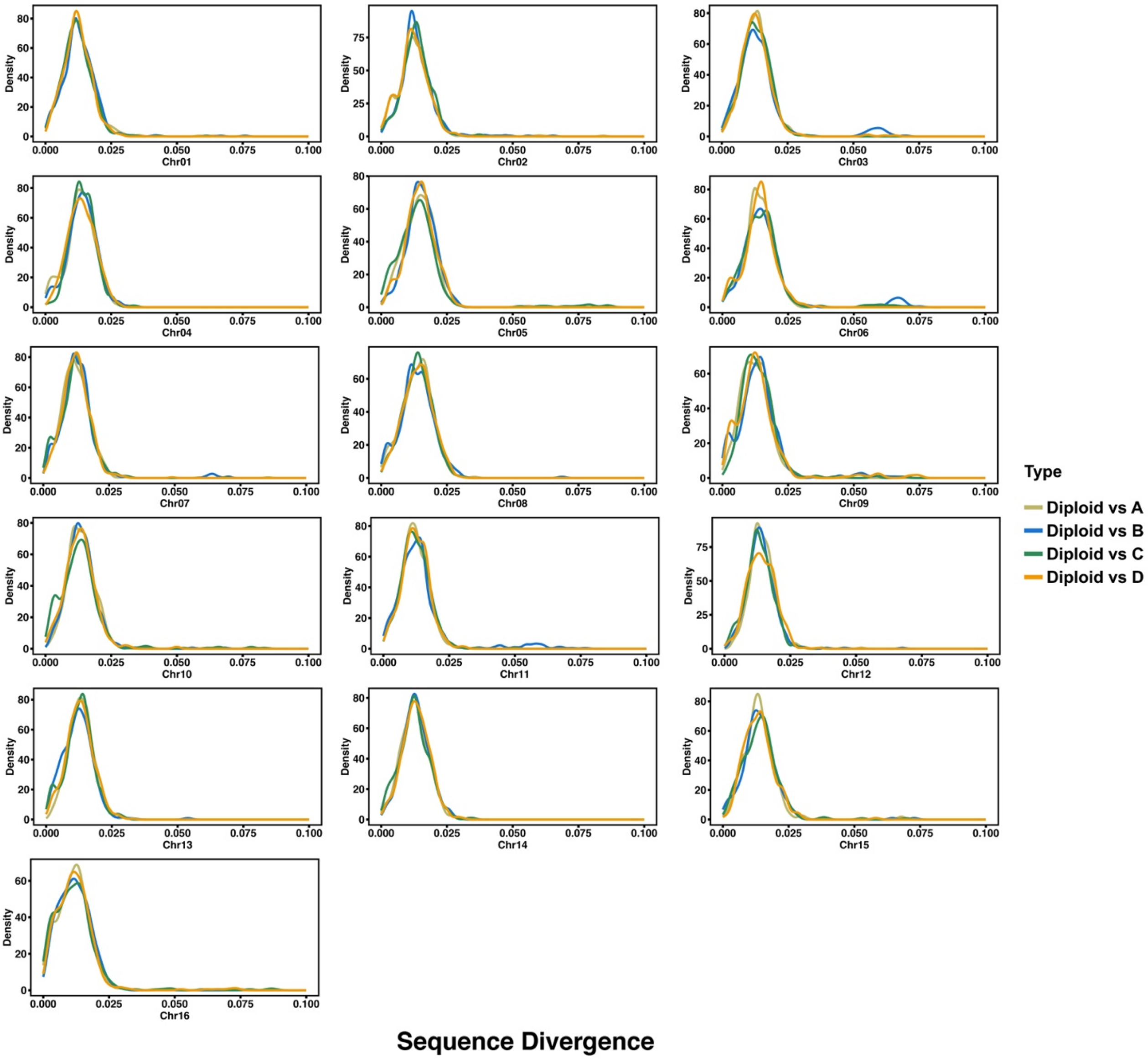

Fig. S9. D_{XY} in 50-kb stepping windows between diploid and autotetraploid *C. paliurus* for chromosome 1-16. Aligned sequences of each chromosome were obtained using show-aligns function in MUMmer4.

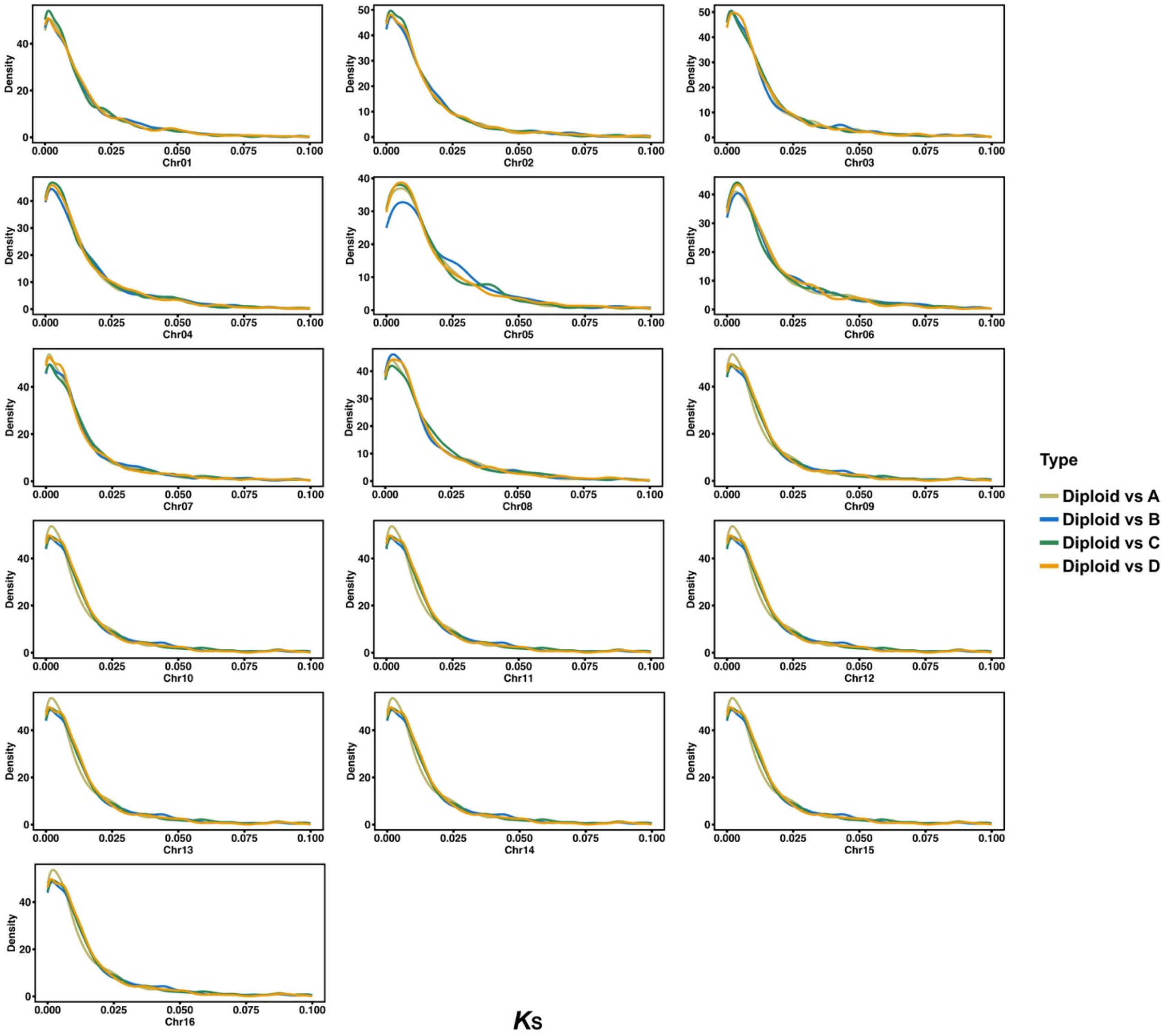

Fig. S10. K_s between diploid and autotetraploid *C. paliurus* for chromosome 1-16. Syntenic blocks between diploid and one of four allelic chromosomes were identified using MCScanX based on the results of an all-to-all BLASTP search. 64 K_s distributions of syntenic genes were estimated and summarized into 16 chromosomes.

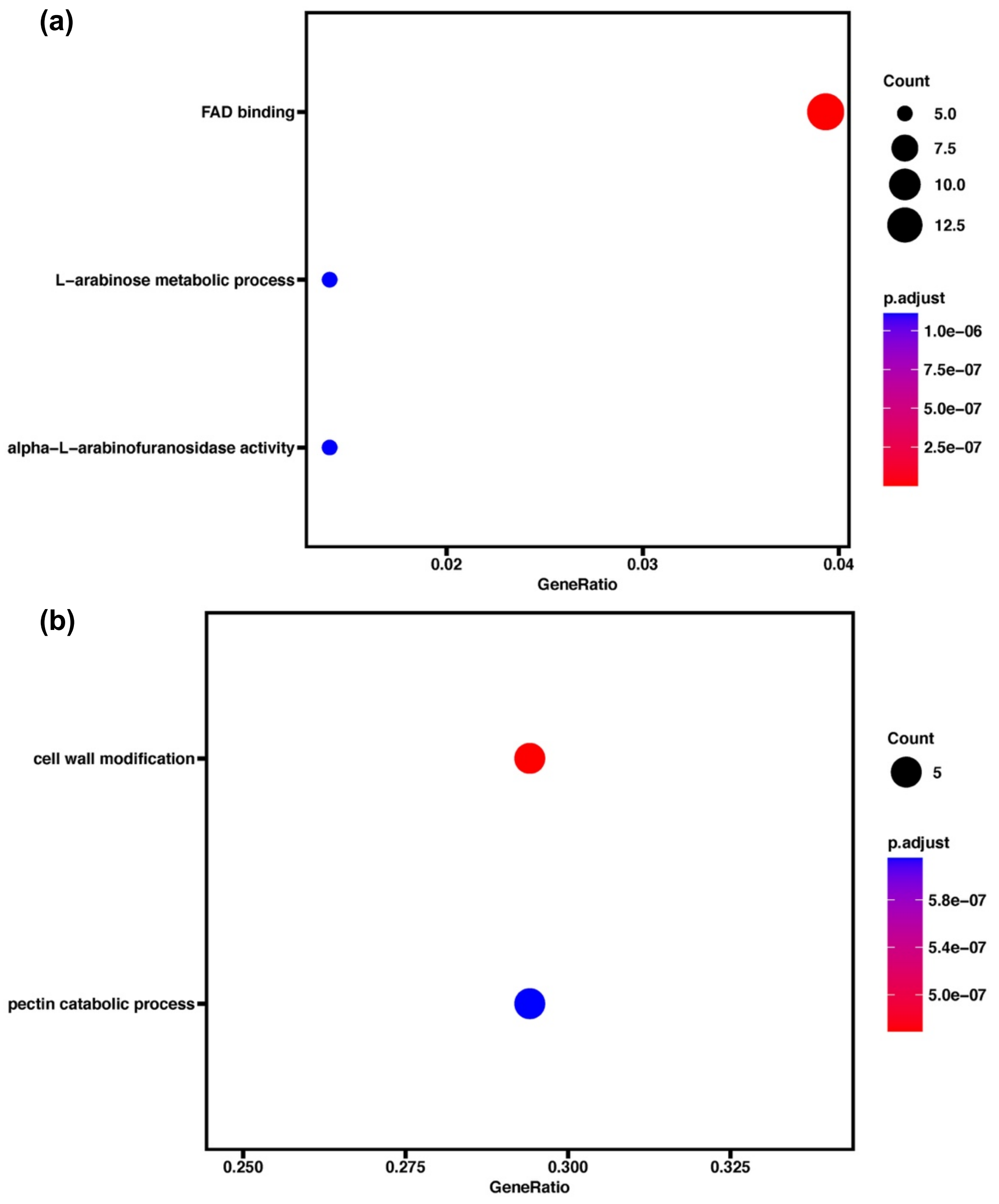

Fig. S11. GO enrichment analysis for 490 genes in the largest inversion (a) and 52 genes in the largest translocations (b) in autotetraploid genome.

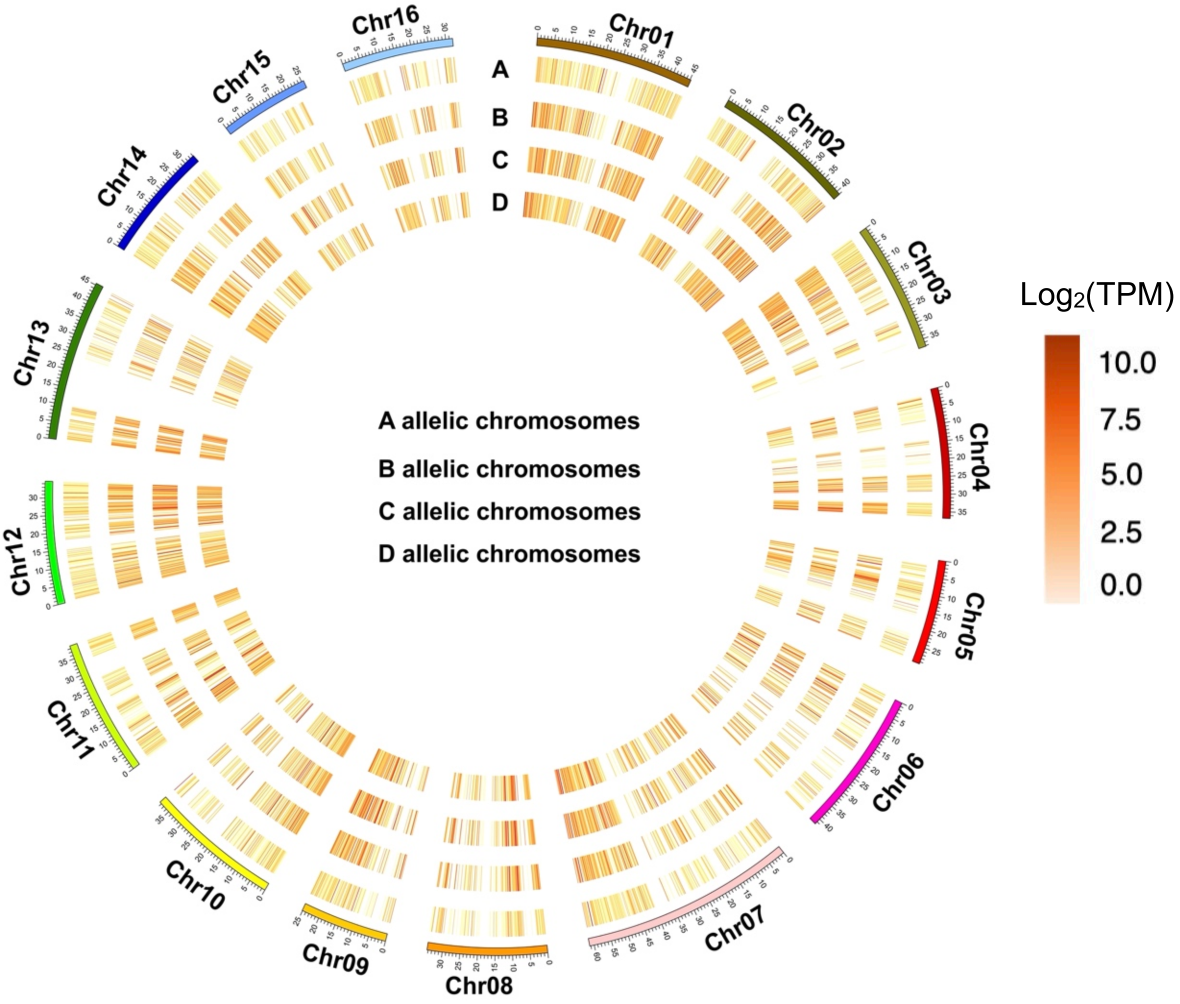

Fig. S12. Expression levels of genes with four alleles. The expression levels of genes were estimated by the transcripts per million values and calculated on the basis of unique mapping reads (TPM value). Each color line from outer to inner represents allelic chromosomes A, B, C and D.

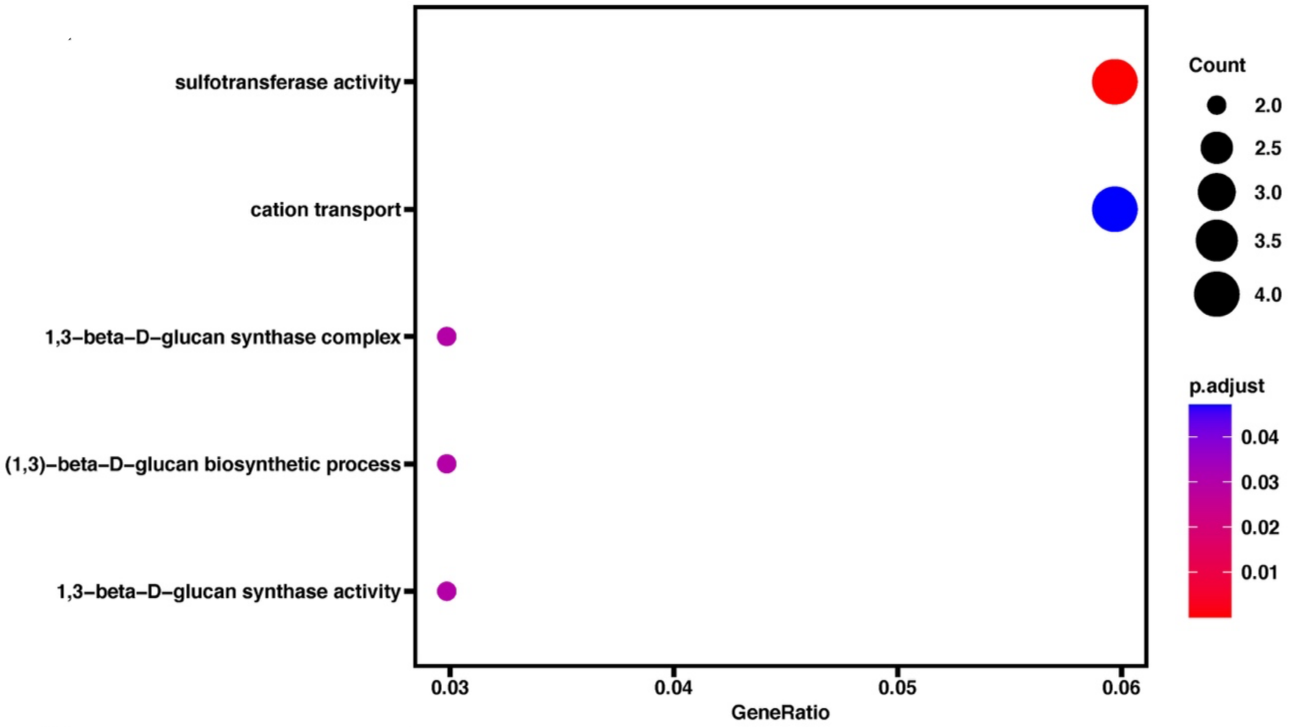

Fig. S13. GO enrichment analysis for 102 significantly upregulated genes which had four or more alleles in autotetraploid *C. paliurus*.

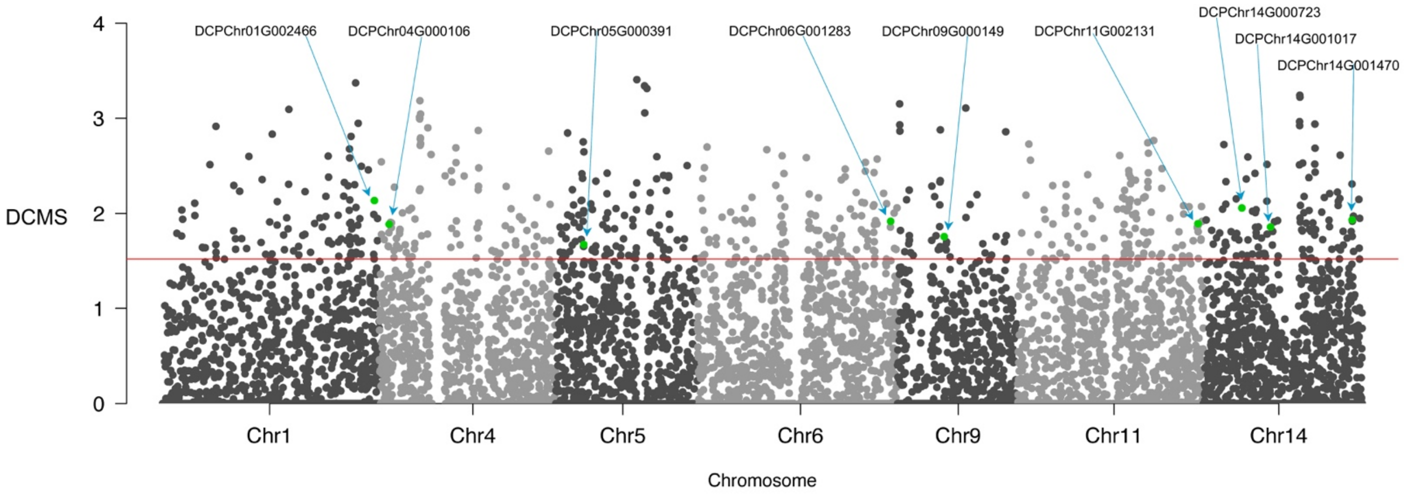

Fig. S14. Nine out of the 102 genes with four or more alleles exhibited positive selection in autotetraploid *C. paliurus.* The nine genes are marked by green dots. A minimum DCMS threshold was represented by the red line.

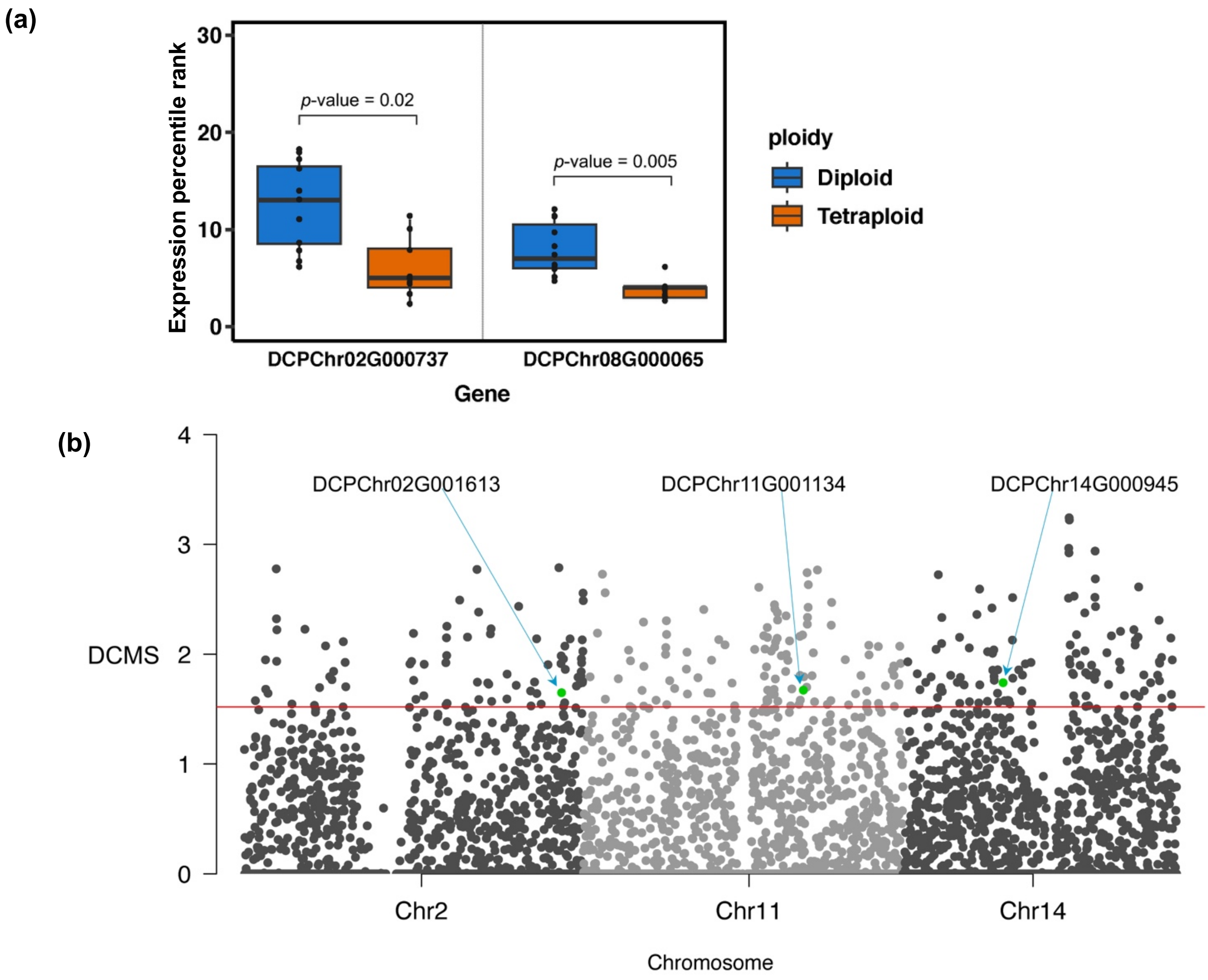

Fig. S15. Five cold-regulated (*COR*) genes in autotetraploid *C. paliurus.* (a) Expression of two *COR* genes had four or more alleles were significantly upregulated. (b) Three *COR* genes with four or more alleles were under positive selection in autotetraploid *C. paliurus*. The three genes are marked by green dots. A minimum DCMS threshold was represented by the red line.

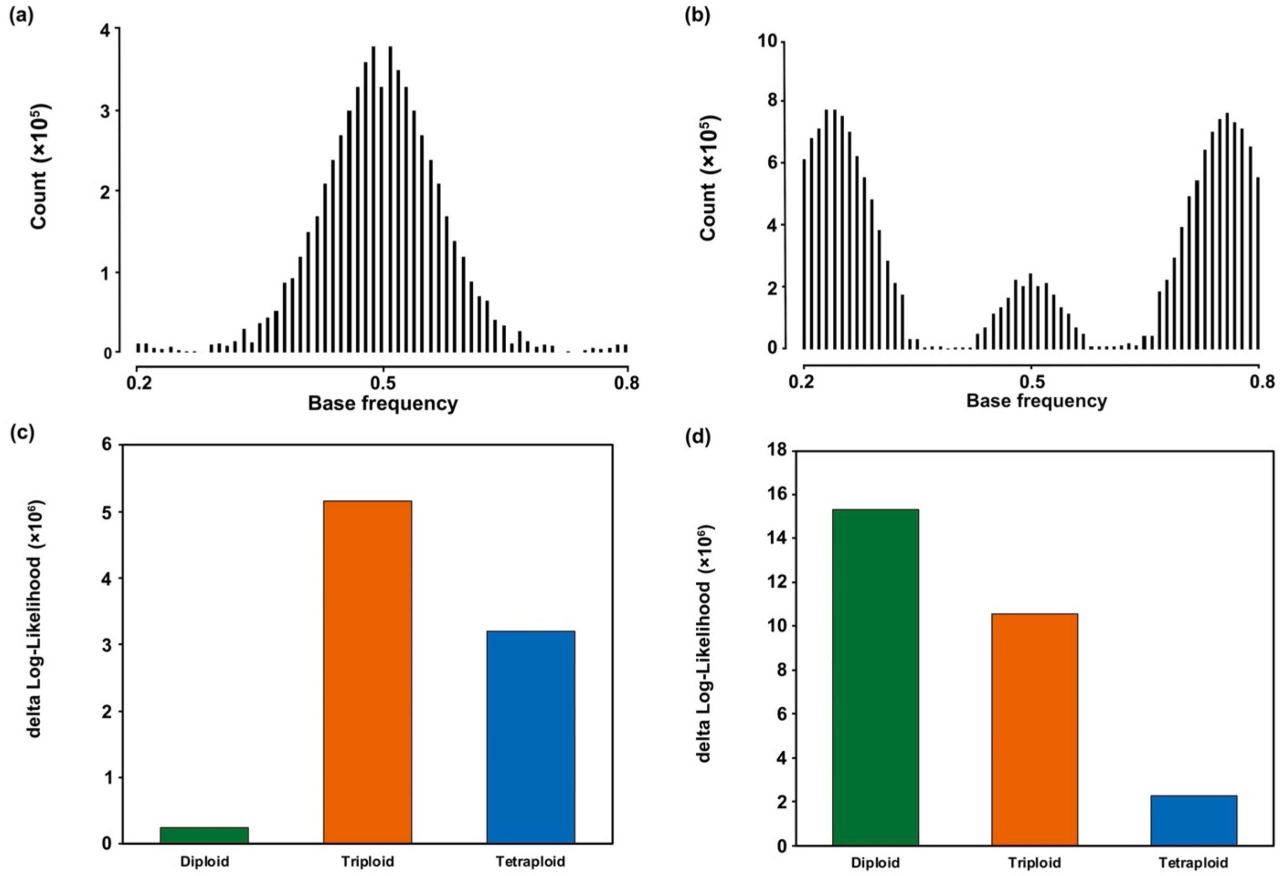

Fig. S16. Distribution of base frequencies at variable sites where only two bases are segregating for diploid (a) and tetraploid (b). The bar plots depict the Δlog*L* of all fixed models (diploid, triploid and tetraploid) for the diploid (c) and tetraploid (d).

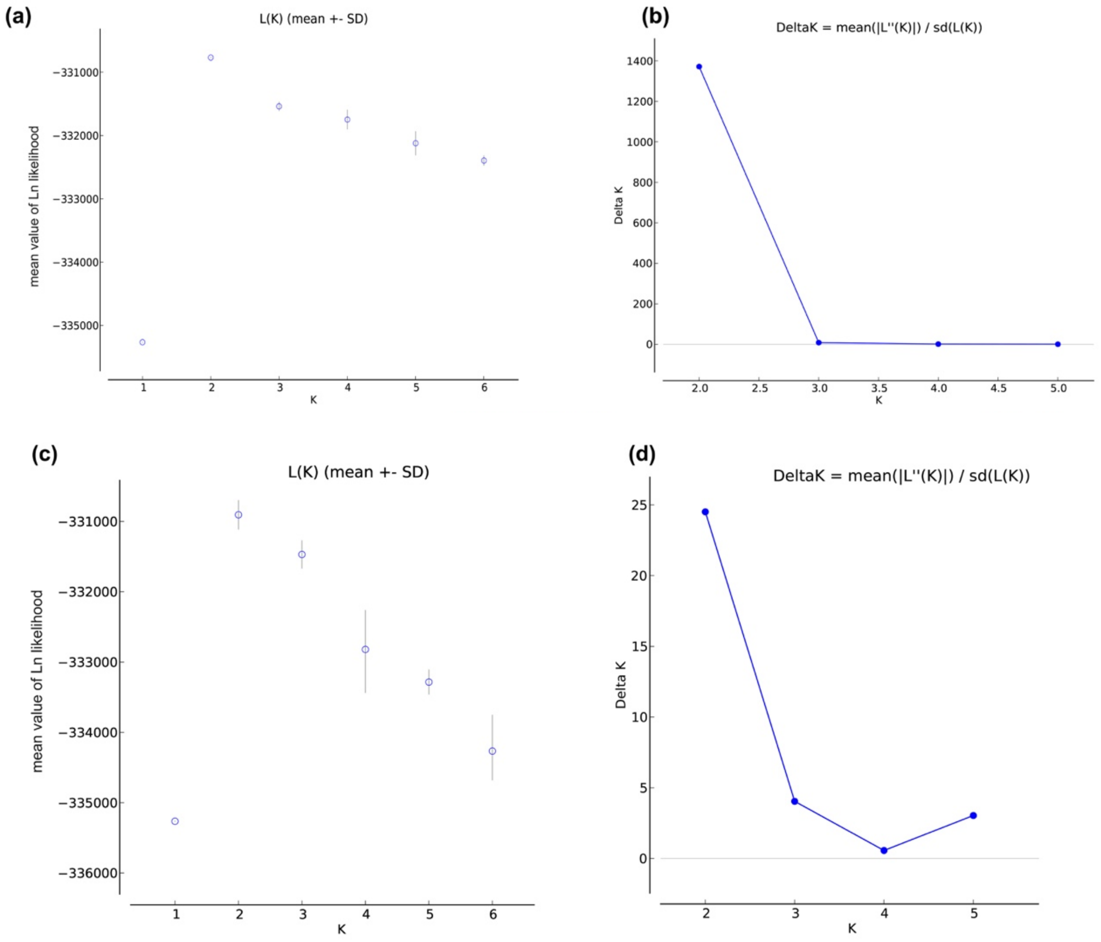

Fig. S17. The results of the mixed-ploidy STRUCTURE analysis using 2,849 independent and neutral SNPs (a, b) and 14,365 independent and synonymous SNPs (c, d) for the 118 individuals. (a, c) are mean value of Ln likelihood, and (b,d) delta *K* for 118 samples, which indicated that k = 2 was the optimal number of groups.

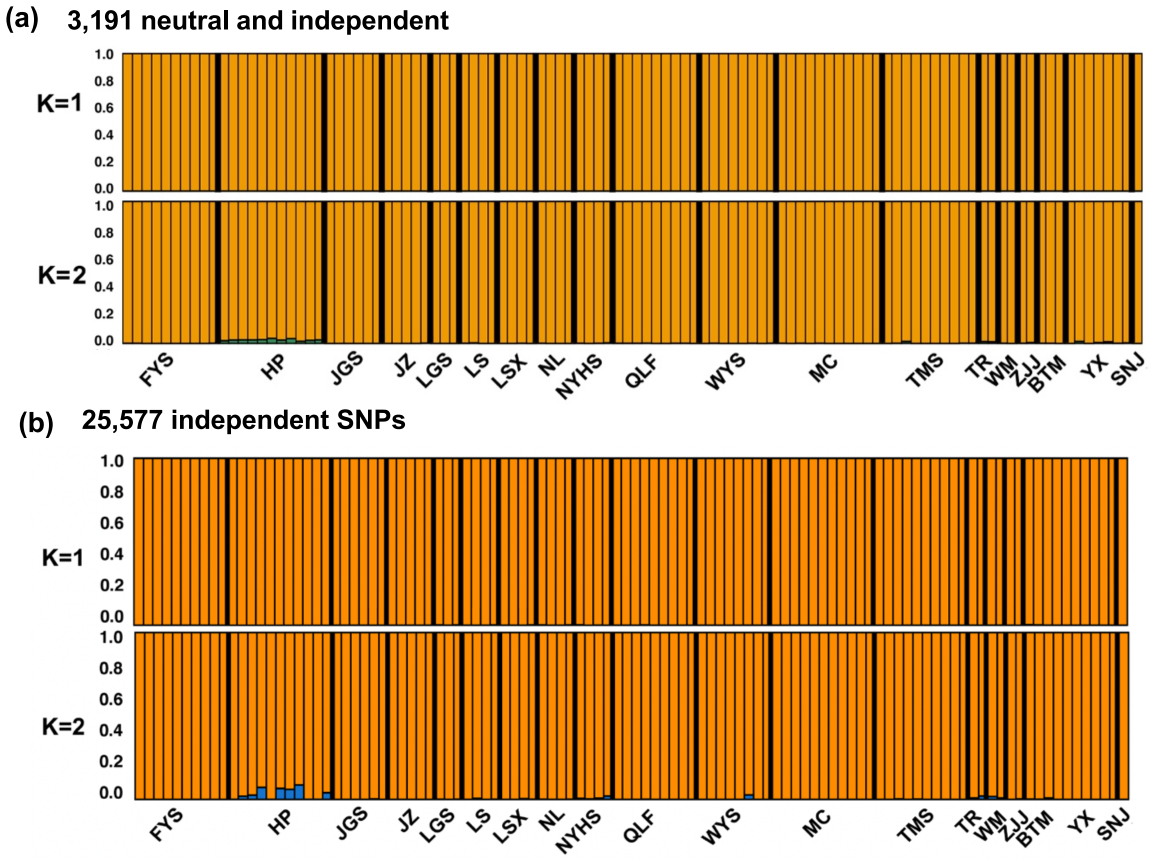

Fig. S18. STRUCTURE analysis for 106 autotetraploid *C. paliurus* samples. The results for K = 1-2 were obtained from 3,191 neutral and independent SNPs (excluding CDS regions) (a), and 25,577 independent SNPs (including CDS regions) (b).

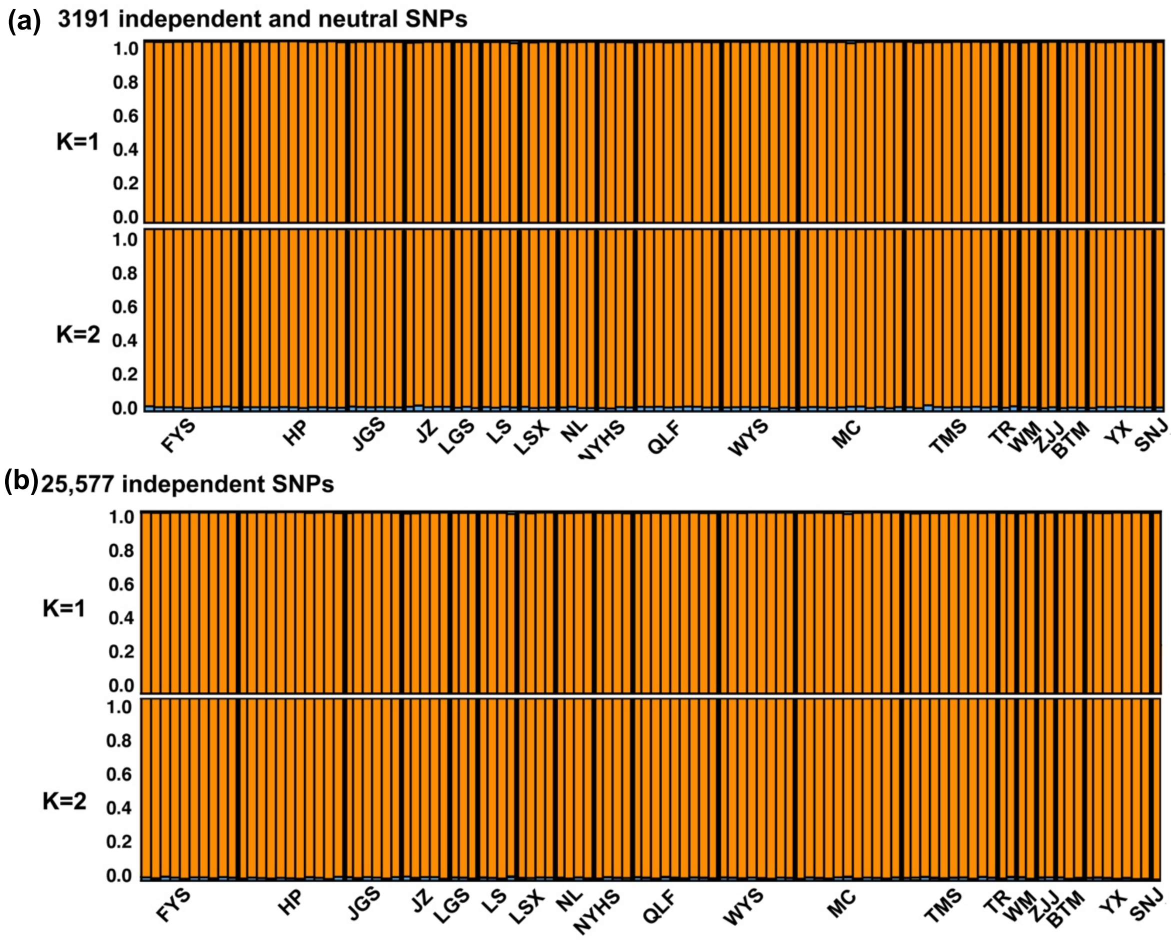

Fig. S19. ENTROPY analysis for 106 autotetraploid *C. paliurus* samples. The results for K = 1-2 were obtained from 3,191 neutral and independent SNPs (excluding CDS regions) (a), and 25,577 independent SNPs (including CDS regions) (b).

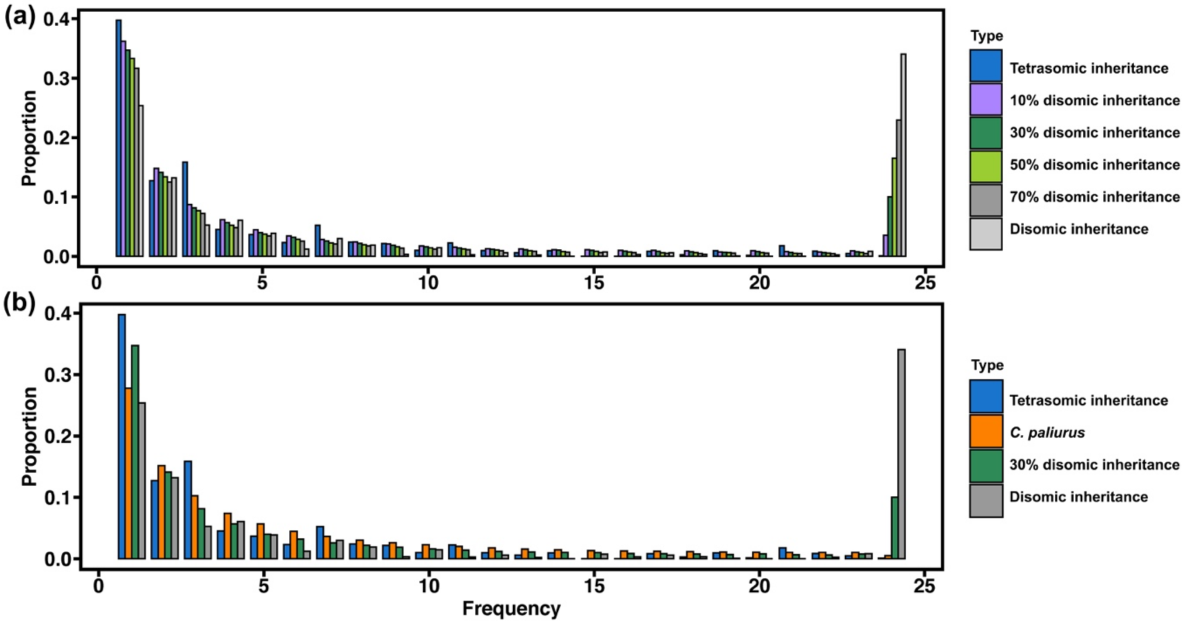

Fig. S20. The site frequency spectrum (SFS) of simulated data and autotetraploid *C. paliurus*. (a) Expected neutral folded SFS from coalescent simulations under different inheritance models (fully disomic inheritance; 10%, 30%, 50%, 70% proportions of genome exhibiting disomic inheritance; fully tetrasomic inheritance) using simulated data. (b) Folded SFS from simulated data and autotetraploid.

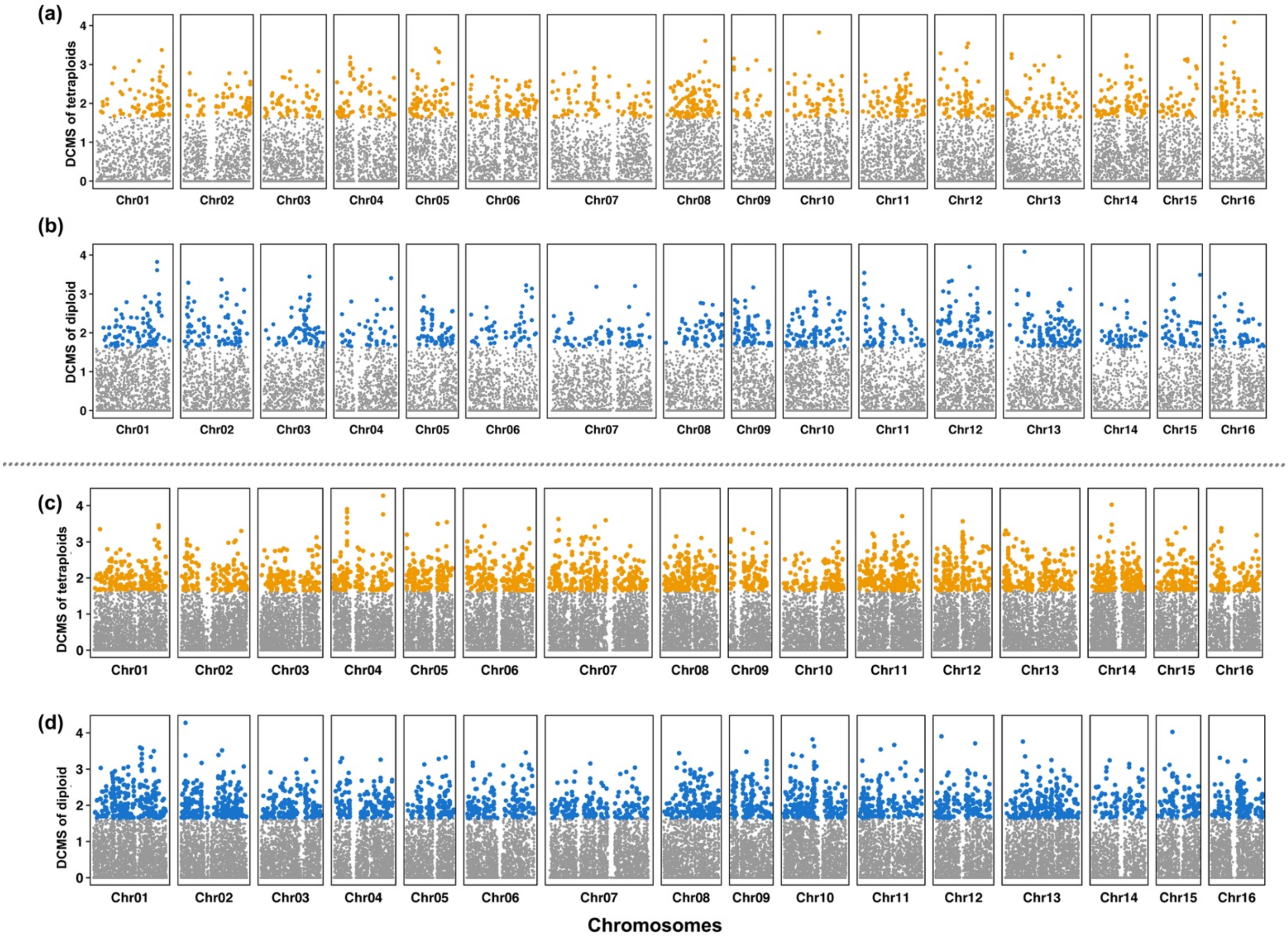

Fig. S21. DCMS values of diploid and autotetraploid *C. paliurus* with 25 kb (a, b) and 10 kb window (c, d). The regions under positive selection were in orange (tetraploid) and blue (diploid), and the regions not under selection were in gray.

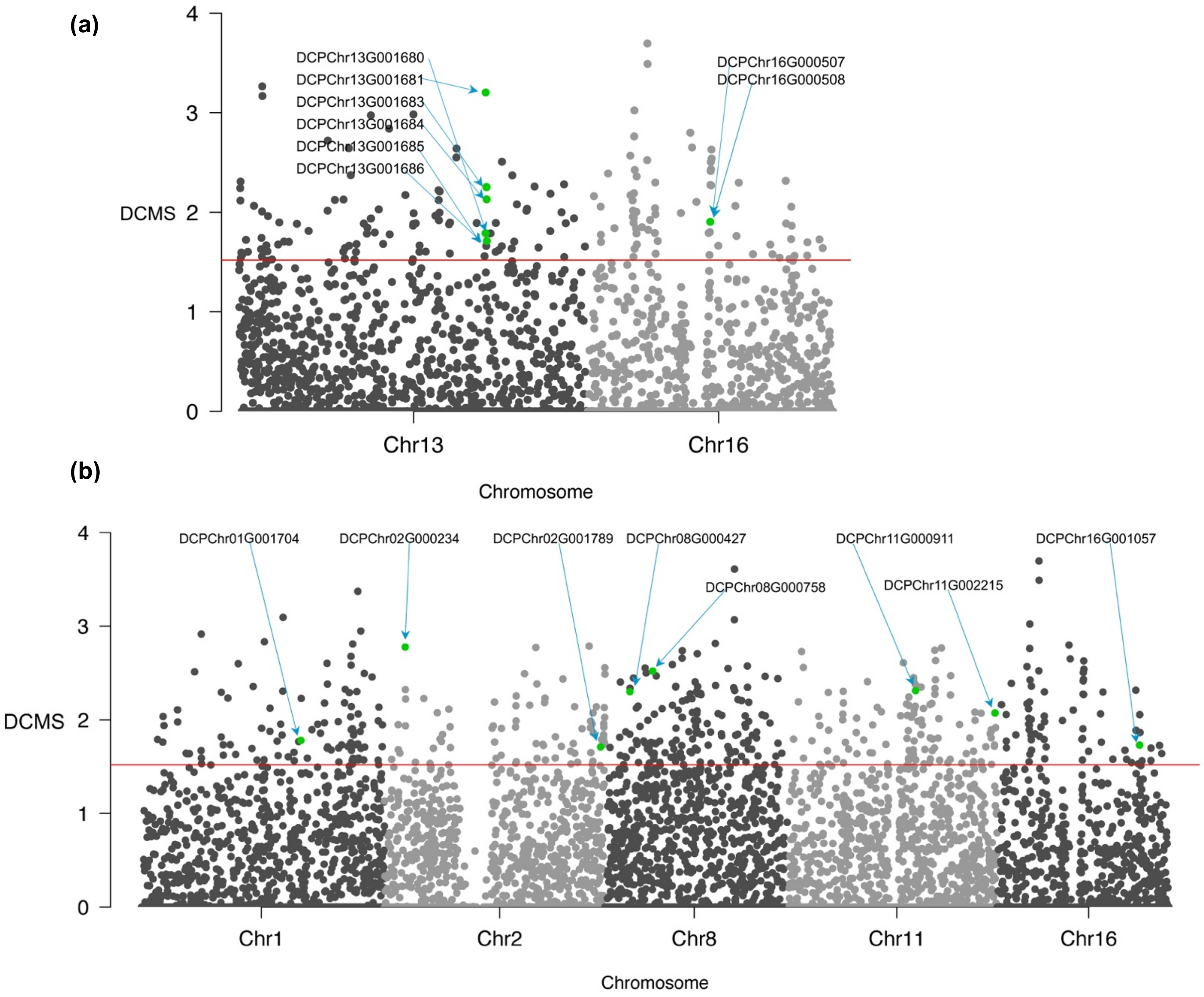

Fig. S22. (a) Eight genes under positive selection were significantly enriched for the term 'response to biotic stimulus' and (b) eight genes under positive selection were meiosis-related genes.

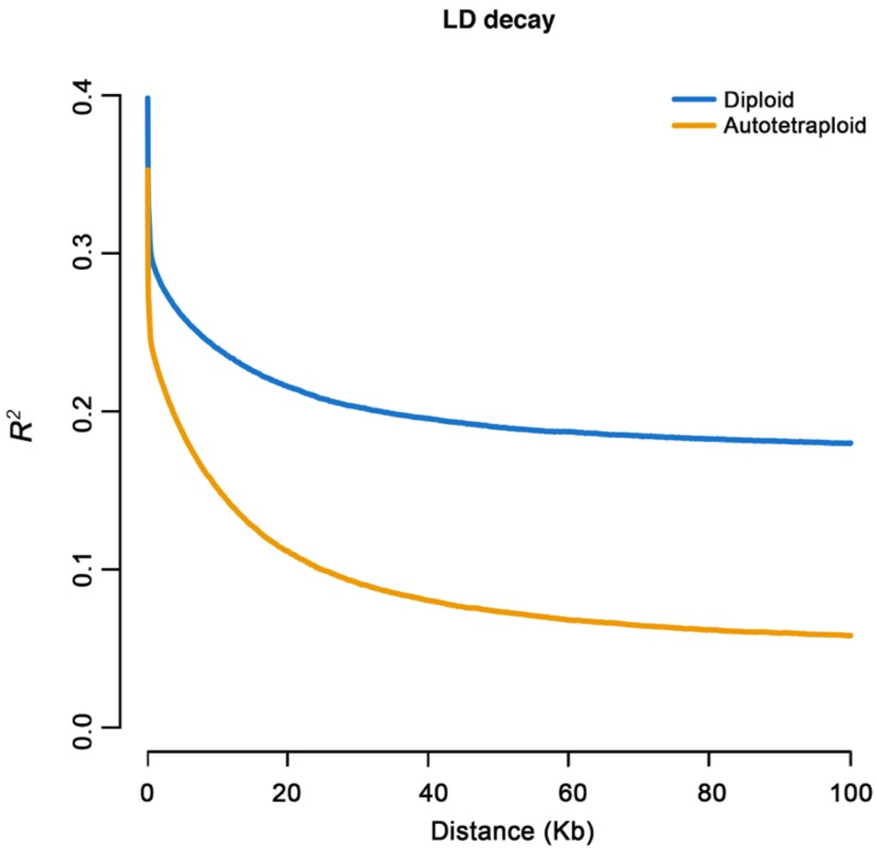

Fig. S23. Linkage disequilibrium (LD) decay patterns of diploid and autotetraploid *C. paliurus*.

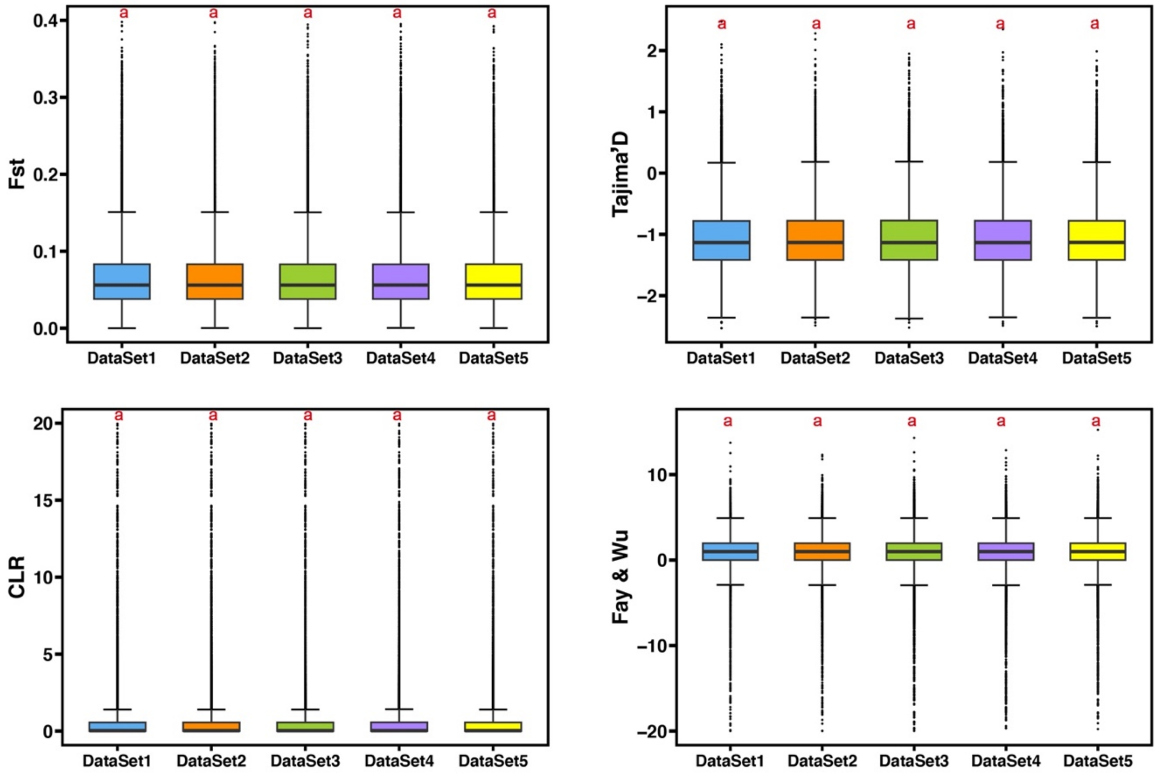

Fig. S24. Statistical tests for positive selection were conducted on five replicate datasets. The significances of five datasets for four statics were analyzed using the rank sum test. The red labels indicate that there is no significant difference between the five replicate datasets, with *p* -value > 0.05.
