# Supplementary material for: Genomic insights into biased allele loss and increased gene numbers after genome duplication in autotetraploid Cyclocarya paliurus
Source: BMC Biol. 2023 Aug 8;21:168. doi: 10.1186/s12915-023-01668-1 (PMC10408227; doi:10.1186/s12915-023-01668-1)
Supplement: Supplementary file 2 — Additional file 2: Table S1. BUSCO and CEGMA assessments. Table S2. The coverage rates of Illumina reads and Hifi reads mapping to autotetraploid genome. Table S3 and S6. Summaries of repetitive elements for C. paliurus. Table S4. The length and gene count of autotetraploid genome. Table S5 and S7. Number of SNPs and SVs in autotetraploid genome. Table S8. Allele annotations of autotetraploid C. paliurus. Table S9 and S10. Summary numbers of genes with/without allele loss in SVs, TEs and PPIs. Table S12. COR genes with four or more alleles of autotetraploid C. paliurus. Table S13. Sampling and mapping details of 118 C. paliurus individuals. Table S14. Ploidy estimation for 118 individuals. Table S15. Inheritance of autotetraploid C. paliurus. Table S16 and S18. Information on several genes under positive selection. Table S17. The 62 meiosis-related genes in C. paliurus. Table S19. Number of remaining SNPs after filtering. Table S20. Mapping information for allele expression analysis. Table S21. Several samples were used for RNA-seq analysis. [file 12915_2023_1668_MOESM2_ESM.pdf]

Genomic insights into biased allele loss and increased gene numbers after genome duplication in autotetraploid *Cyclocarya paliurus*

Rui-Min Yu[1], Ning Zhang[1], Bo-Wen Zhang[1], Yu Liang[1], Xiao-Xu Pang[1], Lei Cao[1], Yi-Dan Chen[1], Wei-Ping Zhang[1], Yang Yang[1], Da-Yong Zhang[1]*, Er-Li Pang[1]*, & Wei-Ning Bai[1]*

[1]State Key Laboratory of Earth Surface Processes and Resource Ecology, and Ministry of Education Key Laboratory for Biodiversity Science and Ecological Engineering, College of Life Sciences, Beijing Normal University, Beijing 100875, China.

*Corresponding authors: Da-Yong Zhang (zhangdy@bnu.edu.cn), Er-Li Pang (pangerli@bnu.edu.cn), Wei-Ning Bai (baiwn@bnu.edu.cn).

Tables

Table S1. BUSCO and CEGMA assessment about autotetraploid and diploid genomes assembly.

Genomes	Complete BUSCOs (C)	Complete and single-copy BUSCOs (S)	Complete and duplicated BUSCOs (D)	Fragmented BUSCOs (F)	Missing BUSCOs (M)	Total Lineage BUSCOs	Number of 248 highly conserved CEGs present	% of 248 highly conserved CEGs present
**Autotetraploid**	1,563 (96.84%)	73 (4.52%)	1,490 (92.32%)	9 (0.56%)	42 (2.60%)	1,614	220	88.71%
**Diploid**	1,581 (97.96%)	1,473 (91.26%)	108 (6.69%)	14 (0.87%)	19 (1.18%)	1,614	222	89.52%

Table S2. The coverage rates of next-generation Illumina short reads Hifi long reads mapping to the assembled autotetraploid genome.

Type	Total reads	Mapped reads	Mapped rate (%)	Properly mapped reads	Properly mapped rate (%)
Illumina short reads	262,987,708	254,626,915	96.82	243,905,532	93.81
Hifi long reads	5,018,813	5,009,904	99.82	—	—

Table S3. Summaries of repetitive elements for autotetraploid *C. paliurus* genome.

	Type	Length (bp)	Rate (%)
**TE (Transposon element)**	LTR	682,131,392	28.95
	LINE	123,719,901	5.25
	SINE	3,646,209	0.15
	DIRS	448,350	0.03
	DNA transposon	185,154,083	7.86
**Tandem repeats**	Tandem repeats	153,695,597	6.52
**Total**		1,148,795,532	48.76

Table S4. The length and number of genes of four allelic chromosomes.

	length of allelic chromosome (Mb)				Number of genes			
	A	B	C	D	A	B	C	D
**Chr01**	45.42	44.66	43.91	40.90	3,213	3,102	3,183	2,981
**Chr02**	38.14	38.11	37.54	37.13	2,463	2,500	2,482	2,499
**Chr03**	33.34	31.77	36.27	30.80	2,486	2,148	2,555	2,087
**Chr04**	33.24	30.11	32.98	28.33	2,160	1,877	2,068	1,749
**Chr05**	26.26	22.60	27.31	26.53	1,716	1,400	1,722	1,741
**Chr06**	37.12	37.36	39.81	34.60	2,040	2,093	2,230	1,909
**Chr07**	57.95	52.59	53.35	52.56	3,803	3,653	3,660	3,587
**Chr08**	34.87	31.68	31.68	34.19	2,127	1,897	2,100	2,253
**Chr09**	23.23	24.34	23.74	23.40	1,588	1,714	1,619	1,627
**Chr10**	34.99	32.26	33.37	36.41	2,348	2,149	2,155	2,400
**Chr11**	38.97	37.02	37.80	31.08	2,762	2,712	2,726	2,057
**Chr12**	31.17	32.78	31.56	31.34	2,287	2,311	2,394	2,259
**Chr13**	43.74	41.70	30.97	43.96	2,862	2,693	2,233	2,902
**Chr14**	30.60	30.28	29.96	29.57	2,138	2,067	2,094	2,077
**Chr15**	23.50	23.53	24.91	20.64	1,419	1,466	1,495	1,221
**Chr16**	28.46	25.60	29.19	30.89	1,954	1,541	2,010	2,157
**Total**	561.01	536.41	544.34	532.35	37,366	35,323	36,726	35,506

Table S5. The number of SNPs and structure variations (SVs) between any two allelic chromosomes.

	Number of SNPs						Number of SVs					
	**AB**	**AC**	**AD**	**BC**	**BD**	**CD**	**AB**	**AC**	**AD**	**BC**	**BD**	**CD**
**Chr01**	381,133	358,466	355,721	364,700	351,166	337,756	221	162	139	196	157	151
**Chr02**	319,430	327,538	319,556	331,102	319,482	332,614	188	168	156	190	134	181
**Chr03**	240,198	294,888	232,688	250,912	260,584	240,301	115	123	118	108	100	86
**Chr04**	254,399	257,890	235,763	250,420	225,881	237,027	127	155	115	141	108	145
**Chr05**	152,910	190,019	195,856	150,279	157,903	203,361	103	108	108	107	107	131
**Chr06**	270,075	265,265	239,382	274,433	250,463	248,211	164	215	217	213	195	237
**Chr07**	419,518	422,526	430,694	419,066	417,313	418,028	320	312	298	305	305	276
**Chr08**	217,440	246,362	242,800	208,463	219,891	242,879	198	167	205	129	176	175
**Chr09**	210,421	205,587	201,783	207,361	200,597	212,033	63	77	73	66	66	82
**Chr10**	255,211	270,647	289,649	263,186	263,390	261,517	122	118	138	120	132	122
**Chr11**	324,630	326,765	259,563	328,367	258,738	261,769	139	188	84	171	83	97
**Chr12**	292,086	285,880	289,172	282,545	278,104	282,766	150	151	114	159	109	109
**Chr13**	348,967	221,212	352,472	213,863	331,527	227,882	173	127	209	133	186	156
**Chr14**	237,432	230,892	250,985	234,439	237,135	226,343	149	139	113	142	92	120
**Chr15**	176,093	178,703	160,608	176,305	160,059	160,337	104	102	72	137	137	78
**Chr16**	194,303	238,221	248,524	198,828	205,530	242,533	104	98	96	93	93	89
**Total**	4,294,246	4,320,861	4,305,216	4,154,269	4,137,763	4,135,357	2,440	2,410	2,255	2,410	2,180	2,235

Table S6. Summaries of repetitive elements for diploid *C. paliurus* genome.

	**Type**	**Length (bp)**	**Rate (%)**
**TE (Transposon element)**	LTR	211,844,603	35.22
	LINE	47,275,569	7.86
	SINE	669,075	0.11
	DNA transposon	44,690,342	7.43
	Others	22,101,173	3.68
**Tandem repeats**	Tandem repeats	7,323,032	1.22
**Total**		333,903,794	55.52

Table S7. The number and length of SVs on different sub-genome (A, B, C and D) of autotetraploid *C. paliurus*.

**Type**		**Inversions**		**Translocations**		**Duplications**	
		Number	Length (Mb)	Number	Length (Mb)	Number	Length (Mb)
A		32	5.26	989	5.82	1,705	9.02
B		37	16.02	953	6.40	1,671	8.27
C		27	2.64	985	6.59	1,695	8.17
D		27	6.76	934	10.25	1,600	7.90
Shared	AB	0	0	75	1.47	181	1.17
	AC	8	0.16	77	0.74	185	1.07
	AD	7	6.46	66	0.67	161	1.08
	BC	2	0.17	80	0.99	159	0.80
	BD	2	0.57	68	0.38	144	0.72
	CD	8	0.10	61	1.15	161	0.84
	ABC	3	0.31	19	0.35	69	0.45
	ABD	3	0.11	21	1.07	58	0.32
	ACD	2	0.17	16	0.31	58	0.30
	BCD	2	0.30	16	0.24	56	0.25
	ABCD	11	6.91	18	1.86	64	0.49

Table S8. Allele annotations of genes distributed in different chromosomes of autotetraploid *C. paliurus*.

	Total no. of genes with 4 alleles	Total no. of genes with 3 alleles	Total no. of genes with 2 alleles	Total no. of genes with 1 allele	No. of tandem duplicated genes	No. of dispersedly duplicated genes
**Chr01**	2,119	183	172	142	68	331
**Chr02**	1,590	138	95	129	53	276
**Chr03**	1,383	157	401	101	66	342
**Chr04**	974	357	134	112	63	337
**Chr05**	738	296	103	140	49	435
**Chr06**	963	182	129	165	76	549
**Chr07**	2,308	209	181	223	63	615
**Chr08**	1,056	339	170	140	80	530
**Chr09**	1,072	156	68	111	47	216
**Chr10**	1,357	142	243	158	61	282
**Chr11**	1,431	614	159	139	72	348
**Chr12**	1,593	115	108	123	42	309
**Chr13**	1,209	728	225	168	60	399
**Chr14**	1,304	139	125	121	40	393
**Chr15**	666	162	101	112	50	302
**Chr16**	863	599	76	101	48	372
**Total genes**	20,626	4,516	2,490	2,185	938	6,036

Table S9. Summary numbers of genes with allele loss and no allele loss within structural variations (SVs), transposable elements (TEs) and protein–protein interactions (PPIs).

Type		Gene with allele loss	Gene with four alleles
SVs	1) gene within SVs	1,917	2,739
	2) gene within the region of SVs around 2000 bp	487	950
	Total of 1) and 2)	2,404	3,689
	3) gene within the region greater than 2000 bp	6,664	17,049
TEs	1) genes overlapping with one or multiple types of TEs	5,181	11,328
	2) genes not overlapping with one or multiple types of TEs	4,010	9,298
PPIs	1) PPI genes	5,855	17,621
	2) not PPI genes	3,336	3,005

Table S10. The results of comparing the number of genes with allele loss and with no allele loss within structural variations (SVs) by two-sided Fisher's exact test.

SVs types	Number of gene with allele loss	Number of gene with no allele loss	Proportion of gene with allele loss
inversions	413	1133	0.267
translocations	533	406	0.568
Fisher's exact test	**p-value < 2.2e-16**		
translocations	533	406	0.568
duplications	443	549	0.447
Fisher's exact test	**p-value = 1.238e-07**		

Table S12. Forty-one candidate cold-regulated genes with four or more alleles of autotetraploid *C. paliurus*.

Gene name of *C. paliurus*	Number of alleles
DCPChr01G000080	4
DCPChr01G000552	4
DCPChr01G000958	4
DCPChr01G001291	4
DCPChr01G001924	4
DCPChr02G000587	4
DCPChr02G000737	4
DCPChr02G001166	4
DCPChr02G001613	5
DCPChr04G000164	5
DCPChr05G000707	6
DCPChr05G000713	4
DCPChr05G000810	4
DCPChr07G000179	4
DCPChr07G000353	4
DCPChr07G000882	4
DCPChr07G001788	4
DCPChr07G002337	4
DCPChr08G000065	4
DCPChr08G001448	4
DCPChr09G000788	4
DCPChr10G000382	4
DCPChr10G000919	4
DCPChr10G001600	4
DCPChr11G000238	7
DCPChr11G001134	4
DCPChr12G000102	4
DCPChr12G000199	4
DCPChr12G000298	4
DCPChr12G001114	4
DCPChr12G001276	4
DCPChr12G001511	4
DCPChr12G001722	4
DCPChr13G000418	4
DCPChr13G000464	4
DCPChr14G000945	4
DCPChr14G001072	4
DCPChr15G000491	4
DCPChr16G000742	4
DCPChr16G000744	4
DCPChr16G001268	4

Table S13. Details of sample locations and descriptive statistics of genome sequencing for 118 *C. paliurus* samples.

Group	Sample	Location	Latitude (N)	Longitude (E)	Altitude (m)	Depth	Coverage
**Diploid**	Cyc-JFS96	Jinfoshan Chongqing	29.037	107.184	1940	81	0.9737
	Cyc-JFS97	Jinfoshan Chongqing	29.037	107.184	1940	79	0.9663
	Cyc-JFS98	Jinfoshan Chongqing	29.037	107.184	1940	81	0.9727
	Cyc-JZ91	Jinzhai Anhui	31.158	115.78	838	63	0.9658
	Cyc-SNJ110	Shennongjia Hubei	31.451	110.149	1803	91	0.9721
	Cyc-SNJ111	Shennongjia Hubei	31.451	110.149	1803	79	0.9748
	Cyc-SNJ17	Shennongjia Hubei	31.451	110.149	1803	77	0.9998
	Cyc-SNJ18	Shennongjia Hubei	31.451	110.149	1803	79	0.9734
	Cyc-FN100	Funing Yunnan	23.716	105.991	994	80	0.9318
	Cyc-FN101	Funing Yunnan	23.716	105.991	994	97	0.9320
	Cyc-FN102	Funing Yunnan	23.716	105.991	994	90	0.9309
	Cyc-FN99	Funing Yunnan	23.716	105.991	994	84	0.9343
**Autotetraploid**	Cyc-BTM114	Baotianman Henan	33.497	112.012	655	97	0.9705
	Cyc-BTM116	Baotianman Henan	33.497	112.012	655	113	0.9704
	Cyc-BTM117	Baotianman Henan	33.497	112.012	655	93	0.9706
	Cyc-FYS28	Fengyangshan Zhejiang	27.913	119.189	1230	69	0.9620
	Cyc-FYS29	Fengyangshan Zhejiang	27.913	119.189	1230	65	0.9644
	Cyc-FYS30	Fengyangshan Zhejiang	27.913	119.189	1230	64	0.9616
	Cyc-FYS31	Fengyangshan Zhejiang	27.913	119.189	1230	69	0.9640
	Cyc-FYS7	Fengyangshan Zhejiang	27.913	119.189	1230	82	0.9647
	Cyc-FYS79	Fengyangshan Zhejiang	27.913	119.189	1230	85	0.9657
	Cyc-FYS8	Fengyangshan Zhejiang	27.913	119.189	1230	83	0.9670
	Cyc-FYS80	Fengyangshan Zhejiang	27.913	119.189	1230	68	0.9676
	Cyc-FYS81	Fengyangshan Zhejiang	27.913	119.189	1230	84	0.9645
	Cyc-FYS82	Fengyangshan Zhejiang	27.913	119.189	1230	83	0.9652
	Cyc-HP3	Huaping Guangxi	25.641	109.898	640	67	0.9608
	Cyc-HP4	Huaping Guangxi	25.641	109.898	640	63	0.9638
	Cyc-HP51	Huaping Guangxi	25.641	109.898	640	59	0.9614

Cyc-HP52	Huaping Guangxi	25.641	109.898	640	63	0.9592
Cyc-HP53	Huaping Guangxi	25.641	109.898	640	64	0.9611
Cyc-HP54	Huaping Guangxi	25.641	109.898	640	66	0.9602
Cyc-HP55	Huaping Guangxi	25.641	109.898	640	76	0.9571
Cyc-HP56	Huaping Guangxi	25.641	109.898	640	76	0.9592
Cyc-HP58	Huaping Guangxi	25.641	109.898	640	71	0.9648
Cyc-HP59	Huaping Guangxi	25.641	109.898	640	70	0.9592
Cyc-HP60	Huaping Guangxi	25.641	109.898	640	82	0.9604
Cyc-JGS21	Jinggangshan Jiangxi	26.533	114.115	849	57	0.9594
Cyc-JGS24	Jinggangshan Jiangxi	26.533	114.115	849	58	0.9595
Cyc-JGS36	Jinggangshan Jiangxi	26.533	114.115	849	65	0.9641
Cyc-JGS37	Jinggangshan Jiangxi	26.533	114.115	849	67	0.9626
Cyc-JGS47	Jinggangshan Jiangxi	26.533	114.115	849	79	0.9626
Cyc-JGS48	Jinggangshan Jiangxi	26.533	114.115	849	73	0.9624
Cyc-JZ86	Jinzhai Anhui	31.158	115.78	838	65	0.9621
Cyc-JZ87	Jinzhai Anhui	31.158	115.78	838	56	0.9628
Cyc-JZ88	Jinzhai Anhui	31.158	115.78	838	58	0.9591
Cyc-JZ89	Jinzhai Anhui	31.158	115.78	838	72	0.9650
Cyc-JZ90	Jinzhai Anhui	31.158	115.78	838	72	0.9616
Cyc-LGS2	Leigongshan Guizhou	26.38	108.183	1463	94	0.9633
Cyc-LGS26	Leigongshan Guizhou	26.38	108.183	1463	58	0.9616
Cyc-LGS119	Leigongshan Guizhou	26.38	108.183	1463	76	0.9613
Cyc-LS15	Lushan Jiangxi	29.551	115.962	901	76	0.9646
Cyc-LS16	Lushan Jiangxi	29.551	115.962	901	74	0.9637
Cyc-LS38	Lushan Jiangxi	29.551	115.962	901	64	0.9640
Cyc-LS39	Lushan Jiangxi	29.551	115.962	901	64	0.9597
Cyc-LSX118	Leishanxian Guizhou	26.346	108.17	1482	61	0.9583
Cyc-LSX119	Leishanxian Guizhou	26.346	108.17	1482	85	0.9594
Cyc-LSX120	Leishanxian Guizhou	26.346	108.17	1482	55	0.9520
Cyc-LSX121	Leishanxian Guizhou	26.346	108.17	1482	78	0.9599
Cyc-MC19	Muchuan Sichuan	28.969	103.792	1144	73	0.9676
Cyc-MC20	Muchuan Sichuan	28.969	103.792	1144	69	0.9627

Cyc-MC65	Muchuan Sichuan	28.969	103.792	1144	73	0.9665
Cyc-MC66	Muchuan Sichuan	28.969	103.792	1144	74	0.9666
Cyc-MC67	Muchuan Sichuan	28.969	103.792	1144	66	0.9698
Cyc-MC70	Muchuan Sichuan	28.969	103.792	1144	79	0.9660
Cyc-MC71	Muchuan Sichuan	28.969	103.792	1144	50	0.9617
Cyc-MC72	Muchuan Sichuan	28.969	103.792	1144	67	0.9664
Cyc-MC73	Muchuan Sichuan	28.969	103.792	1144	79	0.9633
Cyc-MC74	Muchuan Sichuan	28.969	103.792	1144	92	0.9680
Cyc-MC75	Muchuan Sichuan	28.969	103.792	1144	85	0.9674
Cyc-NL10	Nanling Guangdong	24.922	113.077	537	84	0.9675
Cyc-NL11	Nanling Guangdong	24.922	113.077	537	69	0.9647
Cyc-NL27	Nanling Guangdong	24.922	113.077	537	61	0.9586
Cyc-NL49	Nanling Guangdong	24.922	113.077	537	75	0.9615
Cyc-NYHS12	Nanyuehengshan Hunan	27.271	112.714	384	75	0.9662
Cyc-NYHS13	Nanyuehengshan Hunan	27.271	112.714	384	88	0.9645
Cyc-NYHS35	Nanyuehengshan Hunan	27.236	112.609	441	71	0.9642
Cyc-NYHS83	Nanyuehengshan Hunan	27.236	112.609	441	88	0.9644
Cyc-QLF22	Qingliangfeng Anhui	30.149	118.894	657	58	0.9584
Cyc-QLF23	Qingliangfeng Anhui	30.149	118.894	657	61	0.9652
Cyc-QLF40	Qingliangfeng Anhui	30.149	118.894	657	59	0.9664
Cyc-QLF41	Qingliangfeng Anhui	30.149	118.894	657	64	0.9630
Cyc-QLF42	Qingliangfeng Anhui	30.149	118.894	657	55	0.9656
Cyc-QLF43	Qingliangfeng Anhui	30.149	118.894	657	57	0.9610
Cyc-QLF44	Qingliangfeng Anhui	30.149	118.894	657	69	0.9612
Cyc-QLF45	Qingliangfeng Anhui	30.149	118.894	657	77	0.9650
Cyc-QLF46	Qingliangfeng Anhui	30.149	118.894	657	67	0.9641
Cyc-SNJ112	Shennongjia Hubei	31.433	110.366	1439	69	0.9709
Cyc-TMS14	Tianmushan Zhejiang	30.327	119.44	428	97	0.9635
Cyc-TMS61	Tianmushan Zhejiang	30.327	119.44	428	87	0.9630
Cyc-TMS62	Tianmushan Zhejiang	30.327	119.44	428	78	0.9791
Cyc-TMS63	Tianmushan Zhejiang	30.327	119.44	428	62	0.9553
Cyc-TMS64	Tianmushan Zhejiang	30.327	119.44	428	64	0.9597

Cyc-TMS68	Tianmushan Zhejiang	30.327	119.44	428	67	0.9647
Cyc-TMS69	Tianmushan Zhejiang	30.327	119.44	428	83	0.9621
Cyc-TMS84	Tianmushan Zhejiang	30.327	119.44	428	66	0.9605
Cyc-TMS85	Tianmushan Zhejiang	30.327	119.44	428	77	0.9631
Cyc-TMS9	Tianmushan Zhejiang	30.327	119.44	428	62	0.9582
Cyc-TR92	Tongren Guizhou	27.546	108.787	661	88	0.9650
Cyc-TR93	Tongren Guizhou	27.546	108.787	661	77	0.9623
Cyc-WM1	Wangmo Guizhou	25.213	106.163	1315	71	0.9628
Cyc-WM25	Wangmo Guizhou	25.213	106.163	1315	59	0.9628
Cyc-WYS32	Wuyishan Fujian	27.744	117.673	740	63	0.9593
Cyc-WYS33	Wuyishan Fujian	27.744	117.673	740	70	0.9637
Cyc-WYS34	Wuyishan Fujian	27.744	117.673	740	66	0.9664
Cyc-WYS5	Wuyishan Fujian	27.744	117.673	740	63	0.9673
Cyc-WYS6	Wuyishan Fujian	27.744	117.673	740	69	0.9623
Cyc-WYS76	Wuyishan Fujian	27.744	117.673	740	85	0.9632
Cyc-WYS77	Wuyishan Fujian	27.744	117.673	740	71	0.9683
Cyc-WYS78	Wuyishan Fujian	27.744	117.673	740	80	0.9664
Cyc-YX103	Yangxian Shannxi	33.456	107.51	1384	69	0.9670
Cyc-YX104	Yangxian Shannxi	33.456	107.51	1384	69	0.9676
Cyc-YX105	Yangxian Shannxi	33.456	107.51	1384	58	0.9637
Cyc-YX106	Yangxian Shannxi	33.456	107.51	1384	59	0.9661
Cyc-YX107	Yangxian Shannxi	33.456	107.51	1384	68	0.9697
Cyc-YX108	Yangxian Shannxi	33.456	107.51	1384	76	0.9694
Cyc-YX109	Yangxian Shannxi	33.456	107.51	1384	68	0.9698
Cyc-ZJJ94	Zhangjiajie Hunan	29.351	110.483	439	76	0.9628
Cyc-ZJJ95	Zhangjiajie Hunan	29.351	110.483	439	61	0.9646

Table S14. The information of ploidy estimation of 118 samples of *C. paliurus* by using nQuire.

**Sample**	**Ploidy**	**$\Delta \log L_{2n}$**	**$\Delta \log L_{3n}$**	**$\Delta \log L_{4n}$**
Cyc-FN100	2n	**847,167**	1,817,276	1,106,045
Cyc-FN101	2n	**1,046,079**	2,168,562	1,254,775
Cyc-FN102	2n	**984,370**	2,097,668	1,227,481
Cyc-FN99	2n	**930,411**	2,352,078	1,389,435
Cyc-JFS96	2n	**146,631**	5,481,388	3,495,537
Cyc-JFS97	2n	**207,151**	4,445,903	2,854,470
Cyc-JFS98	2n	**162,340**	5,514,211	3,512,184
Cyc-JZ91	2n	**179,342**	4,421,452	2,967,674
Cyc-SNJ110	2n	**239,228**	5,157,580	3,196,105
Cyc-SNJ111	2n	**195,893**	5,620,619	3,559,369
Cyc-SNJ17	2n	**22,185**	5,770,657	3,785,044
Cyc-SNJ18	2n	**198,252**	5,579,618	3,592,757
Cyc-BTM114	4n	15,309,331	10,572,087	**2,258,531**
Cyc-BTM116	4n	16,231,757	11,447,228	**2,143,454**
Cyc-BTM117	4n	15,218,779	10,403,801	**1,640,273**
Cyc-FYS28	4n	11,240,601	7,537,148	**1,042,513**
Cyc-FYS29	4n	13,504,960	8,668,001	**1,394,473**
Cyc-FYS30	4n	10,921,557	7,108,806	**1,026,015**
Cyc-FYS31	4n	13,363,144	8,732,719	**1,326,476**
Cyc-FYS79	4n	13,080,787	8,868,836	**1,312,326**
Cyc-FYS7	4n	14,225,178	9,511,435	**1,440,875**
Cyc-FYS80	4n	14,324,078	9,578,161	**1,449,625**
Cyc-FYS81	4n	11,581,982	7,690,268	**1,074,009**
Cyc-FYS82	4n	12,873,554	8,871,565	**1,693,050**
Cyc-FYS8	4n	14,505,235	9,728,941	**1,500,164**
Cyc-HP3	4n	11,044,484	7,331,887	**1,020,714**
Cyc-HP4	4n	12,393,694	7,966,755	**1,237,161**
Cyc-HP51	4n	12,876,713	8,153,909	**1,433,237**
Cyc-HP52	4n	10,418,879	6,854,929	**946,681**
Cyc-HP53	4n	12,666,785	8,127,448	**1,260,117**
Cyc-HP54	4n	10,887,121	7,201,852	**1,009,953**
Cyc-HP55	4n	11,393,819	7,821,719	**1,116,949**
Cyc-HP56	4n	12,141,763	8,280,854	**1,192,163**
Cyc-HP58	4n	14,041,012	9,136,504	**1,441,488**
Cyc-HP59	4n	12,880,698	8,471,653	**1,232,353**
Cyc-HP60	4n	13,065,280	8,938,836	**1,331,650**
Cyc-JGS21	4n	12,105,166	7,683,197	**1,367,361**
Cyc-JGS24	4n	12,639,274	7,997,506	**1,447,689**

Cyc-JGS36	4n	12,664,369	8,123,751	**1,258,133**
Cyc-JGS37	4n	13,101,898	8,497,690	**1,314,639**
Cyc-JGS47	4n	13,977,321	9,394,143	**1,418,669**
Cyc-JGS48	4n	13,843,468	9,097,557	**1,384,932**
Cyc-JZ86	4n	13,303,329	8,569,769	**1,351,551**
Cyc-JZ87	4n	12,537,141	7,933,448	**1,403,713**
Cyc-JZ88	4n	12,451,309	7,864,257	**1,391,544**
Cyc-JZ89	4n	13,787,509	9,067,818	**1,370,495**
Cyc-JZ90	4n	12,176,527	8,068,797	**1,143,853**
Cyc-LGS119	4n	13,224,462	9,430,775	**1,396,363**
Cyc-LGS2	4n	13,942,201	9,254,756	**1,394,636**
Cyc-LGS26	4n	13,103,265	8,317,822	**1,452,525**
Cyc-LS15	4n	13,552,113	9,031,219	**1,345,428**
Cyc-LS16	4n	13,736,021	9,040,744	**1,374,310**
Cyc-LS38	4n	13,139,230	8,450,763	**1,308,117**
Cyc-LS39	4n	10,273,133	6,823,177	**950,105**
Cyc-LSX118	4n	11,436,602	7,339,352	**1,078,110**
Cyc-LSX119	4n	14,498,006	9,756,871	**1,477,464**
Cyc-LSX120	4n	11,190,671	7,125,232	**1,141,730**
Cyc-LSX121	4n	12,501,936	8,357,456	**1,198,725**
Cyc-MC19	4n	13,919,080	9,148,020	**1,385,688**
Cyc-MC20	4n	13,688,545	8,947,343	**1,369,968**
Cyc-MC65	4n	13,896,937	9,143,897	**1,409,685**
Cyc-MC66	4n	13,643,081	9,024,171	**1,362,762**
Cyc-MC67	4n	13,298,137	8,538,003	**1,408,364**
Cyc-MC70	4n	13,866,288	9,213,765	**1,397,055**
Cyc-MC71	4n	10,999,765	6,779,268	**1,173,261**
Cyc-MC72	4n	13,485,682	8,734,579	**1,359,701**
Cyc-MC73	4n	13,590,462	9,147,390	**1,380,263**
Cyc-MC74	4n	14,734,431	10,002,206	**1,574,223**
Cyc-MC75	4n	13,283,366	9,089,486	**1,363,051**
Cyc-NL10	4n	14,017,825	9,418,639	**1,423,055**
Cyc-NL11	4n	13,394,205	8,697,437	**1,313,154**
Cyc-NL27	4n	12,767,646	8,128,200	**1,423,842**
Cyc-NL49	4n	13,871,059	9,127,086	**1,382,530**
Cyc-NYHS12	4n	13,182,504	8,753,435	**1,307,830**
Cyc-NYHS13	4n	14,226,069	9,730,813	**1,488,604**
Cyc-NYHS35	4n	13,476,607	8,835,806	**1,338,912**
Cyc-NYHS83	4n	14,510,425	9,791,582	**1,493,498**
Cyc-QLF22	4n	13,036,732	8,276,492	**1,454,294**
Cyc-QLF23	4n	13,262,796	8,476,091	**1,385,982**
Cyc-QLF40	4n	12,624,630	7,997,154	**1,388,595**
Cyc-QLF41	4n	13,213,943	8,438,141	**1,414,740**
Cyc-QLF42	4n	12,408,610	7,812,046	**1,426,003**
Cyc-QLF43	4n	12,799,022	8,085,444	**1,445,460**
Cyc-QLF44	4n	13,740,932	8,985,339	**1,393,957**

Cyc-QLF45	4n	14,532,242	9,619,302	**1,489,759**
Cyc-QLF46	4n	13,576,388	8,808,772	**1,404,635**
Cyc-SNJ112	4n	8,075,991	5,625,166	**1,121,223**
Cyc-TMS14	4n	15,121,181	10,326,626	**1,621,319**
Cyc-TMS61	4n	13,506,194	9,205,924	**1,389,886**
Cyc-TMS62	4n	9,033,150	6,310,285	**1,278,694**
Cyc-TMS63	4n	12,254,825	7,886,543	**1,235,982**
Cyc-TMS64	4n	12,905,438	8,277,551	**1,314,602**
Cyc-TMS68	4n	12,821,692	8,401,906	**1,264,588**
Cyc-TMS69	4n	14,372,256	9,612,813	**1,488,857**
Cyc-TMS84	4n	13,210,407	8,554,926	**1,362,481**
Cyc-TMS85	4n	13,256,656	8,761,784	**1,314,692**
Cyc-TMS9	4n	12,973,530	8,294,127	**1,366,552**
Cyc-TR92	4n	15,114,990	10,243,391	**1,594,750**
Cyc-TR93	4n	14,273,168	9,415,304	**1,440,304**
Cyc-WM1	4n	13,706,907	8,930,649	**1,392,222**
Cyc-WM25	4n	13,005,239	8,260,447	**1,473,792**
Cyc-WYS32	4n	12,777,408	8,217,717	**1,334,323**
Cyc-WYS33	4n	13,243,112	8,635,485	**1,308,620**
Cyc-WYS34	4n	13,169,951	8,363,586	**1,408,911**
Cyc-WYS5	4n	12,877,403	8,208,754	**1,338,238**
Cyc-WYS6	4n	13,616,125	8,871,554	**1,371,924**
Cyc-WYS76	4n	14,495,300	9,769,574	**1,483,618**
Cyc-WYS77	4n	14,067,384	9,134,629	**1,452,675**
Cyc-WYS78	4n	13,865,393	9,233,128	**1,359,113**
Cyc-YX103	4n	13,473,430	8,799,261	**1,347,741**
Cyc-YX104	4n	13,753,066	8,990,659	**1,380,084**
Cyc-YX105	4n	13,118,296	8,326,509	**1,498,410**
Cyc-YX106	4n	13,302,634	8,445,158	**1,469,997**
Cyc-YX107	4n	13,901,310	9,042,333	**1,417,953**
Cyc-YX108	4n	14,401,312	9,591,746	**1,457,326**
Cyc-YX109	4n	13,420,146	8,764,904	**1,315,255**
Cyc-ZJJ94	4n	13,670,760	9,067,044	**1,347,932**
Cyc-ZJJ95	4n	13,144,633	8,368,269	**1,412,905**

The smallest $\Delta \log L$ for each sample is highlighted in bold.

Table S15. The statistical analysis between the observed and simulated SFS distribution using Kolmogorov-Smirnov test.

Group	*P* value
tetrasomic inheritance mode	0.005
disomic inheritance mode	0.002
10% disomic inheritance mode	**0.227**
20% disomic inheritance mode	**0.132**
30% disomic inheritance mode	**0.132**
40% disomic inheritance mode	0.034
50% disomic inheritance mode	0.015
60% disomic inheritance mode	0.015
70% disomic inheritance mode	0.015
80% disomic inheritance mode	0.006
90% disomic inheritance mode	0.006

Table S16. The information of eight genes under positive selection that were significantly enriched in the terms response to biotic stimulus in autotetraploid *C. paliurus*.

**Gene ID**	**Chromosome**	**Gene start**	**Gene end**	**DCMS**	***p*-value**	**Function**
DCPChr13G001680	13	32142414	32143544	1.789	0.037	MLP-like protein 43, function as a positive regulator during abscisic acid responses and confers drought tolerance
DCPChr13G001681	13	32171724	32172826	3.203	0.001	MLP-like protein 43, function as a positive regulator during abscisic acid responses and confers drought tolerance
DCPChr13G001683	13	32251598	32256970	2.253	0.012	MLP-like protein 328, response to cis-cinnamic acid that functions in regulation of bolting in *Arabidopsis*
DCPChr13G001684	13	32291269	32291481	2.130	0.017	MLP-like protein 31, proteins are associated with the effects of salicylic acid on *Arabidopsis* seed germination and the establishment of early defense mechanisms.
DCPChr13G001685	13	32308245	32312287	1.713	0.043	ARF-GAP domain proteins, response to auxin stimulus
DCPChr13G001686	13	32321633	32325058	1.713	0.043	MLP-like protein 31, proteins are associated with the effects of salicylic acid on Arabidopsis seed germination and the establishment of early defense mechanisms.
DCPChr16G000507	16	15989588	15990308	1.904	0.028	MLP-like protein 328, response to cis-cinnamic acid that functions in regulation of bolting in *Arabidopsis*
DCPChr16G000508	16	15990534	15990713	1.904	0.028	MLP-like protein 328, response to cis-cinnamic acid that functions in regulation of bolting in *Arabidopsis*

Table S17. We have identified 62 homologous meiosis-related genes in *C. paliurus*.

C. paliurus gene name	ATG ID
DCPChr02G000276	AT1G01690
DCPChr01G001704	AT1G06660
DCPChr11G000115	AT1G08880
DCPChr13G001577	AT1G10710
DCPChr13G000416	AT1G10930
DCPChr08G000427	AT1G22260
DCPChr13G000970	AT1G27900
DCPChr15G000539	AT1G34355
DCPChr01G000180	AT1G35530
DCPChr09G000967	AT1G53490
DCPChr02G000932	AT1G63990
DCPChr01G000686	AT1G66170
DCPChr13G000940	AT1G67370
DCPChr15G000220	AT1G77320
DCPChr13G001911	AT1G77600
DCPChr14G001105	AT2G27170
DCPChr07G001050	AT2G31970
DCPChr07G002390	AT2G45280
DCPChr08G000758	AT2G46980
DCPChr11G000911	AT2G47980
DCPChr10G000066	AT3G02680
DCPChr11G000199	AT3G05480
DCPChr02G001789	AT3G13170
DCPChr06G000882	AT3G18524
DCPChr13G000060	AT3G19210
DCPChr02G001707	AT3G20475
DCPChr12G001082	AT3G22880
DCPChr12G000213	AT3G25100
DCPChr01G001782	AT3G43210
DCPChr02G000234	AT3G48190
DCPChr14G000031	AT3G52115
DCPChr01G002365	AT3G54670
DCPChr04G001202	AT3G57300
DCPChr11G001092	AT4G02070
DCPChr11G002215	AT4G09140
DCPChr07G001431	AT4G14220
DCPChr02G000664	AT4G17380

DCPChr07G001038	AT4G20900
DCPChr04G000925	AT4G21270
DCPChr13G000371	AT4G22970
DCPChr01G000954	AT4G25540
DCPChr10G001386	AT4G29170
DCPChr14G000097	AT4G30870
DCPChr03G001919	AT5G01630
DCPChr07G002748	AT5G05490
DCPChr01G000808	AT5G15540
DCPChr05G000050	AT5G15920
DCPChr11G000793	AT5G16270
DCPChr14G001343	AT5G19400
DCPChr14G001112	AT5G22000
DCPChr16G000389	AT5G24280
DCPChr16G001057	AT5G40820
DCPChr03G001008	AT5G47690
DCPChr14G000527	AT5G48390
DCPChr10G000934	AT5G48720
DCPChr01G000846	AT5G51330
DCPChr10G000670	AT5G52290
DCPChr02G001577	AT5G54260
DCPChr08G000348	AT5G57450
DCPChr01G000194	AT5G61460
DCPChr10G000735	AT5G63920
DCPChr11G000552	AT5G66130

Table S18. The information of eight meiosis-related genes in diploid and autotetraploid of *C. paliurus*.

***C. paliurus* Gene ID**	**ATG ID**	**Name**	**Description**
DCPChr01G001704	AT1G06660	*JASON*	Encodes JASON. *jason* mutant produces diploid male gametes leading to triploid progeny. Diploid gametes in the *jason* mutant are generated by a defect in male meiosis II.
DCPChr02G000234	AT3G48190	*ATM*	homolog of human ataxia telangiectasia mutated
DCPChr02G001789	AT3G13170	*SPO11*-1	Required for meiotic recombination
DCPChr08G000427	AT1G22260	*ZYP1a*	SC transverse filament protein
DCPChr08G000758	AT2G46980	*ASY3*	Encodes ASY3, a coiled-coil domain protein that is required for normal meiosis
DCPChr11G000911	AT2G47980	*SCC3*	Essential to the monopolar orientation of the kinetochores during meiosis.
DCPChr11G002215	AT4G09140	*MLH1*	Encodes a protein with similarity to *Mut1* DNA mismatch repair protein, from *E. coli*. The protein is expressed during prophase I of meiosis,
DCPChr16G001057	AT5G40820	*ATRAD3*	Encodes an Arabidopsis ortholog of the ATR protein kinase that is involved in a wide range of responses to DNA damage and plays a central role in cell-cycle regulation.

Table S19. A summary of the number of SNPs remaining after several filtering steps.

Filtering steps	**118 individuals**
Original SNP number	132,960,641
1) Filtering missing SNPs;<br>2) SNPs whose depth does not meet the average depth of 1/3 to 2;<br>3) SNPs with more than 4 alleles	45,540,703
4) Using a distance filter of 20 kb based on LD results, selecting independent SNPs	24,657<br>4,657
5) Filtering SNPs located in CDS and its 20-kb extension region	6,264
6) Filtering biallelic SNPs of diploid samples	6,103
7) Filtering SNPs with minor allele frequencies <0.01	2,849

Table S20. The mapping information of three autotetraploid samples for allele expression analysis.

Reads	Cyc-1A		Cyc-2A		Cyc-3A	
	No. of pairs	Ratio (%)	No. of pairs	Ratio (%)	No. of pairs	Ratio (%)
Total paired reads	21,066,117	1.00	23,892,234	1.00	23,871,603	1.000
Unique aligned pairs	6,993,973	0.33	7,815,569	0.33	8,126,687	0.340
Multiple aligned pairs	12,496,038	0.59	1,4221,370	0.60	13,957,224	0.585
Not aligned pairs	1,576,106	0.07	1,855,295	0.078	1,787,692	0.075
Total aligned pairs	19,490,011	0.92	22,036,939	0.9223	22,083,911	0.9251

Table S21. Sample details of RNA-seq analysis in diploid and tetraploid *C. paliurus.*

Sample id	Ploidy	Batches	Tissue
Cyc_L1_1	diploid	1	leaf
Cyc_L1_2	diploid	1	leaf
Cyc_L2_1	diploid	1	leaf
Cyc_L2_2	diploid	1	leaf
Cyc_L3_1	diploid	1	leaf
Cyc_L3_2	diploid	1	leaf
Cyc_L4_1	diploid	1	leaf
Cyc_L4_2	diploid	1	leaf
Cyc-D_L1	diploid	2	leaf
Cyc-D_L2	diploid	2	leaf
Cyc-D_L3	diploid	2	leaf
Cwm_L1_1	tetraploid	3	leaf
Cwm_L1_2	tetraploid	3	leaf
Cwm_L1_3	tetraploid	3	leaf
Cwm_L2_1	tetraploid	3	leaf
Cwm_L2_2	tetraploid	3	leaf
Cwm_L2_3	tetraploid	3	leaf
Cyc-1A	tetraploid	4	leaf
Cyc-2A	tetraploid	4	leaf
Cyc-3A	tetraploid	4	leaf
