# Supplementary material for: Genomic insights into biased allele loss and increased gene numbers after genome duplication in autotetraploid Cyclocarya paliurus
Source: BMC Biol. 2023 Aug 8;21:168. doi: 10.1186/s12915-023-01668-1 (PMC10408227; doi:10.1186/s12915-023-01668-1)

Genomic insights into biased allele loss and increased gene numbers after genome duplication in autotetraploid *Cyclocarya paliurus*

Rui-Min Yu[1], Ning Zhang[1], Bo-Wen Zhang[1], Yu Liang[1], Xiao-Xu Pang[1], Lei Cao[1], Yi-Dan Chen[1], Wei-Ping Zhang[1], Yang Yang[1], Da-Yong Zhang[1]*, Er-Li Pang[1]*, & Wei-Ning Bai[1]*

[1]State Key Laboratory of Earth Surface Processes and Resource Ecology, and Ministry of Education Key Laboratory for Biodiversity Science and Ecological Engineering, College of Life Sciences, Beijing Normal University, Beijing 100875, China.

*Corresponding authors: Da-Yong Zhang (zhangdy@bnu.edu.cn), Er-Li Pang (pangerli@bnu.edu.cn), Wei-Ning Bai (baiwn@bnu.edu.cn).

Here, we showcase 10 instances of copy gain and 10 instances of copy loss in the autotetraploid *C. paliurus*.

Firstly, we showed 10 examples of copy gain.

Example 1:
The locus has five copies: Cpa02G095910, Cpa02G094840, **Cpa02G095690**, Cpa02G092590, and Cpa02G091740. In addition to the four original copies (not bolded), an additional copy was formed by duplication (bolded). In the following figure, the four original copies and the duplicated copy (highlighted with a red box) are all covered by HiFi reads.

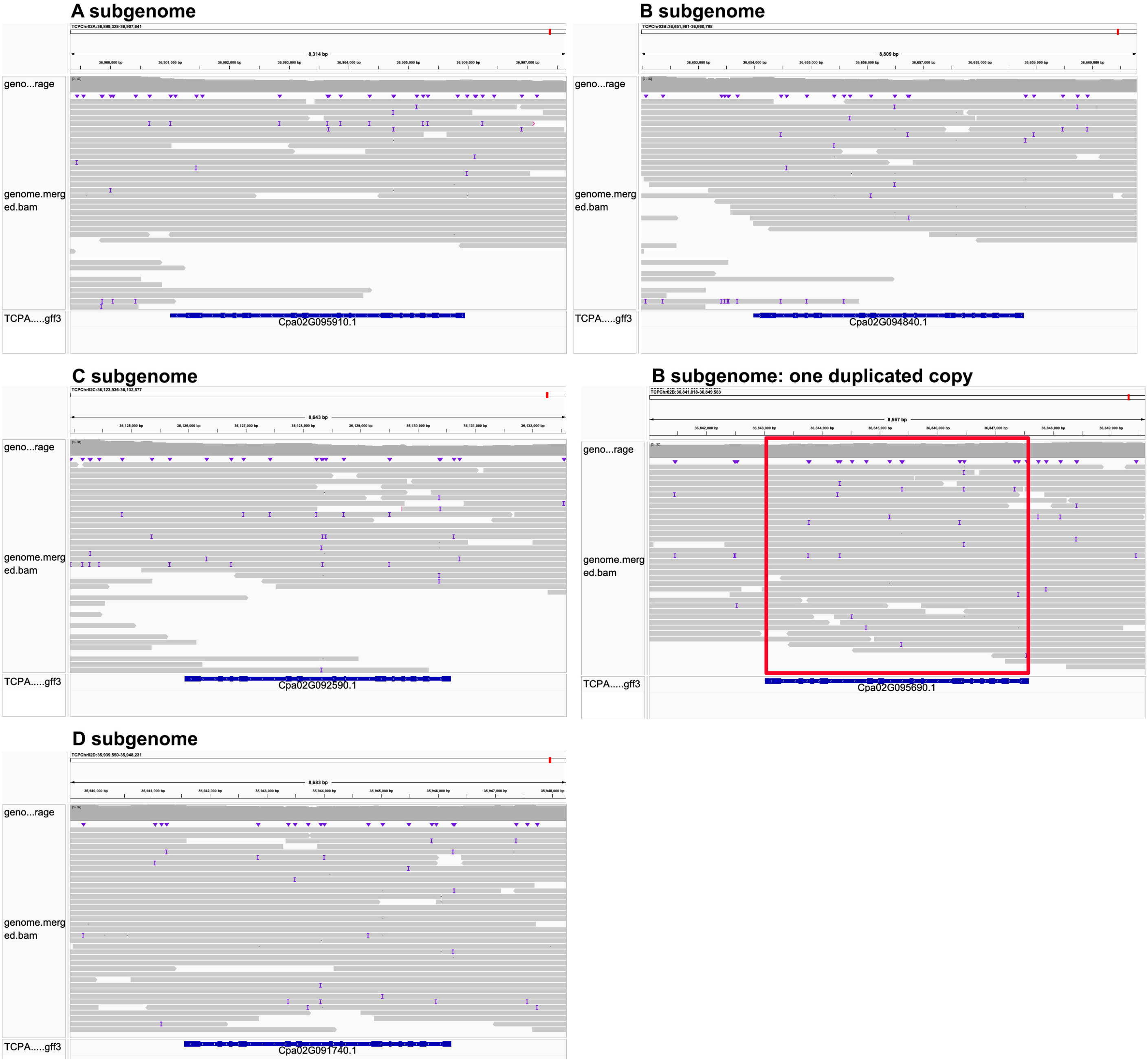

Example 2:

The locus has five copies: Cpa03G009320, Cpa03G009240, **Cpa03G009330**, Cpa03G009270, and Cpa03G009100. In addition to the four original copies (not bolded), an additional copy was formed by duplication (bolded). In the following figure, the four original copies and the duplicated copy (highlighted with a red box) are all covered by HiFi reads.

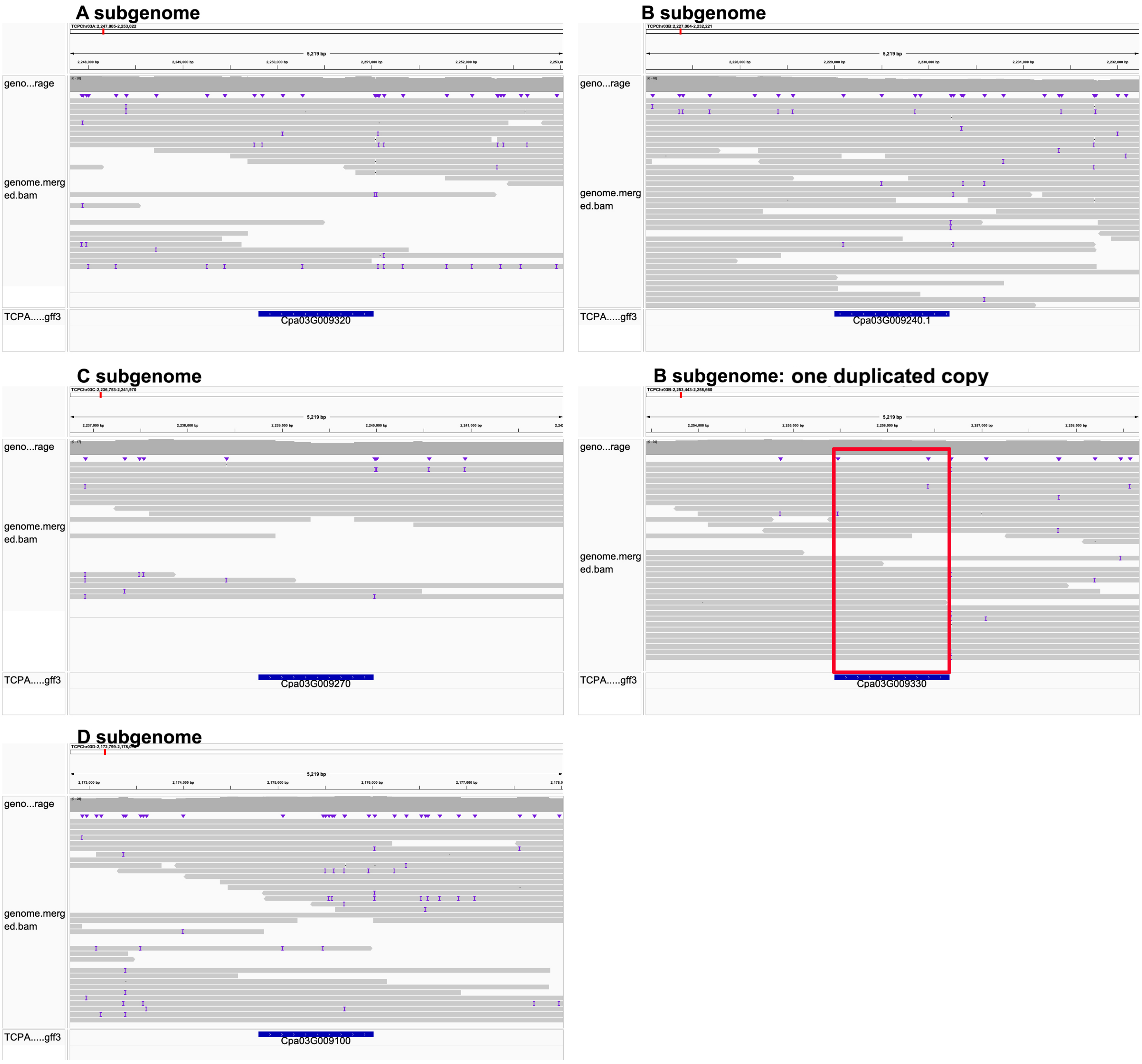

Example 3:

The locus has five copies: Cpa04G023780, Cpa04G021850, Cpa04G023240, **Cpa04G023290** and Cpa04G023870. In addition to the four original copies (not bolded), an additional copy was formed by duplication (bolded). In the following figure, the four original copies and the duplicated copy (highlighted with a red box) are all covered by HiFi reads.

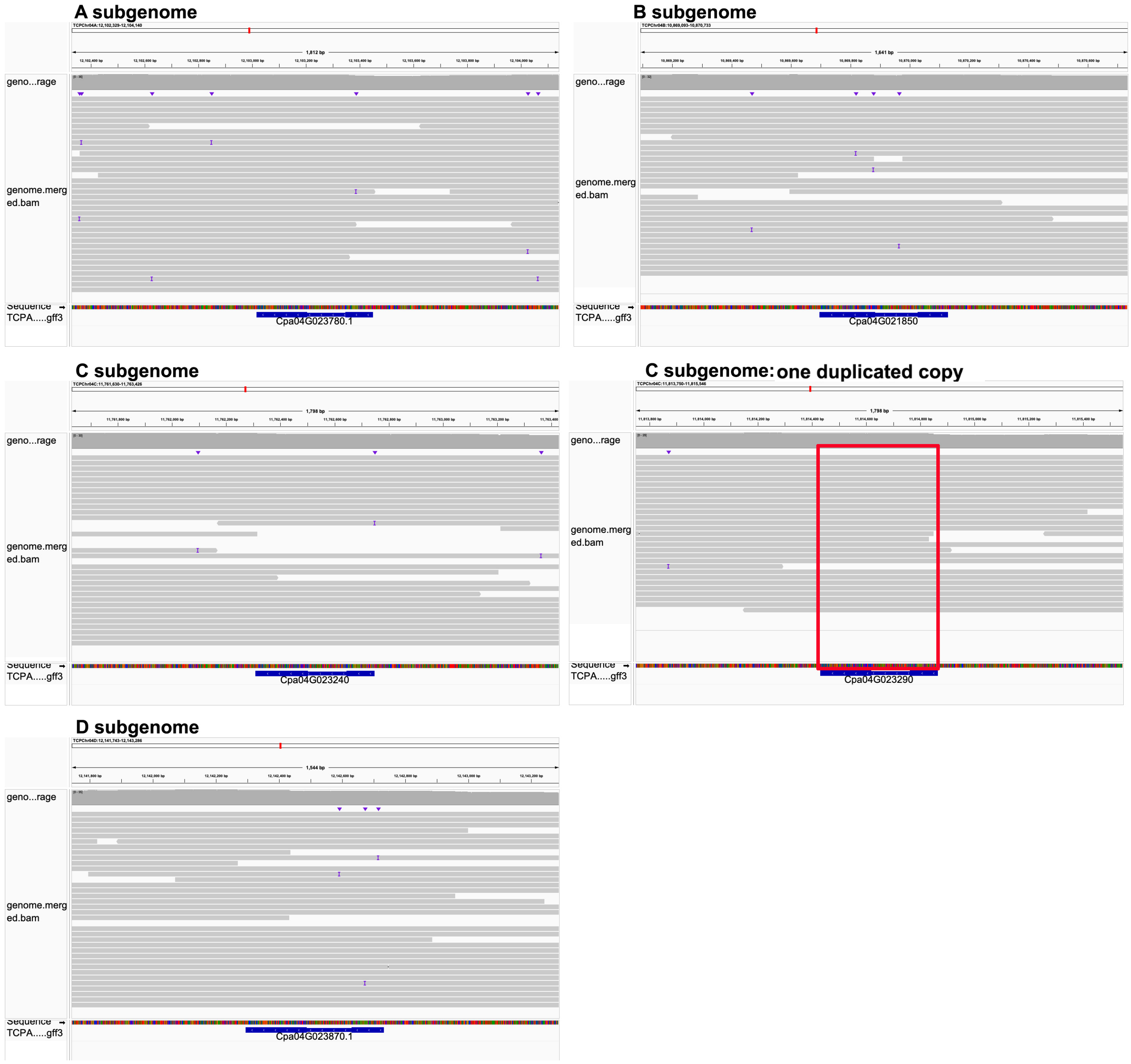

Example 4:

The locus has five copies: Cpa06G024830, Cpa06G025220, Cpa06G024030, Cpa06G024130 and **Cpa06G024460**. In addition to the four original copies (not bolded), an additional copy was formed by duplication (bolded). In the following figure, the four original copies and the duplicated copy (highlighted with a red box) are all covered by HiFi reads.

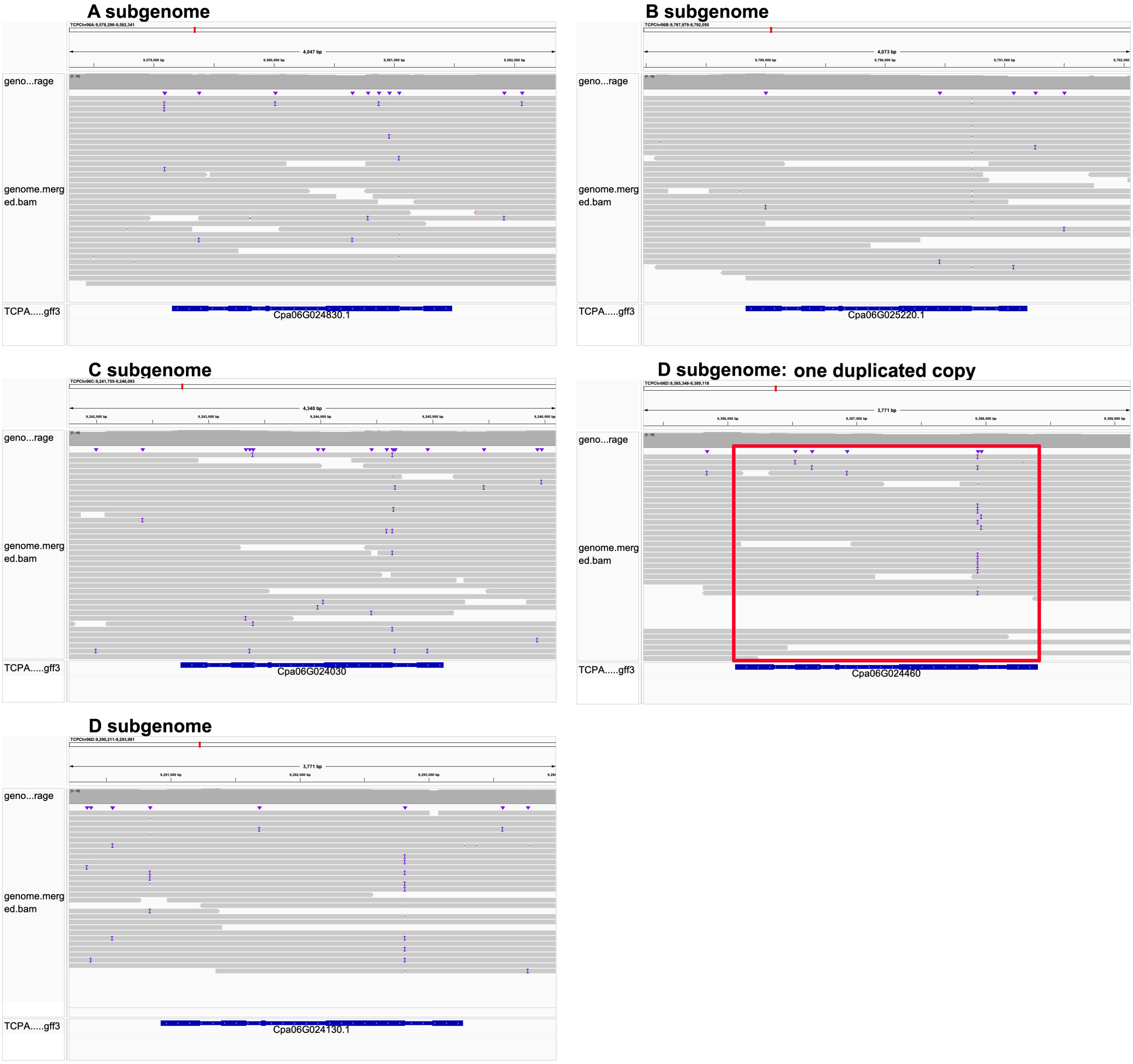

Example 5:

The locus has five copies: Cpa06G031920, Cpa06G031770, Cpa06G031150, **Cpa06G031170** and Cpa06G032080. In addition to the four original copies (not bolded), an additional copy was formed by duplication (bolded). In the following figure, the four original copies and the duplicated copy (highlighted with a red box) are all covered by HiFi reads.

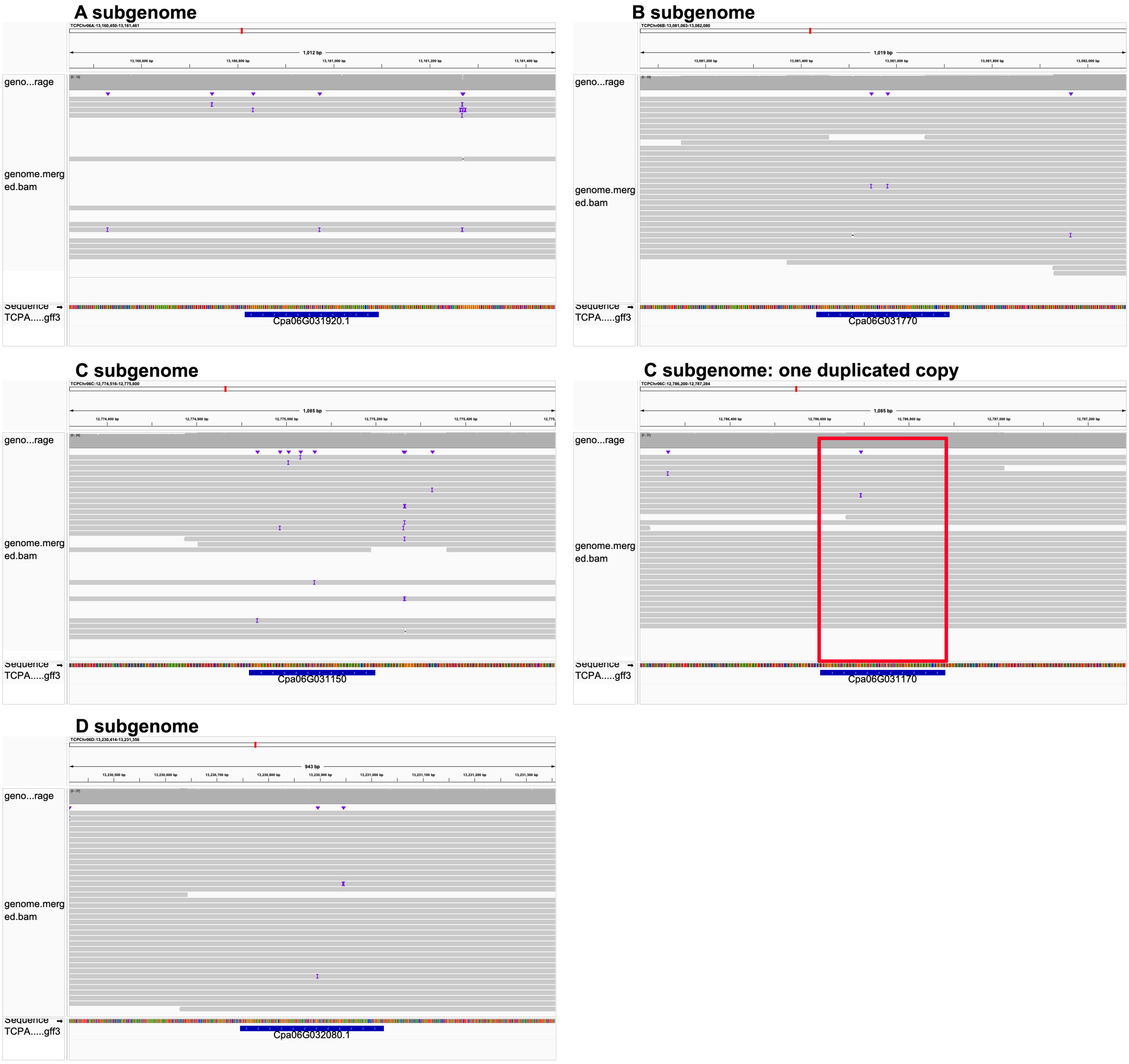

Example 6:

The locus has five copies: Cpa10G013340, **Cpa10G013440**, Cpa10G013400, Cpa10G013430 and Cpa10G013670. In addition to the four original copies (not bolded), an additional copy was formed by duplication (bolded). In the following figure, the four original copies and the duplicated copy (highlighted with a red box) are all covered by HiFi reads.

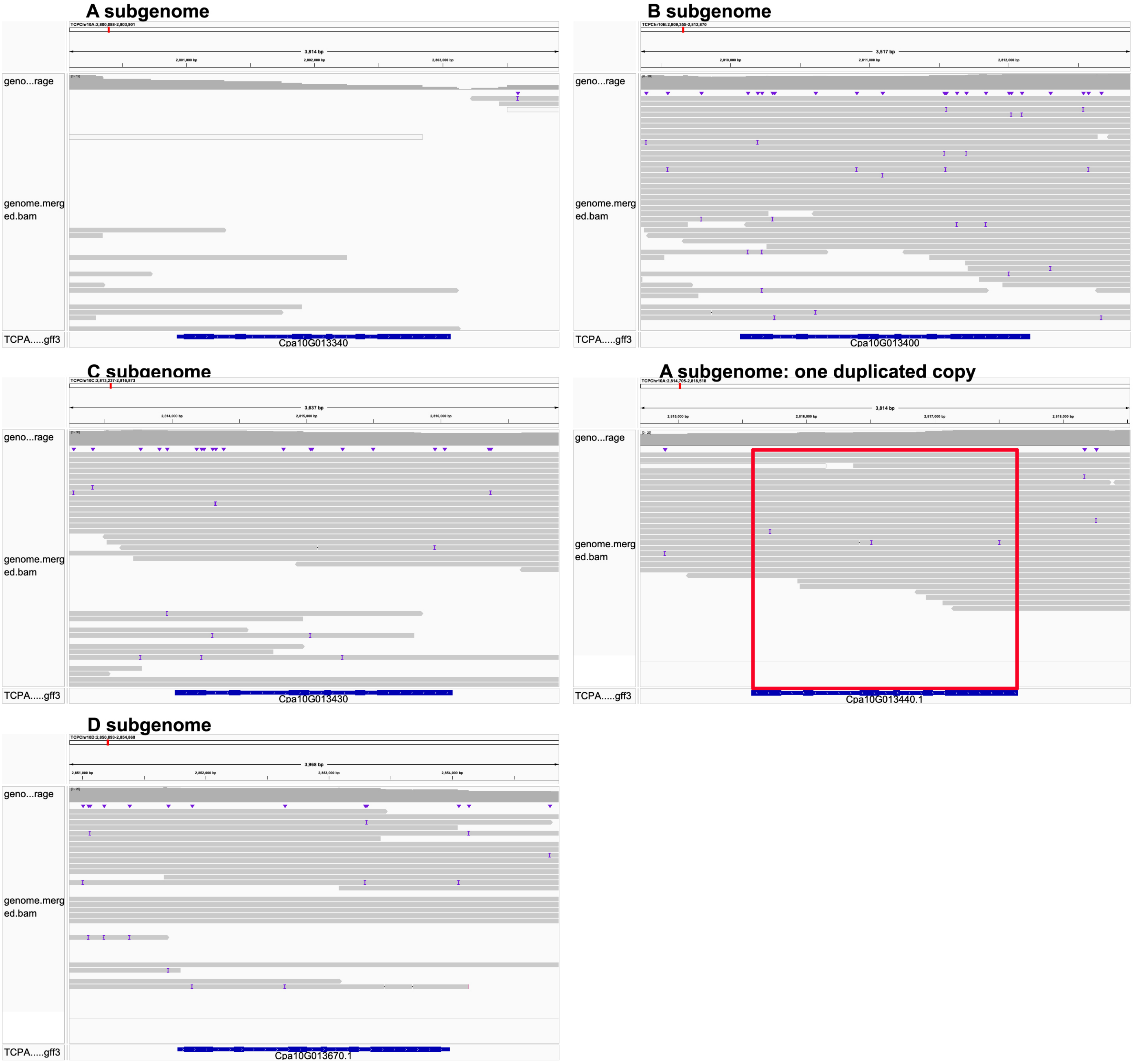

Example 7:

The locus has five copies: Cpa10G055550, Cpa10G055980, **Cpa10G056000**, Cpa10G056650 and Cpa10G055810. In addition to the four original copies (not bolded), an additional copy was formed by duplication (bolded). In the following figure, the four original copies and the duplicated copy (highlighted with a red box) are all covered by HiFi reads.

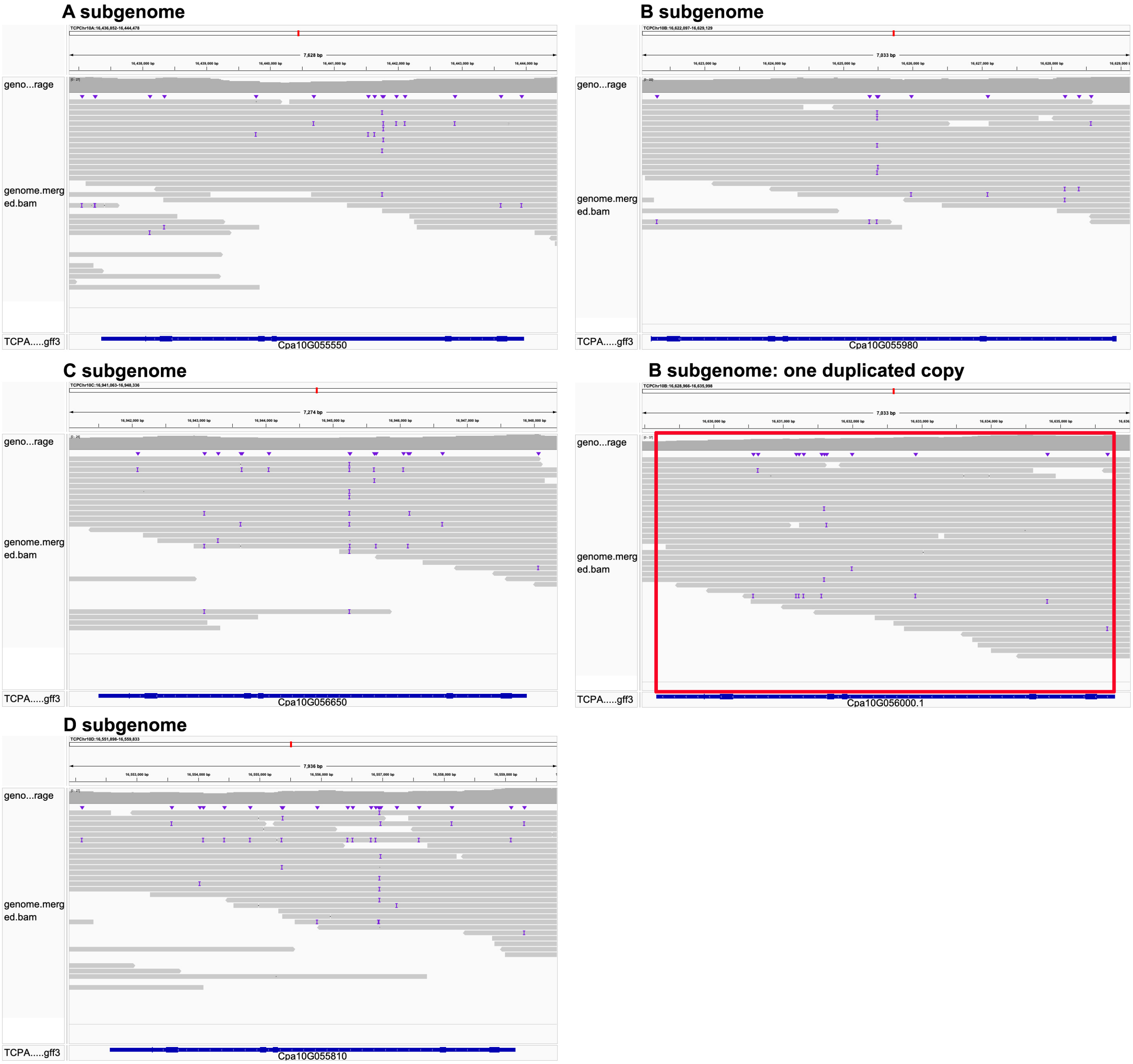

Example 8:

The locus has five copies: Cpa11G002670, Cpa11G002910, Cpa11G002900, **Cpa11G002950** and Cpa11G002770. In addition to the four original copies (not bolded), an additional copy was formed by duplication (bolded). In the following figure, the four original copies and the duplicated copy (highlighted with a red box) are all covered by HiFi reads.

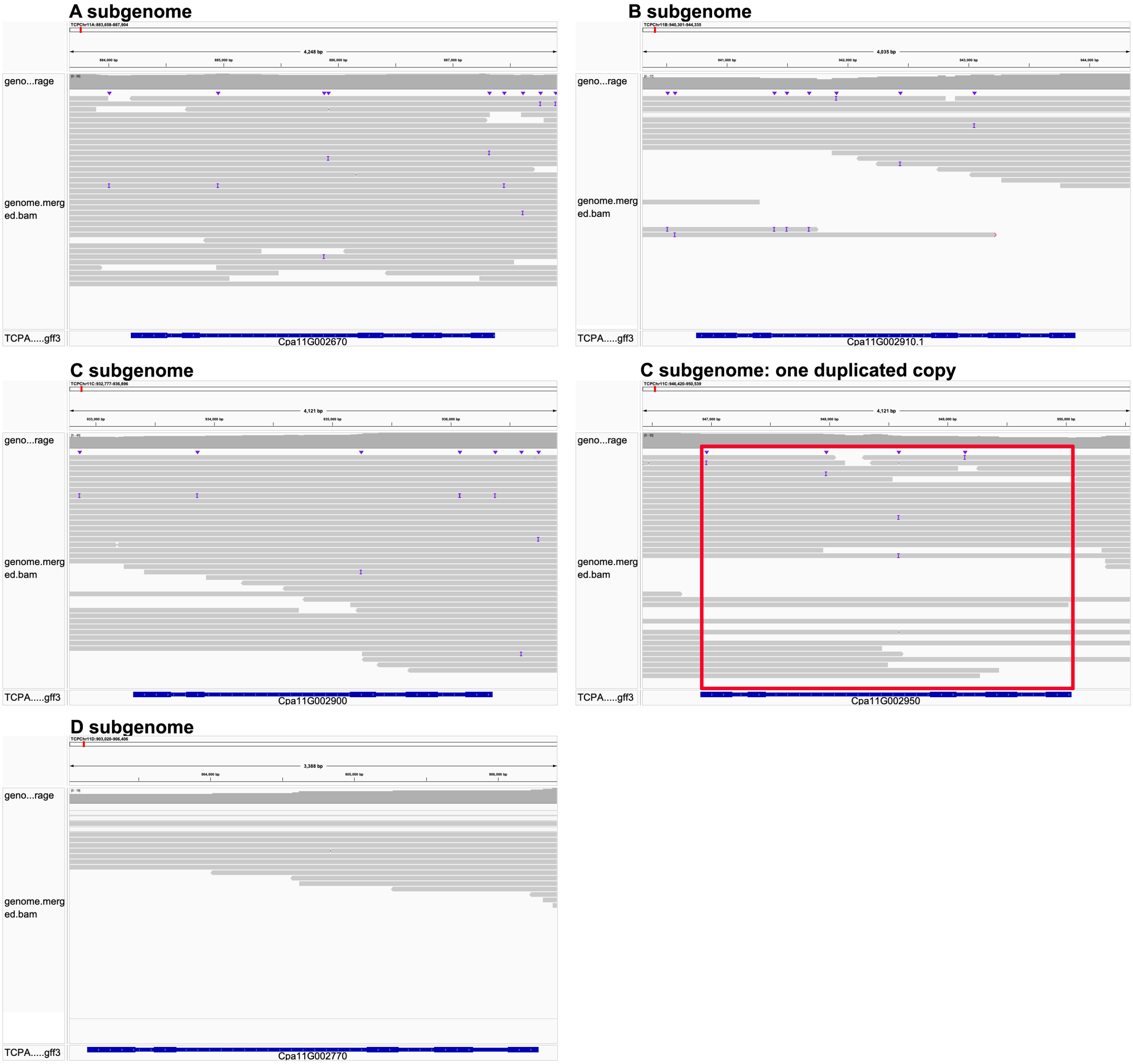

Example 9:

The locus has five copies: Cpa14G035760, Cpa14G032810, **Cpa14G033130**, Cpa14G031500 and Cpa14G032750. In addition to the four original copies (not bolded), an additional copy was formed by duplication (bolded). In the following figure, the four original copies and the duplicated copy (highlighted with a red box) are all covered by HiFi reads.

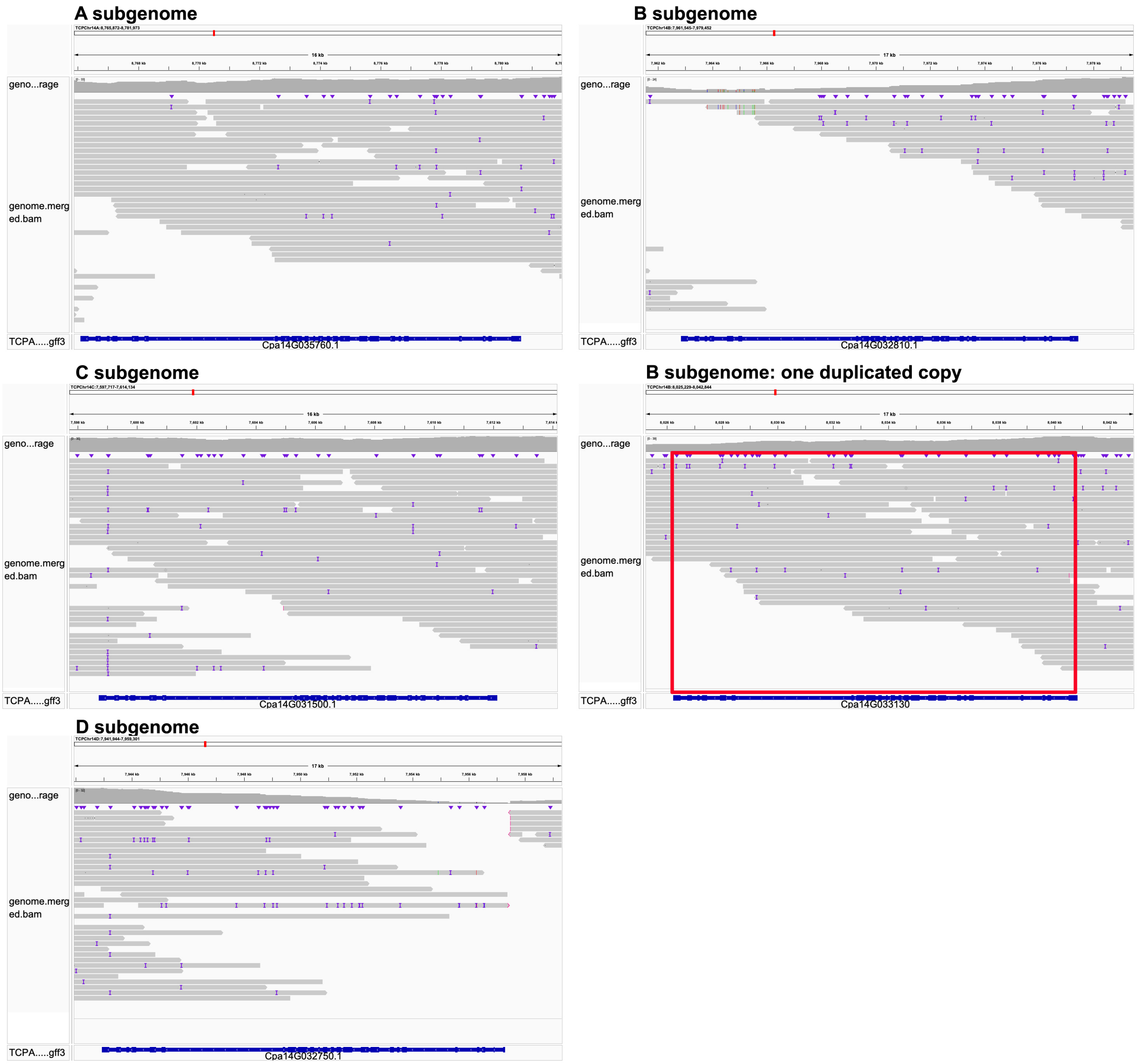

Example 10:

The locus has five copies: Cpa15G003140, Cpa15G003210, Cpa15G002890, **Cpa15G002960** and Cpa15G003280. In addition to the four original copies (not bolded), an additional copy was formed by duplication (bolded). In the following figure, the four original copies and the duplicated copy (highlighted with a red box) are all covered by HiFi reads.

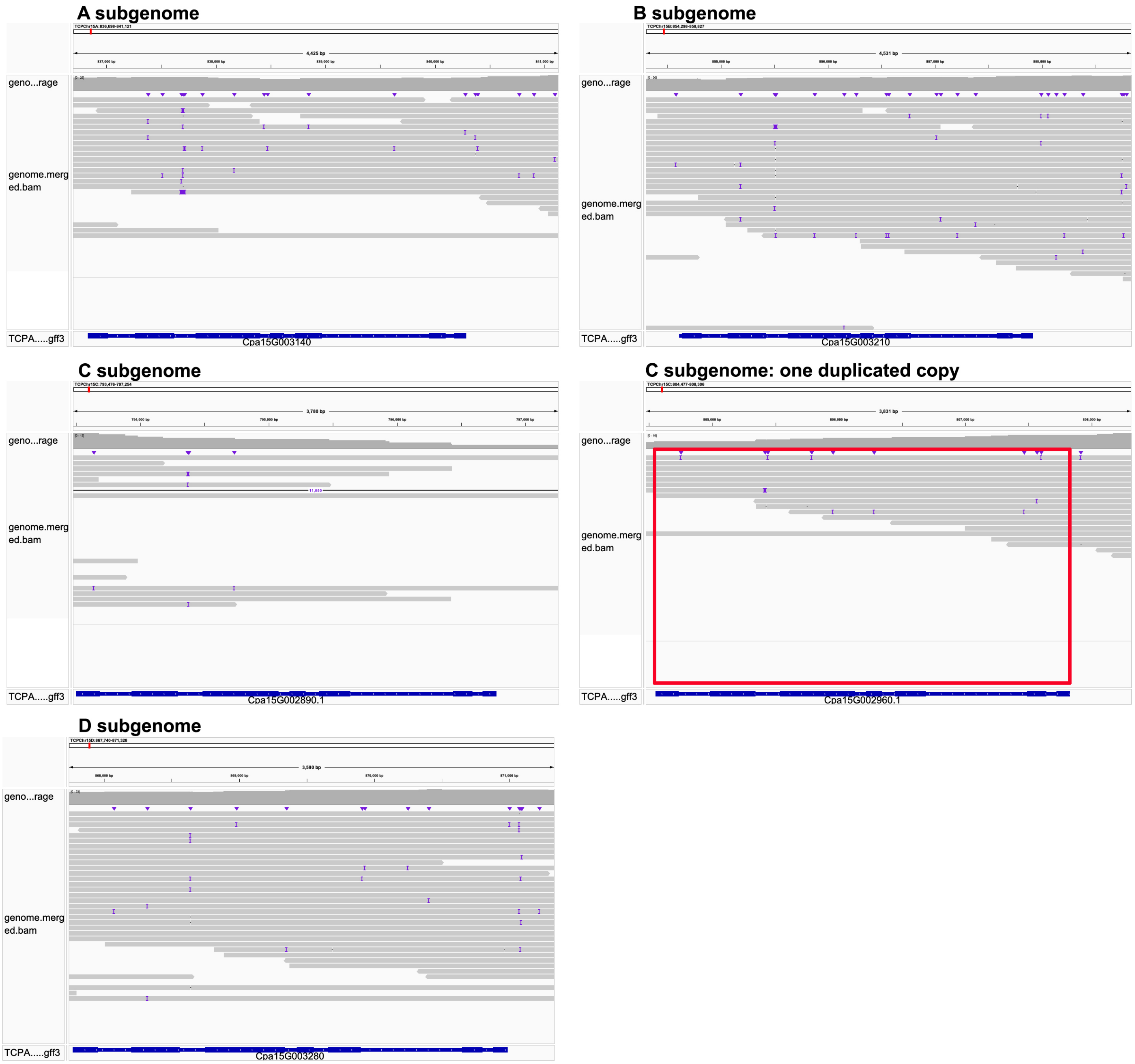

This part showed 10 examples of copy loss.

Example 1:

The locus lost one copy and now has three remaining copies: Cpa08G069140, Cpa08G061320 and Cpa08G068360. In the following figure, the HiFi reads cover the three remaining copies as well as the breakpoints of the lost copy (marked with a green box).

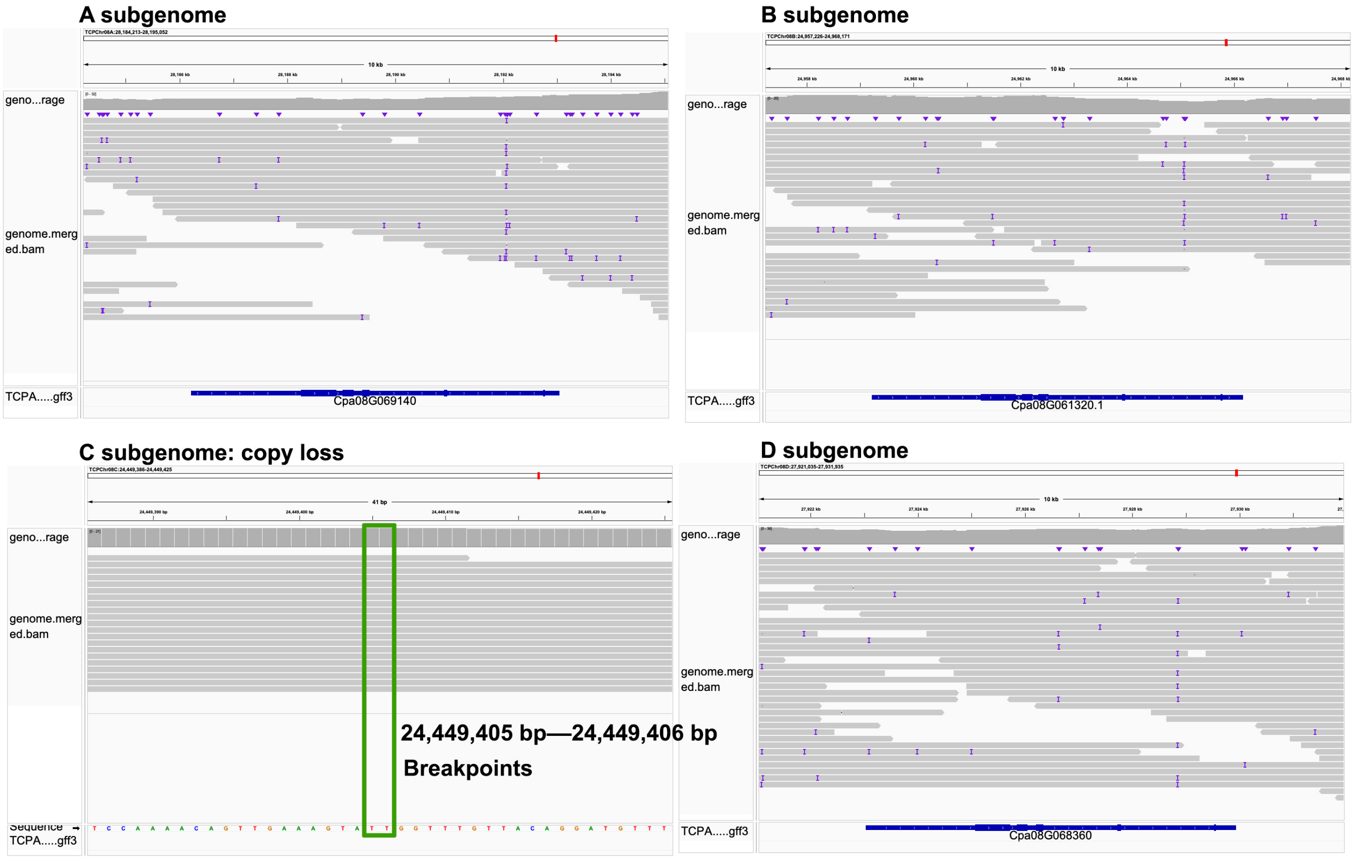

Example 2:

The locus lost one copy and now has three remaining copies: Cpa07G027120, Cpa07G025260 and Cpa07G026230. In the following figure, the HiFi reads cover the three remaining copies as well as the breakpoints of the lost copy (marked with a green box).

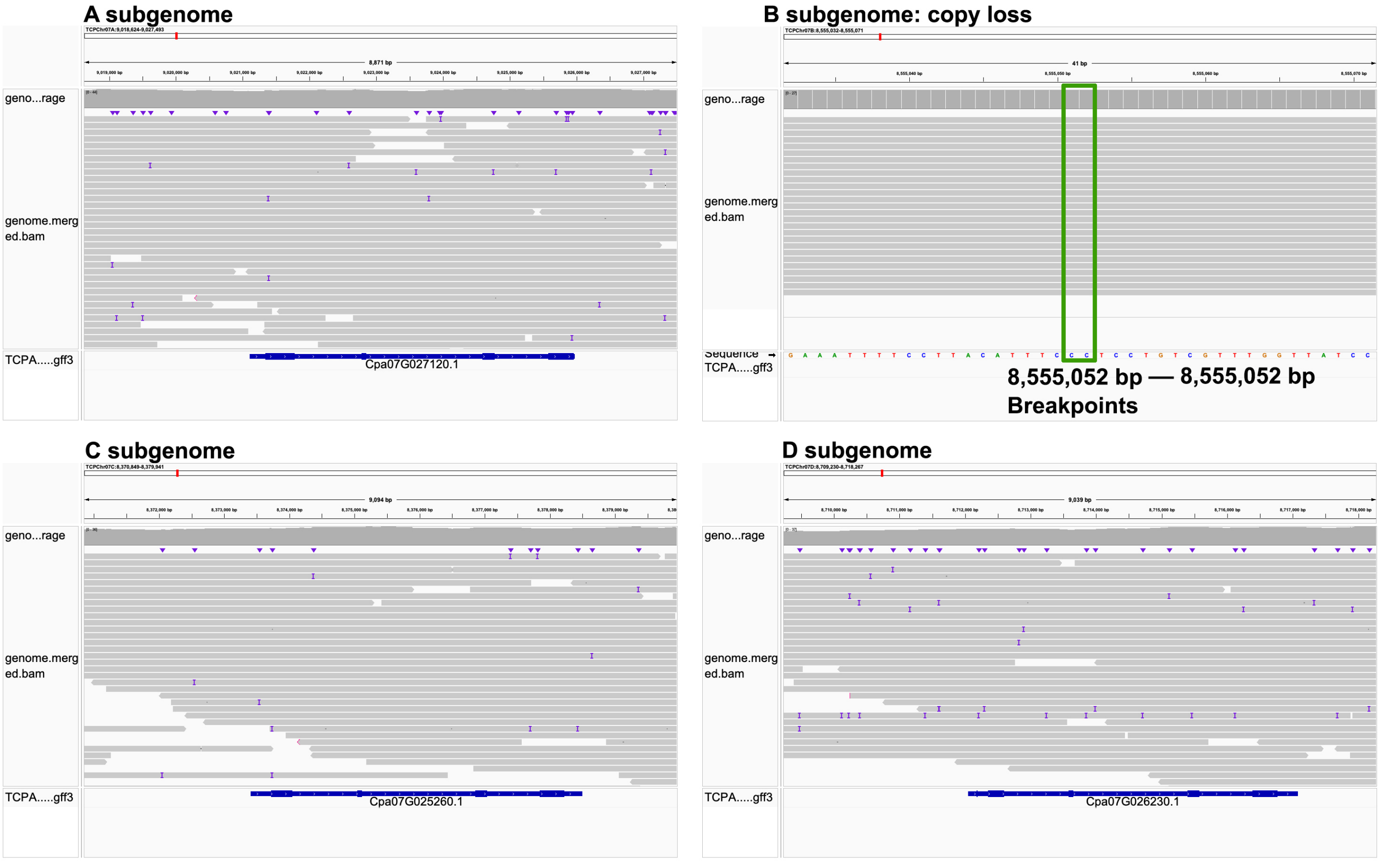

Example 3:

The locus lost one copy and now has three remaining copies: Cpa09G050810, Cpa09G055380 and Cpa09G052750. In the following figure, the HiFi reads cover the three remaining copies as well as the breakpoints of the lost copy (marked with a green box).

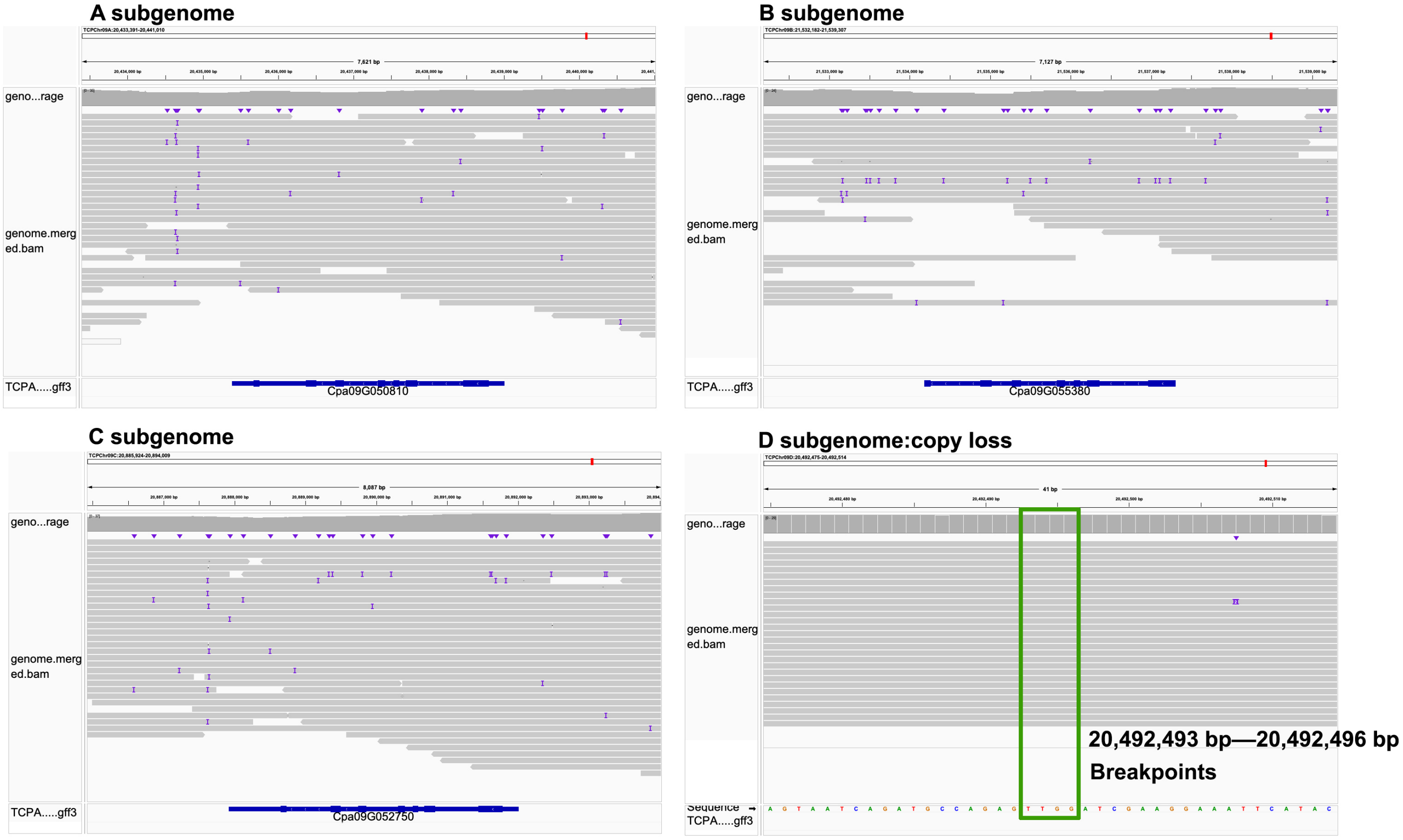

Example 4:

The locus lost one copy and now has three remaining copies: Cpa10G045070, Cpa10G045700 and Cpa10G046040. In the following figure, the HiFi reads cover the three remaining copies as well as the breakpoints of the lost copy (marked with a green box).

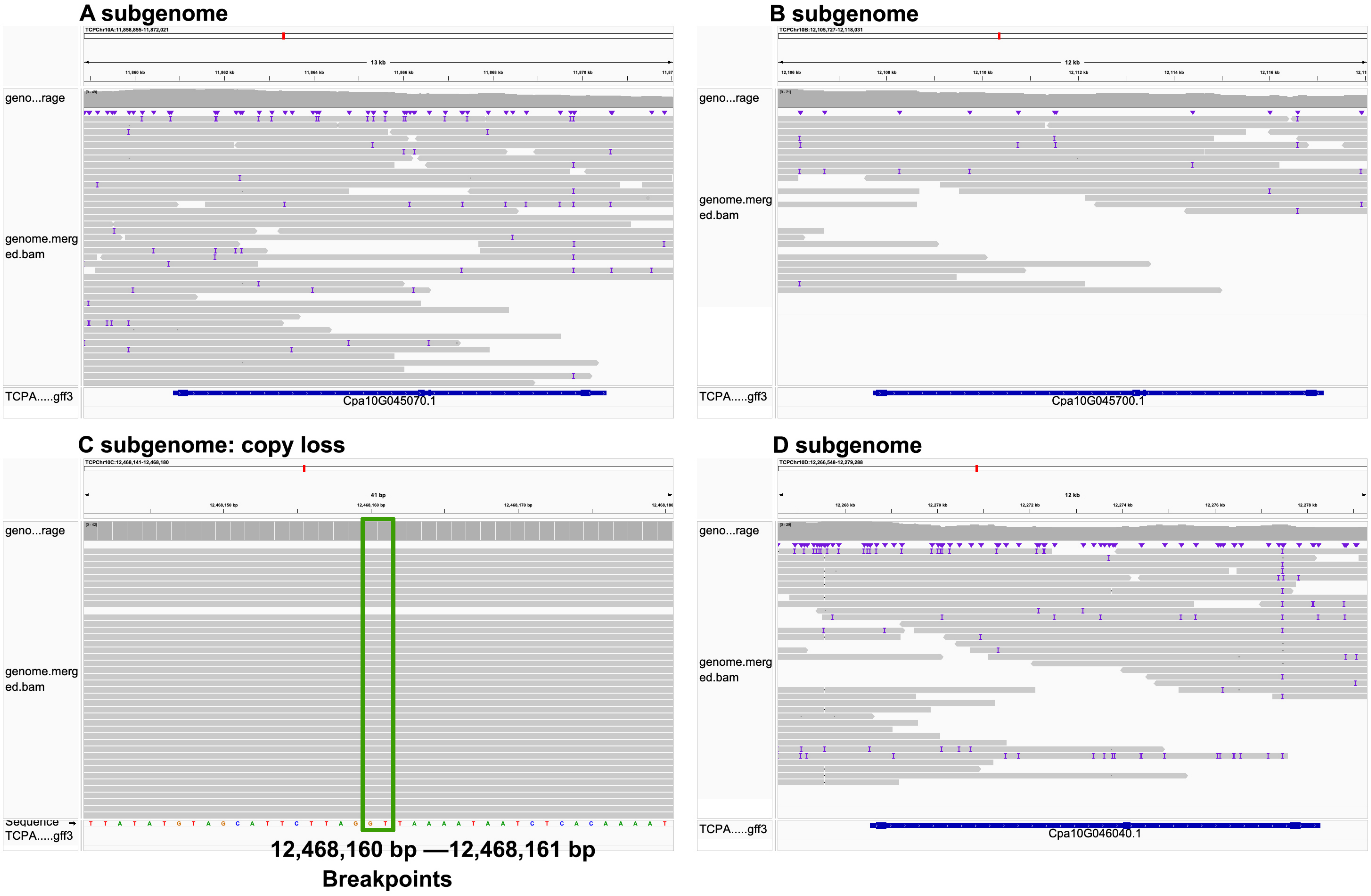

Example 5:

The locus lost one copy and now has three remaining copies: Cpa16G035520, Cpa16G035840 and Cpa16G036840. In the following figure, the HiFi reads cover the three remaining copies as well as the breakpoints of the lost copy (marked with a green box).

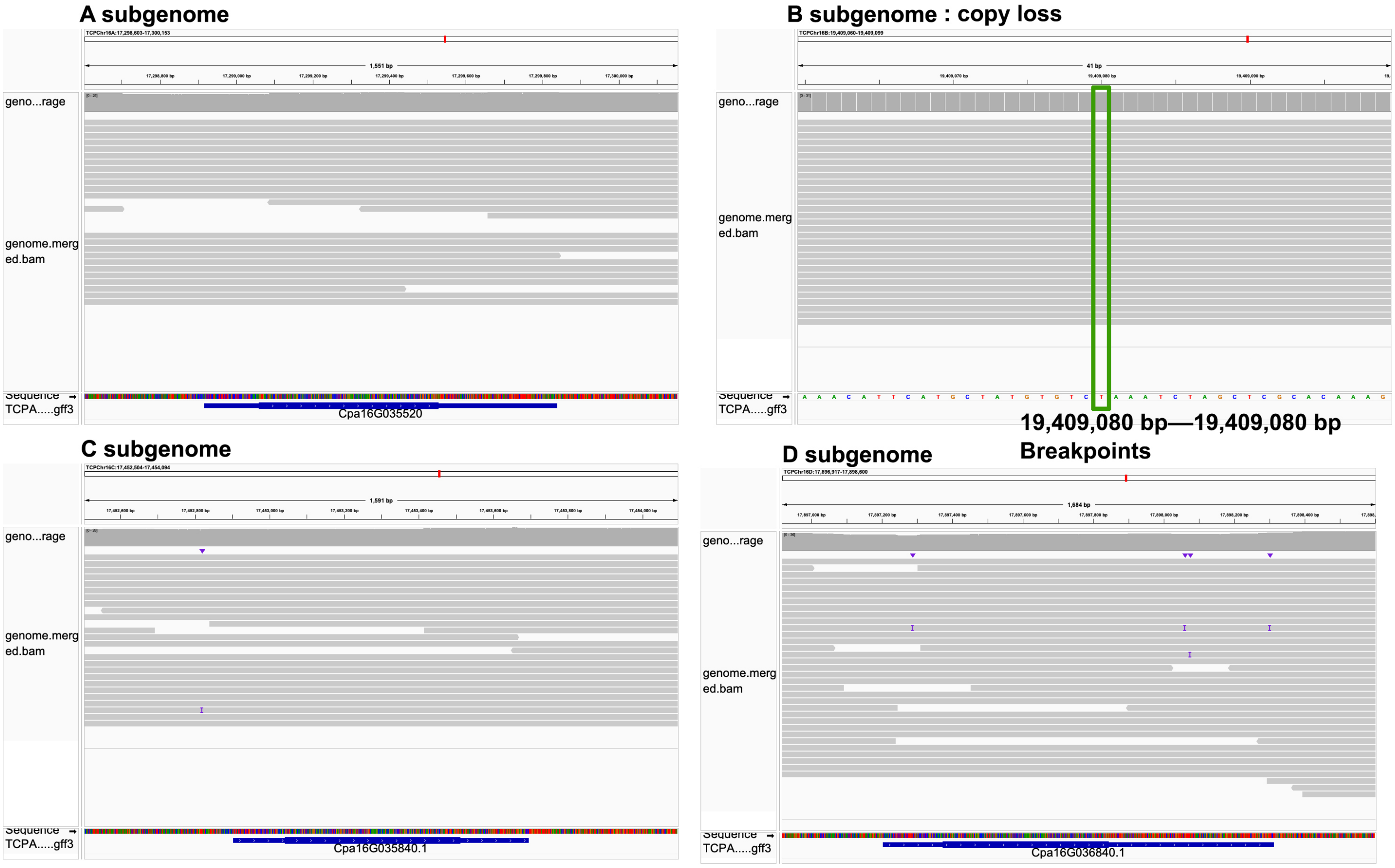

Example 6:
The locus lost one copy and now has three remaining copies: Cpa11G007480, Cpa11G008020 and Cpa11G007150. In the following figure, the HiFi reads cover the three remaining copies as well as the breakpoints of the lost copy (marked with a green box).

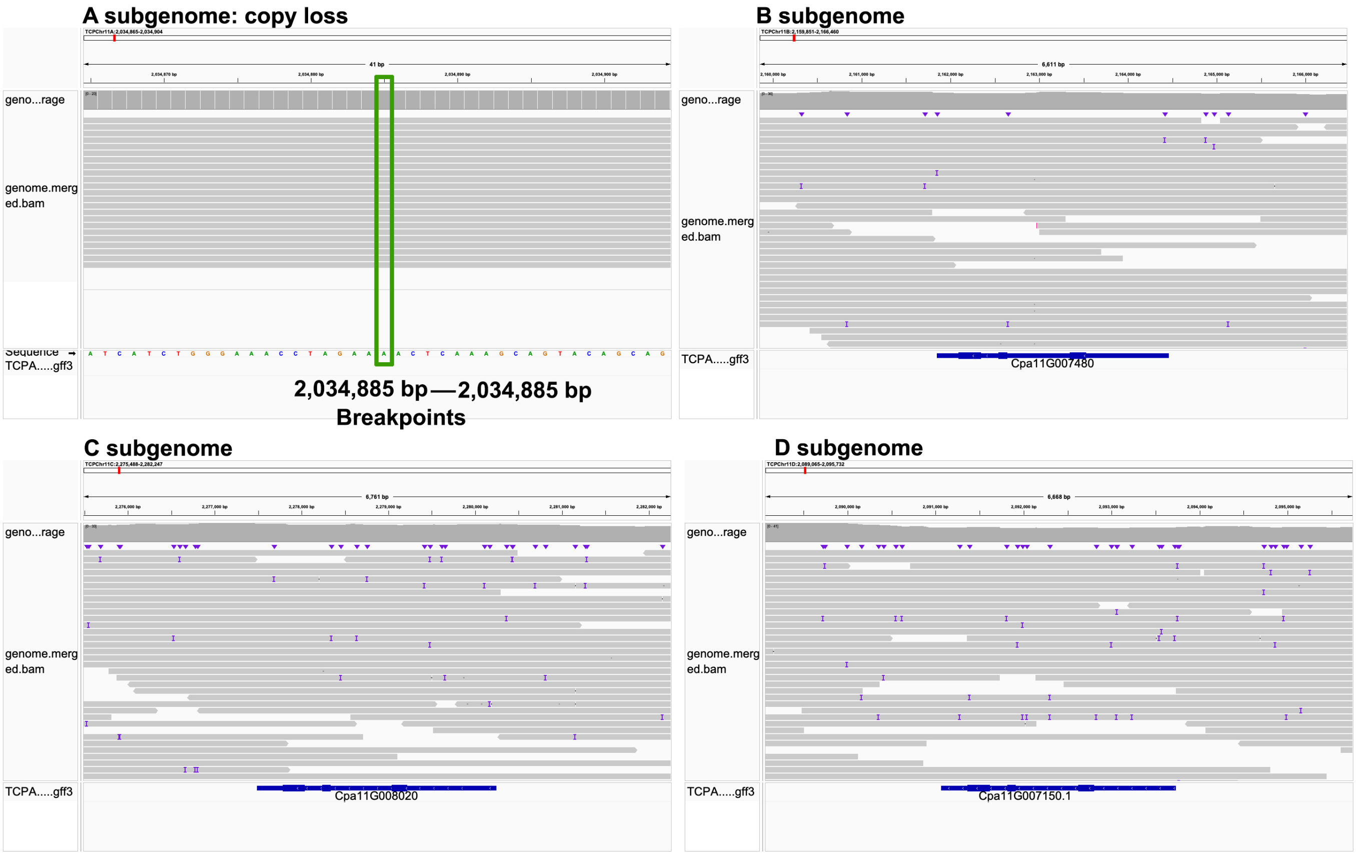

Example 7:

The locus lost one copy and now has three remaining copies: Cpa11G101910, Cpa11G097840 and Cpa11G078880. In the following figure, the HiFi reads cover the three remaining copies as well as the breakpoints of the lost copy (marked with a green box).

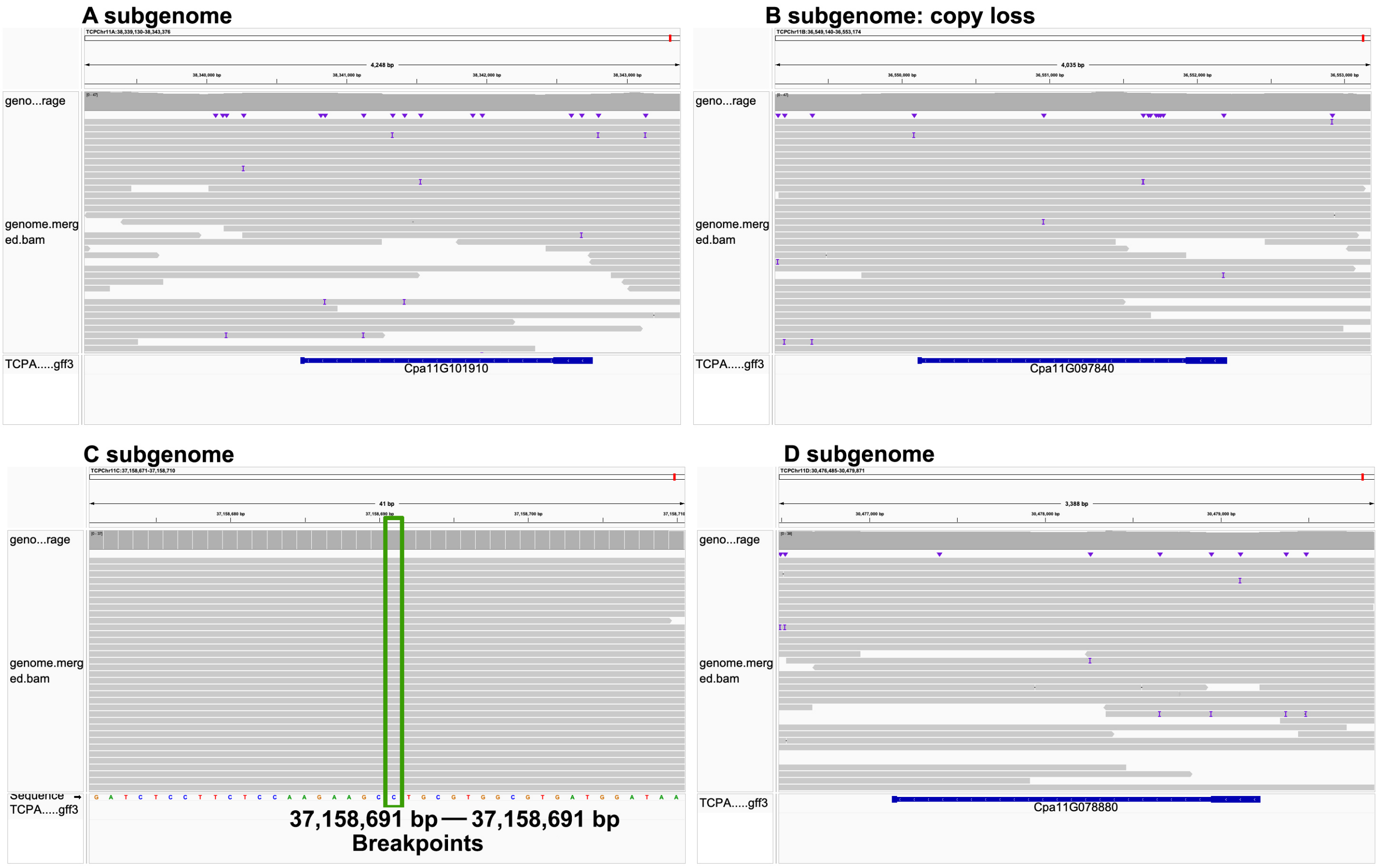

Example 8:

The locus lost one copy and now has three remaining copies: Cpa14G082490, Cpa14G081620 and Cpa14G079510. In the following figure, the HiFi reads cover the three remaining copies as well as the breakpoints of the lost copy (marked with a green box).

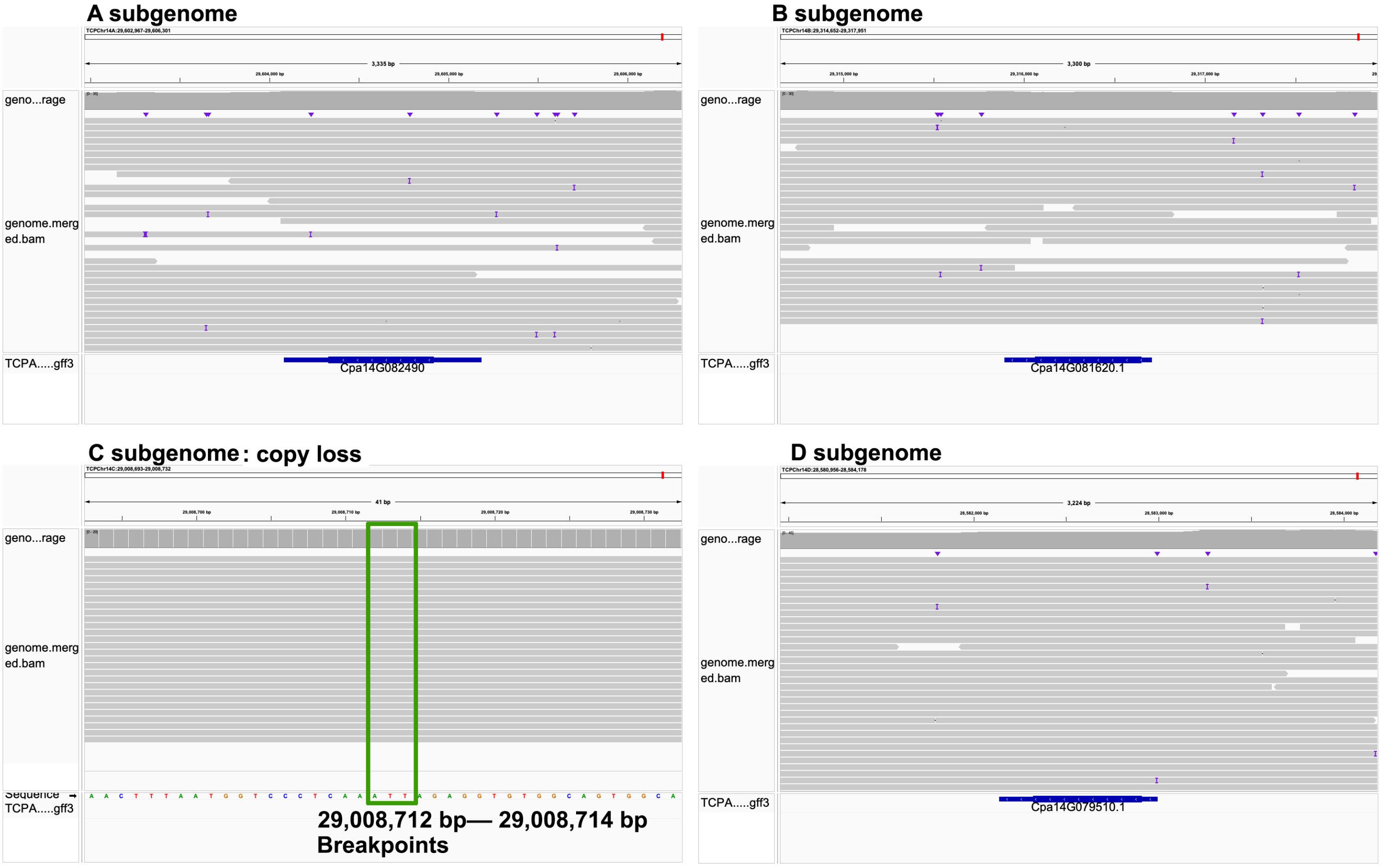

Example 9:

The locus lost one copy and now has three remaining copies: Cpa10G045070, Cpa10G045700 and Cpa10G046040. In the following figure, the HiFi reads cover the three remaining copies as well as the breakpoints of the lost copy (marked with a green box).

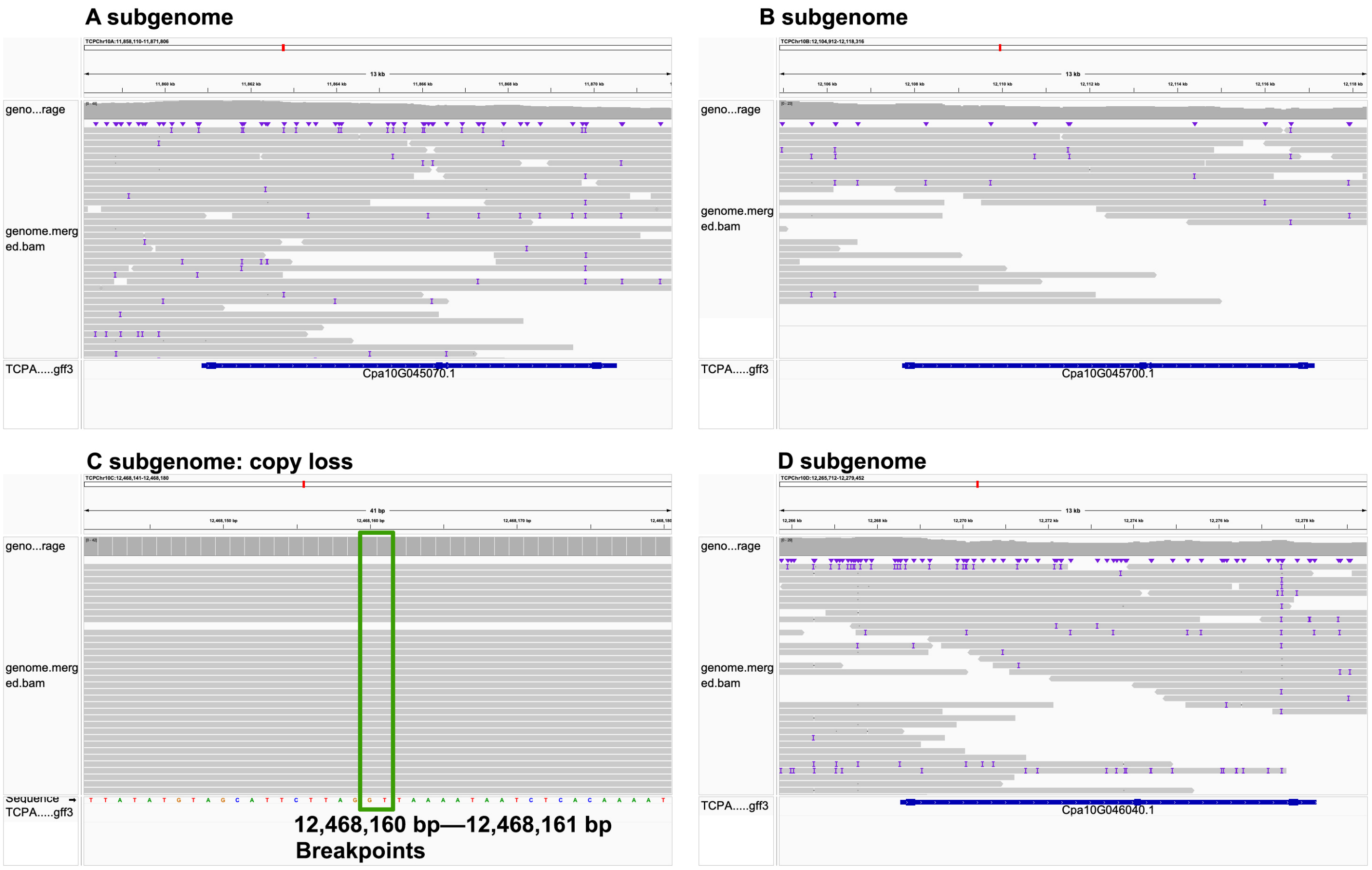

Example 10:
The locus lost one copy and now has three remaining copies: Cpa08G042260, Cpa08G037350 and Cpa08G043730. In the following figure, the HiFi reads cover the three remaining copies as well as the breakpoints of the lost copy (marked with a green box).

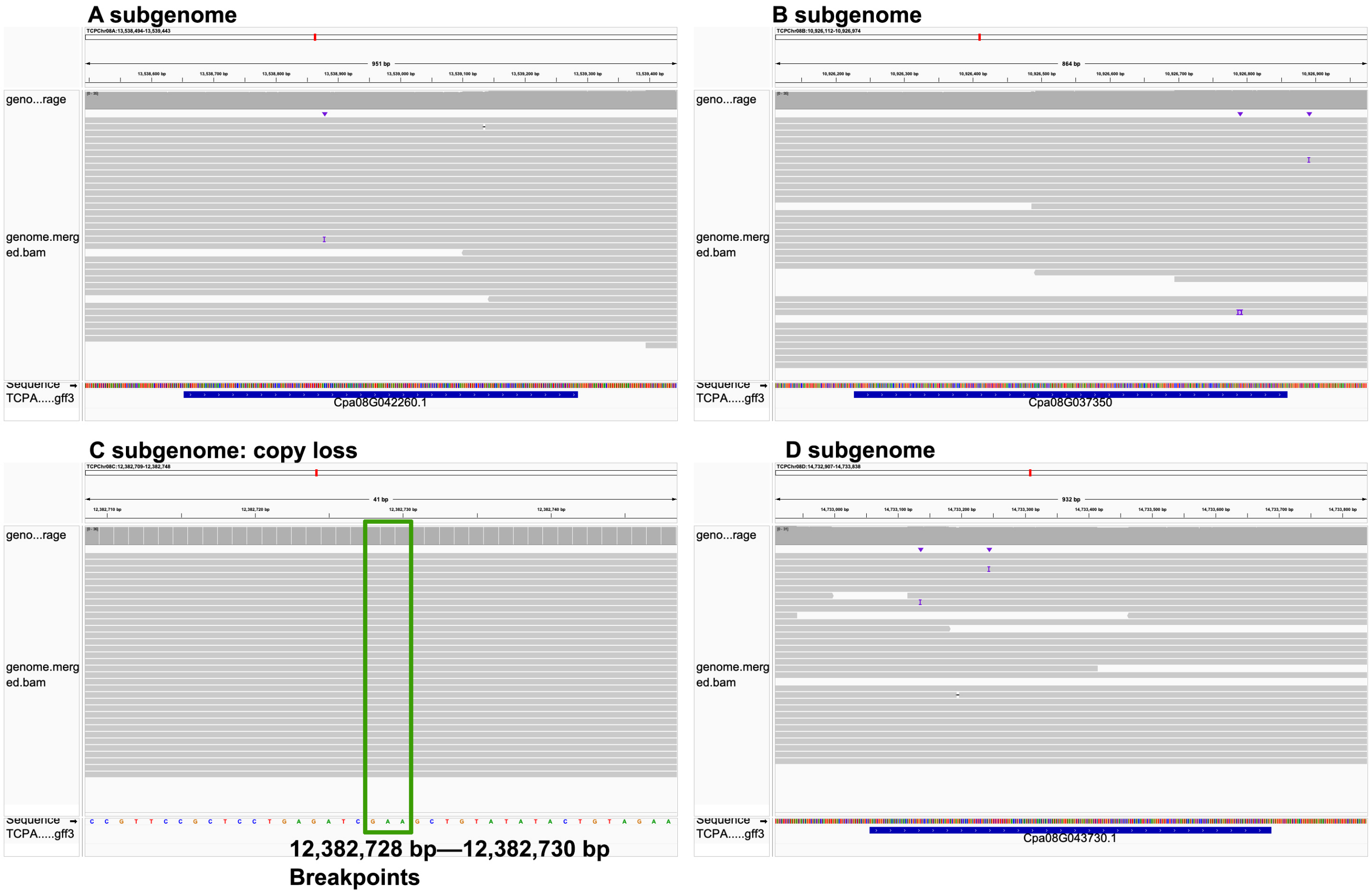

Supplement: Supplementary file 3 — Additional file 3. Instances copy gain and copy loss in autotetraploid C. paliurus. [file 12915_2023_1668_MOESM3_ESM.pdf]
