# Supplementary material for: Genomic insights into biased allele loss and increased gene numbers after genome duplication in autotetraploid Cyclocarya paliurus
Source: BMC Biol. 2023 Aug 8;21:168. doi: 10.1186/s12915-023-01668-1 (PMC10408227; doi:10.1186/s12915-023-01668-1)
Supplement: Supplementary file 6 — Additional file 6: Note 1. Genome assembly of diploid C. paliurus and P. stenoptera. Note 2. Genome annotation of C. paliurus and P. stenoptera. Note 3. Autotetraploid C. paliurus inheritance mode. [file 12915_2023_1668_MOESM6_ESM.pdf]

Genomic insights into biased allele loss and increased gene numbers after genome duplication in autotetraploid *Cyclocarya paliurus*

Rui-Min Yu[1], Ning Zhang[1], Bo-Wen Zhang[1], Yu Liang[1], Xiao-Xu Pang[1], Lei Cao[1], Yi-Dan Chen[1], Wei-Ping Zhang[1], Yang Yang[1], Da-Yong Zhang[1]*, Er-Li Pang[1]*, & Wei-Ning Bai[1]*

[1]State Key Laboratory of Earth Surface Processes and Resource Ecology, and Ministry of Education Key Laboratory for Biodiversity Science and Ecological Engineering, College of Life Sciences, Beijing Normal University, Beijing 100875, China.

*Corresponding authors: Da-Yong Zhang (zhangdy@bnu.edu.cn), Er-Li Pang (pangerli@bnu.edu.cn), Wei-Ning Bai (baiwn@bnu.edu.cn).

Note 1. Genome assembly of diploid *C. paliurus* and *P. stenoptera*.

For the assembly of the diploid genome, we utilized the hifiasm [75] software (v0.13-r308) to assemble the HiFi reads into contigs with the parameters set as -l2 -s 0.9 --high-het. To generate a deduplicated genome, we utilized Purge Haplotigs [79] with the 'contigcov' step parameters set to '-l 5 -m 70 -h 160 -j 80 -s 80' and 'purge' step set to '-a 60'. To generate a chromosome-level assembly, we employed 92 Gb of high-quality Hi-C data. The reads were aligned to the initial assembled genome using BWA v. 0.7.10 with default parameters, and only valid mapped read pairs were used to assemble the pseudo-chromosome sequences using ALLHiC [80]. The final chromosome assembly was obtained by manually ordering the scaffolds in each group with the assistance of Juicebox (https://github.com/aidenlab/Juicebox).

To assemble the P. stenoptera genome, we first corrected PacBio long reads and preassembled the genome using falcon-kit v. 1.0 [81]. The resulting assembly was then polished and checked for mis-assemblies using Arrow (SMRTLink, v. 5.1.0) with default parameters. To improve accuracy, we mapped Illumina 150 bp paired-end clean reads to the preassembled genome using the BWA-mem algorithm in BWA v. 0.7.10 [76] with default settings, and subsequently used Pilon v. 1.22 [82] to correct the preassembled genome. We further assembled the genome into chromosomes using 77 Gb of Hi-C reads, following the same method as for diploid genomes. The final P. stenoptera genome assembly, v. 3.0, had a size of 555.14 Mb with a contig N50 of 3.76 Mb. To assess the completeness of the assembly, we used the BUSCO database (http://busco.ezlab.org/) and CEGMA (http://korflab.ucdavis.edu/datasets/cegma/) and found that 92.5% of universal single-copy orthologs and 93.15% of ultra-conserved CEGs were present. We also identified 32,548 protein-coding genes and 3,649 noncoding RNA genes (1,698 rRNA, 636 tRNA, 375 miRNAs, and 940 snRNA) in the assembly, while approximately 52.27% of the assembly consisted of repetitive elements of the P. stenoptera genome (assembly v. 3.0).

Note 2. Genome annotation of *C. paliurus* and *P. stenoptera.*

To annotate repetitive sequences in the autotetraploid genome, we utilized a combination of homology-based and de novo methods. Initially, we used de novo method to identify transposable elements (TEs), which primarily involved TR_FINDER v. 1.1 [83] and RepeatModeler2 [84]. The latter software integrated several de novo methods, including RepeatScout v. 1.0.6 [85], RECON v. 1.0.8 [86], LTRharvest v. 1.5.9 [87], and LTR_retriever v. 2.8 [88]. LTR_FINDER was used to identify LTR-RTs with the following parameter settings: '-D 40000 -d 100 -L 9000 -l 50 -p 20 -C -M 0.9'. To identify the TEs, we also utilized the existing RepBase library (Bao et al., Mobile DNA. 2015, 6:11). Finally, we identified and categorized TEs in the autotetraploid genome by performing a homology search against the library using RepeatMasker v. 4.10 [89]. We also detected tandem repeats using TRF v. 4.09 [90] and MISA v. 2.1 [91].

An integrated approach was employed to annotate protein-coding genes in the genome, which involved de novo prediction, homology search, and transcriptome-based prediction. Two ab initio gene-prediction softwares, Augustus v. 2.4 [92] and SNAP (v. 2006-07-28) [93], were used to predict de novo gene models. GeMoMa v. 1.7 [94] was utilized for the homolog-based approach, which predicted genes with reference gene models from five other species (*J. regia*, *J. nigra*, *J. mandshurica*, *A. thaliana*, and *V. vinifera*). The transcriptome-based prediction was performed by mapping RNA-sequencing data to the reference genome using Hisat v. 2.0.4 [95] and assembling them by Stringtie v. 1.2.3 [96]. Assembled transcripts were also used for gene prediction by GeneMarkS-T v. 5.1 [97], while unigenes assembled by Trinity v. 2.11.0 [98] were used for gene prediction by PASA v. 2.0.2 [99]. Gene models from these different approaches were combined using the EvidenceModeler (EVM) v. 1.1.1 [100] and updated by PASA. The final gene models were annotated by searching various databases, including NR (202009, https://ftp.ncbi.nlm.nih.gov/blast/db/), GO (20200615, http://geneontology.org), KEGG (20191220, http://www.genome.jp/kegg), Pfam (v. 33.1, http://pfam.xfam.org), SWISS-PROT (202005, http://ftp.ebi.ac.uk/pub/databases/swissprot), and EggNOG (v. 5.0, http://eggnog5.embl.de/download/eggnog_5.0). A total of 1,610 (99.75%) genes were mapped to the genome in BUSCO evaluation. tRNAscan-SE v. 1.3.1 [101] was used to predict tRNA genes with eukaryote parameters, while barrnap v. 0.9 [102] was used to identify rRNA genes. miRNA genes were identified by searching miRbase [103] (release 21) databases. snoRNA and snRNA genes were predicted using Infernal v. 1.1 [104] against the Rfam v. 12.0 [105] database.

For diploid genome, TRF v. 4.09 was used to identify tandem repeats. TEs of the diploid genome were annotated by the combination of homology-based and de novo approaches. Alignment searches were undertaken against the RepBase database (http://www.girinst.org/repbase/), and then were predicted by RepeatMasker v. 4.10.

For de novo annotation, LTR_FINDER and RepeatModeler2 were used to construct a de novo library, and then annotation was carried out with RepeatMasker2.

We utilized a similar strategy as that used for the autotetraploid genome to annotate protein-coding genes in the diploid genome. For de novo, we employed five prediction programs to predict the coding regions in the repeat-masked genome (de novo set), namely Augustus v. 2.4, Genscan v. 1.0 [106], Geneid [107], GlimmerHMM v. 3.0.1 [108] and SNAP v. 2006-07-28. For homology annotation, we selected protein sequences from six different species (*J. regia*, *P. stenoptera*, *Carya cathayensis*, *Morella rubra*, *Quercus lobata*, and *Ostrya rehderiana*) to predict protein-coding genes using GeneWise [108] (Homo-set). To obtain annotation results based on transcripts, RNA-seq alignment files were generated using Tophat v. 2.0.8 [109], which were then assembled into gene models (Cufflinks-set) via Cufflinks v. 2.1 [109]. Furthermore, we used PASA to align spliced transcripts and annotate candidate genes (PASA-T-set). Finally, we merged gene models predicted from the four sets using EVM v. 1.1.1, resulting in a non-redundant set of gene annotations. To identify the functions of protein-coding genes, we mapped their sequences against six databases, namely SwissProt, NR, KEGG, InterPro (https://www.ebi.ac.uk/interpro/), GO, and Pfam. We annotated genes for tRNA, miRNA, and snRNA using tRNAscan-SE v. 1.3. and Infernal v. 1.1, while rRNA sequences were predicted by aligning them to Arabidopsis template rRNA sequences.

It is worth noting that the annotation for *P. stenoptera* was similar to that of the diploid *C. paliurus*.

Note 3. Estimation of mode of inheritance in autotetraploid *C. paliurus*.

Both disomic and tetrasomic inheritance may happen in autotetraploids, so we compared our observed data with different simulated data to determine the inheritance mode of autotetraploid *C. paliurus*. We followed the methodology described in Hollister et al [49] to generate expected neutral SFS under both tetrasomic and disomic inheritance model.

For six simulations, we set the sample size to 48 chromosomes and the theta (-t switch) was equal to the theta estimated from our data (0.084). To model disomic inheritance, we simulated two isolated sub-populations for different time by selecting two chromosomes per individual (representing the two homologous chromosome pairs). We set td from 1 to 0.2, stepping by 0.2 to represent the divergence time decreased sequentially. For td=0.2, we set -I switch '2 24 24' to specifies two sub-populations of sample size 24, set -ej switch '0.2 1 2' to specify all the lineages in subpopulation 1 moved to subpopulation 2 at time 0.2, set '-G 8.7' to specifies that the population decreases as we go back in time, set '-eG 0.2 0.0' to specifies that the growth rate changes to zero at time 0.2. We used the '-G' and '-eG' to account for the population decreasing in our observed data as we move back in time. Similarly, we changed '-ej' switch to '0.4 1 2', '-eG' switch to '0.4 0.0' for td=0.4, and other parameters were set the same as td=0.2. The same simulations were performed for td = 0.6, 0.8 and 1.0.

Furthermore, we investigated the extent of disomic inheritance on tetraploid genomes by conducting simulations at different proportions of disomic inheritance, ranging from 10% to 90%, stepping by 10%. For example, assuming the tetraploid genomes that display 10% disomic inheritance, we can break down this 10% into five equal parts of 2%, each corresponding to Td values of 0.2, 0.4, 0.6, 0.8, and 1.0.

For our observed data, we selected 12 tetraploid individuals from our dataset and obtained a total of 60,578,248 SNPs. We filtered out missing SNPs, SNPs with less than 1/3 or greater than 2 times the average depth and biallelic SNPs, resulting in 30,124,148 high-quality SNPs that were utilized to create the observed SFS.

To statistically evaluate the distribution of SFS between our observed and simulated data, we employed the Two-Sample Kolmogorov-Smirnov test, which is considered more powerful in assessing the goodness of fit of a theoretical distribution to observed data [139].
